# The role and impact of therapeutic counselling on the emotional experience of adults living with dementia: A systematic review

**DOI:** 10.1177/14713012241233765

**Published:** 2024-04-16

**Authors:** Gill Mathews, Xiaoyang Li, Heather Wilkinson

**Affiliations:** Edinburgh Centre for Research on the Experience of Dementia (ECRED), 65932 School of Health in Social Science, University of Edinburgh, UK

**Keywords:** counselling, psychotherapy, emotional well being, systematic review, psychological support, mental health, dementia

## Abstract

Introduction There is limited psychological support available to help people living with dementia to deal with the emotional consequences of their condition. Anxiety and depression are commonly experienced in this population, yet the use of counselling and psychotherapeutic interventions is not well documented. Aim This systematic review sought to understand the current knowledge on the role and impact of therapeutic counselling on the emotional experience of adults living with dementia. Methods Qualitative and quantitative research designs were accepted for review. A comprehensive search of the main biomedical, nursing and other specialist databases was performed to access articles published between 2015 and 2022. Trial registers and academic journals were also searched. 43 original studies were included: qualitative (*n* = 15); RCTs (*n* = 9); other designs (*n* = 19); plus eight systematic reviews. Results The majority of studies were conducted in Europe, the United Kingdom in particular, although a range of countries from across the globe were represented. The combined evidence from the different study designs suggest a range of ways that people living with different stages of dementia can participate in, and gain emotional benefit from, therapeutic counselling. Key themes identified: (1) The emotional and well-being benefits of therapeutic counselling; (2) No one size fits all - relational and tailored approaches driven by person-centred values; (3) Training, supervision and building community for counsellors; (4) Involvement of people with dementia in therapeutic interventions. Conclusions Our findings from this systematic review show that different therapeutic approaches have been tested with people at different stages of a dementia diagnosis. The results suggest the value of therapeutic counselling as a supportive medium to help with the processing and coping of difficult emotions and feelings across the trajectory of a dementia illness.

## Background

Dementia is a complex neurological condition that damages nerve cells and diminishes a person’s physical, cognitive and emotional capacities (World Health Organisation, 2020). Someone in the world develops dementia every 3 seconds (Alzheimer’s Disease International, 2023). Approximately 50 million people live with dementia globally and the estimated proportion of the general population aged 60 and over with dementia at a given time is between 5%–8% (World Health Organisation, 2019). Increasingly, people are receiving a dementia diagnosis at an earlier stage of the disease process when they are more likely to be able to talk freely about its impact on their mental health (Vernooij-Dassen et al., 2006). The diagnostic experience is, however, often not well managed by health professionals and comorbidities are often underdiagnosed (Regan, 2016).

Practice and research on emotional well-being for those living with dementia continue to lags behind the main focus on drug therapy (Alzheimer’s Society, 2019). The rate of depression for people living with vascular dementia may be 30% and in other dementias, over 40% (Enache et al., 2011). Studies suggest that prevalence of depression for people living with vascular dementia may be 30% and in other dementias, over 40% (Enache et al., 2011). In care homes, it is likely that up to 60% of residents experience poor mental health of whom 40% experience depression (Pybis et al., 2021). Anxiety symptoms are also clinically significant in one third of elder care home residents (Creighton et al., 2016). Both depression and anxiety have a substantial impact on the presentation and incidence of behavioural and psychological symptoms (Koder, 2016), affecting individual overall quality of life and the nature of care management. Although pharmacological treatments are largely ineffective in this context and carry adverse risk, these are frequently given to people living with dementia (Banerjee et al., 2011; Wang et al., 2018).

Addressing the cognitive or medical impacts of dementia is often the key focus of research whilst the exploration of difficult emotional experiences remains largely neglected (Poz, 2018). The experience of dementia can elicit many different feelings including an increased sense of vulnerability and fear around loss of control. People commonly report anxiety following a diagnosis of dementia and stress and anxiety can intensify as they encounter different life transitions throughout the disease trajectory (Eriksen et al., 2018). The emotional needs of people living with later stages of dementia are also important, yet rarely addressed, pointing to a role for a nuanced and tailored therapeutic approach that is responsive to individual needs across the dementia trajectory. Without adequate support, the individual may be left feeling confused and grief-stricken resorting to avoidance as a key coping strategy (Milby et al., 2015).

Medical and nursing care in the biomedical model focuses on reducing the advance of the disease and symptom management but can overlook the ‘felt’ sense of a person’s experience and their human right to be as they are (Cahill, 2018). Counsellors and psychotherapists can play a crucial role in easing psychological distress and help with future decision-making and referral on to other supportive provisions, where indicated. Counselling is described as a helping therapy. Usually, it involves talking to a trained professional and sharing on thoughts, feelings and emotions (Sanders, 2011). The term can, however, be misconstrued as it is used in a variety of contexts other than therapeutic, for example in advocacy and advice-giving (Aldridge, 2014). A key purpose of ‘therapeutic’ counselling is to improve emotional and mental well-being through building a therapeutic relationship, creating a safe and confidential space in which clients can self-express and create meaning out of their personal experience or circumstances (Norcross, 2010).

A common view among mental health practitioners and therapists has been that people with memory problems cannot benefit from counselling (Hepple, 2004; Bruce-Hay et al., 2011; Hill and Brettle, 2005). There is mounting evidence, however, to challenge this standpoint and demonstrate the potential for counselling interventions at different stages of the dementia (White et al., 2018). Project work in the Camden area of London, for example, returned positive impacts from a year of counselling (Patrikiou, 2018). Benefits experienced by people living with dementia included increased communication and relatability, improved mood and cognitive functioning, and greater self-expression. Within the nursing home environment, explicit staff training in counselling skills/facilitative communication has also empowered residents with advanced dementia (Alnes et al., 2011). Similarly, research by Watson (2016) demonstrates ongoing engagement within relationships until the end of life.

Counselling and psychotherapy have multiple formats. Mostly these take the form of talking therapies and reflect a recognised theoretical premise, such as humanistic approaches include Person Centred Counselling (PCC) (Mearns and Thorne, 2013; Rogers, 1951), Rational Emotive (REBT) and Cognitive Behavioural Therapies (CBT) (Beck, 1979; Beck & Emery, 1985; Ellis, 1977) and Mindfulness Based interventions (MBI). Currently there is little readily available evidence appraising the role and value of different counselling methods for people living with dementia. Scholarly texts on practitioners’ experiences of counselling people living with dementia imply high value (Cooper, 2008; Holland, 2012). However, the research evidence on counselling and the therapeutic relationship in this context is not well developed.

This critical gap in the knowledge calls for a detailed investigation to grow our understanding about counselling interventions and their modes of delivery to ascertain which may offer the greatest benefit. This systematic review sought to search out and understand different perspectives on the role and impact of therapeutic counselling on the emotional experience of adults living with dementia, and to develop insights into relevant process issues. Our findings contribute to an evolving theoretical knowledge of the potential for counselling people who live with dementia and offer guidance for the future implementation and delivery of counselling services in this context.

## Why it was important to conduct this review

Many people living with dementia and their families do not have the chance to talk about how they feel (Regan, 2016). Evidence on their views has generally been absent from research, policy and practice that concerns them (IInnes and Manthorpe, 2012). This situation is gradually changing but such oversight emphasises how the condition can be rendered invisible and highlights the critical importance of bringing the specific psychological needs of people living with dementia into clear awareness.

To develop and improve access to counselling services more knowledge is needed - in particular, to understand the perspectives and experiences of people living with dementia who have undergone counselling and therapist’s experience of working in this context. Such data will build insight into the key issues of importance in respect of day-to-day coping with the psychological impacts of dementia, user views on counselling, and greater awareness of the type of services that might work best for them. Much of the current social science research literature is rooted in quantitative approaches measuring such factors as dementia severity, the cost of care and caregiver burden, using methods that fail to recognise the person behind the condition (Cahill, 2018). Here, reflecting the recommendations of Cohen-Mansfield et al. (2014) we adopted a broad approach to study design to help gather and evaluate relevant information on the critical issues and needs around the topic, giving particular attention to the views expressed by people living with dementia who had undergone counselling therapy.

## Review question

What is the current knowledge on the role and impact of therapeutic counselling on the emotional experience of adults living with dementia?

## Methods

### Search methods for identification of studies

A five-part search strategy identified eligible studies published since 2015 which met the inclusion criteria. This comprised searching: (1) electronic bibliographic databases for published work, using a comprehensive search strategy for therapeutic counselling interventions; (2) trial registers for ongoing and recently completed trials; (3) the reference lists of original research studies included in the review; (4) prominent journals reporting on dementia and counselling/psychotherapy domains; 5) the grey literature.

The first two review authors documented the search process and the records, assessed potentially relevant studies and made the final selection for inclusion and data extraction. Any disagreements were resolved by discussion.

### Electronic searches

A comprehensive search of the main biomedical, nursing and other specialist databases listed in Table 1 was performed to access articles published between 2015 and 2022:

**Table 1.** Databases searched.

AMED
CINAHL (Cumulative Index to Nursing and Allied Health Literature)
Cochrane Central Register of Controlled Trials (CENTRAL, The Cochrane Library)
EBSCO – ERIC, Family and Society Studies Worldwide
EMBASE
Global Health
MEDLINE
PsychArticles
PsycINFO
Pubmed, Social Policy and Practice, PROQUEST – ASSIA, British Nursing Index, Social Services Abstracts, Sociological Abstracts
PubMed
SPORT DISCUS
University of Edinburgh Library (Discover Ed)
WEB of SCIENCE

A free-text search of titles, abstracts, and keywords, as well as medical subject headings (MeSH) during searches, was implemented. The search ran from 2015 to December 2022 with terms adapted to individual databases. The full search strategy for MEDLINE is contained in Appendix 1. To search for controlled trials of interventions, the terms were combined with the relevant Cochrane Library MEDLINE filter. The counselling and dementia search terms were consequently used for the other bibliographic database searches, with modifications applied to suit. 

### Searching other resources

We searched these trials registers: World Health Organisation (WHO) International Clinical Trials Registry Platform (ICTRP) (https://apps.who.int/trialsearch/) WHO clinical https://trials.gov, clinicaltrials.gov and clinical https://studies.org; and reviewed the following specialist journals: (1) Dementia, an international peer reviewed journal that acts as a major forum for social research of direct relevance to improving the quality of life and quality of care for people living with dementia and their families; (2) Alzheimer’s and Dementia which covers and seeks to connect the wide spectrum of dementia research; (3) Counselling and Psychotherapy; (4) Counselling and Psychotherapy Research; (5) Journal of Ageing and Mental Health; (6) Psychotherapy Research. Expert authors were contacted for information and clarification where required.

### Selection criteria

The studies included in the review are outlined in Table 2 below.

**Table 2.** Inclusion criteria.

Criteria	Inclusion	Exclusion
Population	Eligible participants were aged 18 years and older with a diagnosis of dementia. This included those where people living with dementia formed part of a mixed population, as long as data could be extracted separately for people living with dementia. Professional perspectives relevant to counselling people living with dementia were drawn from research studies involving practitioners with a legitimate qualification in their field, e.g. counsellors, psychotherapists, specialist nurses	Studies that reported exclusively on rare forms of dementia were excluded, such as people with human immunodeficiency virus, amyotrophic lateral sclerosis, creutzfeldt–Jakob disease, Huntington’s disease, Parkinson’s disease and Down’s syndrome were excluded as these populations may require specific support due to the particular type of psychological challenges they face (Cheston & Ivanecka, 2017) Participants with mild cognitive impairment (MCI) were excluded
Intervention	Interventions included in the review concentrated on improving the emotional well-being of adults living with dementia through the use of some form of therapeutic counselling. Included interventions centred on the helping relationship as a means of supporting participants living with dementia to bring about positive mental, emotional and/or behaviour change. These comprised either: (1) counselling and psychotherapeutic interventions; or, (2) the user and/or professional perspective on these	Following Cheston and Ivanecka (2017), we excluded studies where the main intervention focus was art therapy, cognitive training, stimulation or rehabilitation, life review and reminiscence therapy
Outcome	These contained subjective, objective and self-reported measures that reflected our primary outcome: Changes in emotional health and well-being status following participation in a therapeutic counselling intervention placing focus on the user perspective We also searched for secondary outcome evidence relating to: the use of different counselling and psychotherapeutic modalities and their modes and settings of delivery; unintended or adverse consequences of the interventions; and, professional perspectives on the role of therapeutic relationships for people living with dementia	
Study types	We included original research articles that report on counselling or psychotherapeutic counselling for people living with dementia. Qualitative and quantitative research designs were accepted for review. The latter included randomised control trials (RCT) and observational studies. A small number of case studies of relevance to the review aim, and theoretical perspectives, as provided by systematic reviews were also integrated	
Language	Due to resource constraints, only articles published in the English language were included	

## Data collection and analysis

### Selection of studies

Following the search strategy and inclusion criteria, titles and abstracts of studies retrieved from the databases were screened independently by the first and second review authors. The saved abstracts were downloaded into the reference manager Endnote X8 and duplicate records removed. The full-text documents of those studies marked as potentially relevant were obtained and checked for eligibility using standardised proforma informed by the CASP quality appraisal system (Critical Appriasal Skills Programme, 2019). Any differences were resolved through author discussion.

### Data extraction and management

The data were independently extracted from the included original research studies by the first and second review authors and organised within a standardised format to enable assessment of study quality and evidence synthesis. Characteristics of included studies were then tabulated according to study design. Extracted information included: author/date and country; study aim; participant characteristics (age range, gender, ethnicity, type of dementia, severity of dementia), setting and context; intervention details (modality/therapist/setting/timing and duration); study design; data collection methods; outcome measures; attrition; results; key findings/themes; and, recommendations.

### Quality appraisal and assessment of risk of bias in included studies

Quality rating of the different types of study (Supplement Table 3) was conducted by two of the review authors using a recognised method and scoring system (CASP) to assess evidence for use in healthcare practice and policy development (Critical Appriasal Skills Programme, 2019).

### Statistical analysis

A meta-analysis was not undertaken. Over seventy different outcome measures were recorded, an outcome that diminished the potential to compare and contrast the statistical results. It also highlighted a need for researchers to identify and reach agreement on a more unified approach for the use of quantitative outcome measures in future research on dementia counselling. Such a move would facilitate a more direct comparison of key markers.

### Data synthesis

Outcomes were classified by the review authors with the following characteristics detailed:

• Summary of the key components of original studies retrieved from the review
• The type of counselling or psychotherapeutic intervention, for example Person Centred Counselling, psychodynamic therapy, and their key features/components
• The mode of delivery, for example individual, group, dyad, face to face, online
• The outcomes of the intervention placing emphasis on the perspective of the person with dementia (and family members/caregivers)
• Any adverse consequences of the interventions and process outcomes
• Professional perspectives on the role of therapeutic relationships/counselling for people living with dementia

## Results

**Figure 1.**
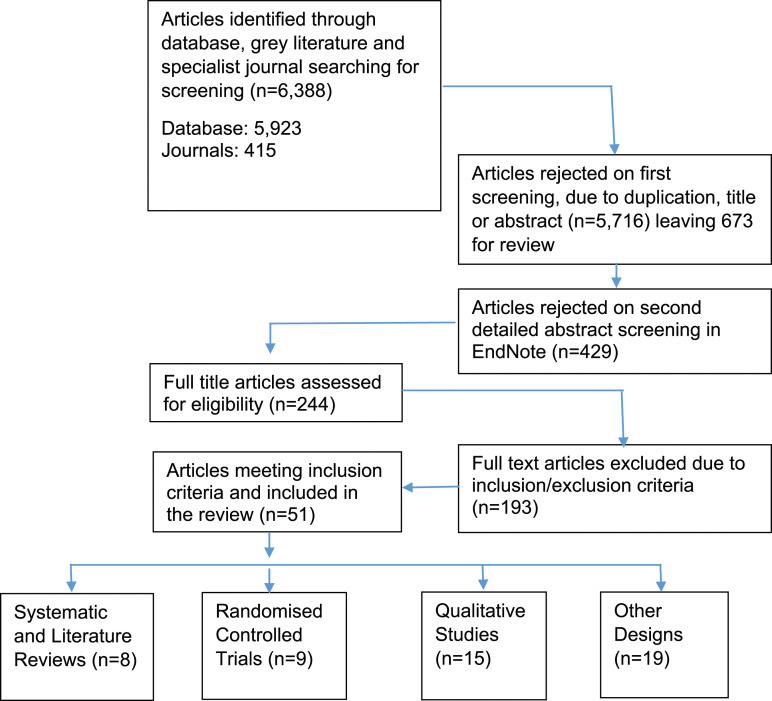
Counselling People with Dementia: PRISMA flow chart (Moher et al., 2009).

After titles and abstracts screening and further review of the full texts, 51 articles met the inclusion criteria: 43 original studies plus eight systematic reviews. A PRISMA study-flow diagram (Figure 1) documents the screening process (Schünemann et al., 2011). The process also elicited relevant grey literature which helped to contextualise the review findings at the discussion stage. Excluded articles are listed within Appendix 2.

### Summary of original studies published between 2015 and 2022 and included in the review

Detailed information extracted from the original research studies (*n* = 43) and systematic reviews uncovered in the review is summarised in Supplemental Tables 4, 5, 6 and 7.

#### Study design and aims

All of the original articles reviewed cited a study aim or objective(s). Thirty-four of these expressed aims specific to the emotional well-being of people living with dementia whilst in the remainder (*n* = 9) this comprised a secondary consideration. Nine of the interventions were Randomised Controlled Trials (RCT). Of these, three were pilot studies that employed a single group design. Fifteen qualitative articles were included. The remainder comprised a range of designs including feasibility or pilot (*n* = 12); survey design (*n* = 4); case report (*n* = 2) and one participatory action research/developmental study.

#### Longitudinal

The longer-term impacts of counselling people living with dementia were evaluated in six studies (Cornelis et al., 2018; Koivisto et al., 2016; Lykkeslet et al., 2016; Spector et al., 2015; Tonga et al., 2016, 2021). Findings reveal mixed results. Highlighted, were beneficial improvements to: mood (Spector et al., 2015; Tonga et al. 2016, 2021); quality of life and behaviour (Cornelis et al., 2018; Lykkeslet et al., 2016). Although some studies involved post intervention follow-up for research purposes, the highest number of psychological interventions delivered to participants totalled 12 sessions.

#### Sample

Overall, people living with dementia (*n* = 1,044) directly participated in research across the 43 original studies. Indirect participation through data gleaned from survey designs involved a further 1,000 participants living with dementia. Sample sizes including people living with dementia varied between (*n* = 1) and (*n* = 797), the latter comprising retrospective, survey data (Jennings et al., 2016).

#### Participant characteristics

Occurring diagnoses were: Alzheimer’s disease (AD), vascular dementia, very mild dementia (VMD), Dementia with Lewy Bodies and Mixed dementias. The type of dementia was reported in 27 studies and most participants were diagnosed with AD. Participant age ranged from 40-101 years with the majority over 75 years. Based on 36 studies, a majority of participants were female (M1046:F1452). The stage of cognitive impairment was cited in thirty-five studies as: Early (*n* = 16); Early/Moderate (*n* = 11); Mild/Moderate/Severe (*n* = 4); Advanced (*n* = 4).

Ethnicity was documented in 27 of the studies reviewed. Findings from these studies showed a mixture of White (*n* = 782); and non-white (*n* = 294) participants. The majority of non-white participants were linked to two studies (Jennings et al., 2016 [USA] and Jo et al., 2017 [Korea]).

#### Setting

The interventions were delivered in different settings including the participant’s home, community and clinical settings. Twenty-eight studies reported on the experience of home dwelling participants, nine on people living in residential care, and one in hospital.

#### Study intervention

Our investigation revealed that people living with dementia are willing to take part in numerous types of counselling and psychotherapeutic research interventions. These are listed in Table 3 below

**Table 3.** Types of intervention included in original research studies (*n* = 43) with first author.

Intervention type	First Authors
Cognitive behavioural therapy (CBT)	Baker, 2021; Blair, 2016; Garcia-Alberca, 2016; Jo, 2016; Tonga, 2016; Tonga, 2021; Spector, 2015; Staubo, 2017
Compassion focused therapy	Collins, 2017; Craig, 2018
Dignity therapy	Johnston, 2015; Johnston, 2017; Jenewein, 2021
Generic/Unspecified counselling modality within a care model	Cornelis, 2018; Hsiao, 2016; Jennings, 2016; Koivisto, 2016; Orsulic-Jeras, 2019; Skov, 2022; Whitlatch, 2019
Internal validation therapy	Erdmann, 2016
Marte Meo counelling	Berwig, 2020; Lykkeslet, 2016
Mentalisation	Luxmoore, 2017
Mindfulness based interventions	Churcher-Clarke, 2017; Douglas, 2021; Kovach, 2018; Paller, 2016
Pharmacy counselling	Alzubaidi, 2020; Plunger, 2020
Problem adaptation therapy (PATH)	Kiosses, 2015a; Kiosses, 2015b; McCombie, 2021
Psychotherapy	Bhattacharjee, 2017; Cheston, 2015; Cheston, 2017; Cheston, 2018; Marshall, 2015; Perren, 2018
QAR-Depression intervention	Bailey, 2017
Relational counselling (PCC)	Griffiths, 2020; Pybis, 2021
Talking therapy	Birtwell & Dubrow-Marshall, 2018

#### Timing, duration, attrition

The number of intervention sessions offered to participants varied across the different studies. Most were offered weekly over a 6–12 week time frame with an average of seven sessions per intervention, overall. Attrition was variably reported. Participation levels across the settings was difficult to compare and contrast due to the different circumstantial factors, for example being offered therapy at home or within a residential care context versus participants requiring to travel to a memory clinic or community venue.

#### Therapist

Intervention delivery was conducted by a range of therapists and clinicians including dementia care specialists, counsellors and psychotherapists, psychologists, pharmacists, nurses, social workers, and trainee psychologists.

#### Mode of Delivery

Fifteen studies had interventions delivered for people living with dementia individually, nine were focused on therapeutic support for couples and sixteen studies involved group interventions.

#### Adverse effects

In general, adverse effects were not reported. A handful of studies raised issues for future attention including: the negative impact of directive facilitation (Cheston, 2017); discriminatory attitudes and behaviour/therapeutic nihilism (Hsiao et al., 2016); non-reflective staff responding as a root cause of participant misunderstanding and emotional disengagement (Luxmoore & McEvoy, 2017); challenges to staff undertaking the interventions (Perren & Richardson, 2018); financial, relational, practical and attitudinal barriers; lack of staff training (Pybis et al., 2021) and, tensions between minimal resources, equitable access, and delivery of person-centred care (Plunger et al., 2020; Pybis et al., 2021).

#### Outcome measures

Across the different research studies the impact of the counselling/psychotherapeutic interventions on individuals with dementia, their care partners, and the therapeutic process was investigated through a wide-range of outcomes. In the quantitative studies (Tables 5 and 6) these were mainly linked to various aspects of cognitive function and diverse assessments of neuropsychiatric symptoms. The most frequently used quantitative outcome measures relating to people with dementia were, the Quality of Life–Alzheimer’s Disease (QOL-AD) questionnaire (Logsdon et al., 1999), used in fourteen studies and The Mini-Mental State Examination (MMSE) (Folstein et al., 1975) which recorded cognitive function in thirteen studies. The most frequently employed assessors of mood were: the Cornell Scale for Depression in Dementia [CSDD] (Alexopoulos et al., 1988) (*n* = 6); the General Depression Scale (GDS) (Yesavage, 1988), (*n* = 4); the Hospital Anxiety and Depression (HADS) (Zigmond and Snaith, 1983) (*n* = 5) and, the Rating Anxiety in Dementia (RAID) (Shankar et al., 1999) (*n* = 3). The remaining assortment of scales were used in one or two studies.

The qualitative studies (Table 4) investigated: participant response to a psychotherapeutic intervention; therapist and carer interaction and experience of counselling; the facilitation of appropriate communication; the mode of caring; identification of barriers and facilitators to a psychotherapeutic intervention for people living with dementia and potential adaptations. Eleven of these studies explored the topic from the people living with dementia’s viewpoint whilst four studies reported singularly through the eyes of health care professionals.

#### Countries represented

The majority of the original studies were conducted in Europe (*n* = 29), followed by the USA and Canada (*n* = 10), Asia (*n* = 2) Australia (*n* = 1) and the Middle East (*n* = 1). Over seventy per cent of the research was conducted in Europe, over fifty per cent of which was carried out in the UK.

## Review literature

Five of the eight reviews were conducted in England (*n* = 5) whilst the remaining three represented the USA, Malaysia and Sweden. Three included a Meta-Analyses (National Institute for Health and Care Excellence, 2018, Noone et al., 2019; Orgeta et al., 2015), three were Systematic Reviews (Cheston & Ivanecka, 2017; Shoesmith et al., 2020; Tay et al., 2019) and two, Literature Reviews (Bielsten and Hellström, 2019; Jao et al., 2017). Five of these eight studies were specific to anxiety and depression in people living with dementia (Cheston, 2017; NICE, 2018; Noone et al., 2019; Orgeta et al., 2015; Tay et al., 2019) whilst the more general effects of and/or process of psychotherapeutic interventions were studied in three reviews (Bielsten & Hellström, 2019; Jao et al., 2017; Shoesmith et al., 2020, 2022).

Sample sizes in the review literature were generally small but the collective findings suggest the benefits of counselling and psychotherapeutic interventions for people living with mild to moderate dementia and that these may be effective at improving depression and anxiety symptoms. The Orgeta et al. (2015) review, for example, found moderate quality evidence to show that psychological treatments can potentially, lead to improvements in both depressive and anxiety symptoms. No adverse events were reported.

Shoesmith et al., 2020 noted that most of the interventions adopted either a problem-solving or cognitive behavioural therapy approach with mixed results, overall. The authors recommended guidance for therapists and tailored interventions with key attention given to the relational context of participants and their caregiver(s). Bielsten and Hellström (2019) accented the lack of a genuine dyadic approach with greater attention given to carer’s emotional needs whilst an emphasis was placed on cognitive outcomes for the person living with dementia. Jao et al. (2017) found that support groups were well received and accepted by people living with dementia and their carers. Support groups with education did not, however, improve cognitive function for individuals with early stage dementia and their potential to result in negative affect was noted. A lack of ethnic diversity was also recorded. The NICE committee did not find any meaningful benefits from the trials of psychotherapy or on pre- and peri-diagnostic counselling and support for people living with dementia and their families (National Institute for Health and Care Excellence, 2018).

### Synthesis of original research study findings

The principal aim of this review was to explore the current knowledge on the role and impact of therapeutic counselling on the emotional health and well-being status of people living with dementia. The main themes identified across the original studies are summarised below:

#### Emotional and well-being benefits of therapeutic counselling

The research studies demonstrated a range of ways that people living with dementia can participate and gain emotional benefit from therapeutic counselling interventions. The combined evidence from across the different types of therapeutic interventions was mixed but overall, points to an improvement in depressive and anxiety symptoms in people living with early to moderate stage dementia. Improved mood and responding from those living with more advanced dementia was also reported. These benefits were mostly confined to the duration of the intervention period suggesting the value of here and now engagement. Interventions with a duration of 6–12 weeks were posited as feasible and acceptable to people living with dementia (and caregivers) living at home and in the residential care setting but the optimum length of sessions and their duration requires further investigation.

#### No one size fits all - relational and tailored approaches driven by person-centred values

Results from both the qualitative and quantitative studies did not show that any singular type of therapy worked better than another.

Across the different studies, an identified theme pointed to the need for therapists to employ a skilful, relational approach to support effective (and embodied) communication, placing value on enhancing the internal locus of personal control for the individual, regardless of the age, stage and form of dementia. Emphasis was placed on: person-centred values; relevant facilitative skills and the development of the therapeutic relationship; insight and sensitivity to life context and to cultural norms. Findings also indicated that therapies which employ a more structured approach such as CBT may require specific adaptations and carer involvement to realise beneficial outcomes. Tailored psychological support that aligns to personal preferences and individual abilities was emphasised.

The qualitative studies highlighted that participants experience a series of losses following a dementia diagnosis, distress that was intensified by the stigma of negative stereotyping which commonly led to low mood and diminished selfhood. A central part of the therapeutic process was, therefore, to support the reformulation of a person’s relationship to self while continuing to live with and work towards acceptance of the dementia.

#### Training, supervision and building community amongst counsellors

Professional perspectives on therapeutic counselling underlined the need for well-trained, deeply empathic practitioners who themselves receive frequent supervision, ongoing training and who are supported by positive encouragement and sufficient resources. Forging a sense of community amongst dementia counsellors and therapists was advocated to promote shared learning and ongoing practice improvement.

#### Involvement of people with dementia in therapeutic interventions

The small number of direct participants (excepting survey design studies), and the prevalence of pre-post data collection methods using different outcome measures impeded generalisability across the quantitative studies. Regardless, the potential value of counselling for people living with dementia was suggested. Important feasibility issues were, however noted. These were linked to practical and clinical issues including participant interest and readiness to take part. The findings invite questions about the capacity of people living with dementia to fully engage with a manualised programme and home practice and suggest the need for sufficient prior knowledge and understanding about a person’s wants, needs and aptitudes before taking part. Also featured were the lack of ethnic diversity in studies and racial/ethnic disparity in respect of depression treatment.

## Strengths and Limitations

We carried out a comprehensive, sensitive search to maximise the identification of eligible studies and minimise the risk of publication bias. The decision to feature studies with varied types of research design could be regarded as a drawback but including RCTs, qualitative research articles and other designs were included to provide strength and depth in relation to existing evidence on the topic. This followed the recommendation to expand review criteria from RCTs for non-pharmacological interventions in dementia to increase relevance and ecological legitimacy (Cohen-Mansfield et al., 2014). The miscellany of interventions and wide variety of outcome measures employed in the different studies prevented statistical comparability. Nevertheless, similar thematic features were noted across the different study designs.

Our research retrieved only articles written in the English-language which may have resulted in a failure to include relevant studies. Additionally, it is possible that we excluded some interventions that met the criteria for counselling and psychotherapy. In some instances, particularly when counselling was included as part of a multi-component programme, the interventions lacked a clear description. The distinction between what does or does not count as a therapeutic counselling intervention as referred to in the introduction is therefore, not always straightforward and we acknowledge a degree of ambiguity in this respect.

## Discussion

Our systematic review sought to uncover knowledge of the impact on emotional health and well-being status of people living with dementia following their participation in a therapeutic counselling intervention. The results point to the feasibility and potential benefits of counselling and psychotherapeutic interventions for people experiencing different stages of a dementia, including those with more advanced forms of dementia. The results from the review highlight, however, that people living with dementia are infrequent recipients of this form of support. Their recruitment into research studies exploring such interventions is modest overall with a general under-representation of minority ethnic groups. This remains the case from diagnosis to advanced stages of dementia and reflects an ongoing trend of omission as informants in research (Nygård, 2006). The need to involve people at different ages and stages of dementia in future counselling research intended to improve their emotional well-being is essential. This will help build and strengthen the relevance of the evidence base for therapeutic counselling. We acknowledge there are challenges around recruitment and retention but this review provides evidence that people living with dementia can participate in research, that there can be high compliance and that there are meaningful ways of capturing their experience, for example Marte Meo counselling which positively uses video to support reflection on the nuances of interaction between carers and people living with advanced dementia. 

In accord with earlier research (Luborsky et al., 2002), the different therapeutic approaches reviewed here appear to be mainly beneficial to participants. Outcomes of specific therapies are likely to change (and be of more or less relevance), depending on the stage and nature of a person’s dementia. The overriding factor, frequently cited in the qualitative studies and generally accepted within therapy, is the paramountcy of the therapeutic relationship (Paul and Charura, 2014). Bringing a relational understanding (Shoesmith et al., 2022) can help to contextualise the client’s experience, ensuring that the locus of control is firmly centred on their needs. A critical facet in treatment effectiveness, as Norcross and Lambert (2011) point out, is the need to adapt or tailor the therapeutic relationship to specific patient characteristics, taking any diagnoses into account. Without this, there is a risk that the person is fitted into the therapy rather than flexibly and creatively responding to presenting needs in the here and now.

The quantitative studies we reviewed mostly show a reduction in depression and anxiety in people with early to moderate dementia. In some of the cognitive-based therapy interventions we researched, the authors’ recommend frequent repetition to enhance the assimilation potential of people living with dementia to learn new information and skills. In these cases the need for caregiver participation to use a manualised programme and to engage with home practice was a key factor. The attainment of new information, development and measurement of cognitive skills may not, however, be a primary factor in supporting selfhood and emotional well-being. As a person living with dementia Bryden (2019, p. 75) espoused, ‘I am far more than a deteriorating self in an increasingly empty shell of a body, with disappearing neurones and neuronal pathways.’ Potential difficulties with the cognitive approach to therapy (and assessment) invites questions about its purpose in this context. Dewing (2002) alludes to the problem of a ‘hyper-cognitive’ culture that is at risk of neglecting the emotional, relational, aesthetic and spiritual abilities that remain present when memory and other cognitive capacities fade. Appropriately tailored therapy can help to detect and draw out existing qualities instead of trying to make better what has been lost. When researching dementia experience within the context of therapeutic interventions it would appear prudent to redress the imbalance caused by an over-emphasis on quantitative measurement of intellectual and perceptual faculties. We, therefore, question the emphasis placed by the NICE committee (National Institute for Health and Care Excellence, 2018) on findings arising from cognitive-function oriented trials and those which seek to measure and understand mood by questionnaire alone. While evaluating the outcomes of therapeutic counselling for people with dementia can be challenging due to impairments in communication, it is still possible to assess behavioural and functional improvements. By adopting a greater use of qualitative research and user-informed methods, careful attention can be given to the emotional, relational and spiritual dimensions of experience and improve understanding of what really matters to the people concerned.

The qualitative evidence suggests that counselling can strengthen emotional capacity and, that those living with the latter stages of this condition respond positively to a present moment, values-based, relational approach (Erdmann & Schnepp, 2016). Innovative methods that include embodied and sensory aspects in activities may offer greater relevance for people living with advanced dementia (Brown et al., 2020). Different counselling techniques can be customised to meet the individual’s changing needs while also satisfying the natural human desire to be connected and engaged (Brown et al., 2020). It is important to note that embodied and inter-embodied selfhood have previously been highlighted as principal factors in avoiding social death in relationships with people with advanced dementia (Watson, 2016).

Weight is often given to living well with dementia but over-emphasising this aspect may deny the reality of psychological distress that the condition can bring (Bartlett et al., 2017). Bartlett et al. emphasise the importance of realistic understanding of dementia impacts and that multiple perspectives are needed to discover and address the full range of experiences including emotional, physical, existential, and social pain. Support is needed to help people with dementia to find renewed meaning in a changing sense of self (Birtwell and Dubrow-Marshall, 2018). An accurate assessment of presenting needs and giving time to process experience is key. This requires attentive listening to allow those affected to explore and validate their emotional experience in a way that feels right for them. Professionally trained and adaptable communicators can help people to improve the quality of their close relationships and to shield against feelings of isolation and abandonment (Morgan, 2020). Both individual and family psychotherapy have been highlighted as valued approaches to help manage the emotional and behavioural symptoms that can occur as part of the dementia experience (Alzheimer's Scotland, 2016). It is important however, to ensure that carer needs to do not supersede those of the person with dementia and that autonomy is upheld. We discovered here that reported outcomes from the literature on therapeutic counselling interventions are frequently based on carer experience (Bielsten, 2019). Whilst we strongly advocate for carer therapeutic support in this context, it is imperative to stress that people living with mild, moderate and more advanced stages of a dementia can also express their views in verbal and non-verbal ways.

We recommend that therapists offering dementia counselling gain knowledge of the different dementias and their possible trajectories, so they can suitably adapt therapy to continually affirm and empower the client, regardless of the type and stage of dementia. Failure to do this can result in emotional disengagement and reinforce stigma and exclusion. Kitwood (1993)KITWOOD (1993) strongly emphasised the significance of responding to the person not the condition, yet the problematic portrayal of people living with dementia as a homogenous group remains a critical challenge. It depersonalises their needs and denies their human right to be regarded as individuals with unique personalities and distinctive needs (Gerritsen et al., 2016).

The rapidly evolving remit of pharmacists and their potential role in dementia counselling deserves attention. The two studies exploring pharmacy counselling in this context (Alzubaidi et al., 2020; Plunger et al., 2020) revealed key gaps in pharmacists’ communication skills and knowledge with only a small minority involved in the delivery of dementia counselling and/or structured person-centred care. The ‘Dementia-friendly Pharmacy’ programme (Plunger et al., 2020) offers a positive, user-informed model to address training needs and tackle the stigma that often accompanies a dementia diagnosis. The significance of dementia care is confirmed throughout the programme, leading to the launch of various initiatives. These include developing counselling skills, strengthening professional networks, and expanding medication-related knowledge. Consequently, pharmacy staff disclosed heightened self-rated competency in counselling people with dementia and their carers. These studies both highlight the importance of recognising diversity in terms of pharmacy structures, culture and local contexts. Therapeutic programmes developed in one setting may not, however, successfully translate to another and cultural and other adaptations may be necessary to make these suitable for different user groups (Cornelis et al., 2018). Beutler et al. (2004), suggest that ethnic matching in the therapeutic relationship may be linked to more positive outcomes although this area is not well researched.

Most of the original research studies reviewed here inclined towards time limited interventions without longer term follow up. Continuity and familiarity are, however, important for trust and confidence building. Although memory may be compromised, a felt sense of security through familiarity can build over time. In accord with Oyebode and Parveen (2019) we advocate the need for more studies that centre on supporting people living with dementia to remain within their own communities and which place emphasis on therapeutic interactions that help to promote the positive aspects of living with dementia.

In general, our findings suggest the beneficial effects of therapeutic counselling. Key recommendations arising from the review have been listed in Table 9. The breadth and scope of our approach provides a broader insight than that produced from systematic reviews of randomised trial evidence alone. Although the nature and strength of the evidence is varied, results from this research indicate that therapeutic interventions are important to support the emotional impact of living with dementia. These appear to carry low risks and minimal adverse effects. Further, therapeutic counselling that is sensitively shaped to individual needs holds the potential for widespread accessibility.

## Conclusion

In this systematic review, we examined the role and impact of counselling and psychotherapeutic interventions on the emotional experience of people living with dementia. Our findings show that different therapeutic approaches have been tested with people at different stages of a dementia diagnosis. The results suggest the value of counselling as a supportive medium to help with the processing and coping of difficult emotions and feelings across the trajectory of a dementia. Currently, there is limited psychological support available to help people living with dementia to deal with the emotional consequences of their condition. We hope that this review will help to increase awareness and recognition of this important area of unmet need and stimulate further research on the topic.

## Supplemental Material

Supplemental Material - The role and impact of therapeutic counselling on the emotional experience of adults living with dementia: A systematic review Supplemental Material for The role and impact of therapeutic counselling on the emotional experience of adults living with dementia: A systematic review by Gill Mathews, Xiaoyang Li, and Heather Wilkinson in Dementia: the international journal of social research and practice

Supplemental Material - The role and impact of therapeutic counselling on the emotional experience of adults living with dementia: A systematic review Supplemental Material for The role and impact of therapeutic counselling on the emotional experience of adults living with dementia: A systematic review by Gill Mathews, Xiaoyang Li, and Heather Wilkinson in Dementia: the international journal of social research and practice

Supplemental Material - The role and impact of therapeutic counselling on the emotional experience of adults living with dementia: A systematic review Supplemental Material for The role and impact of therapeutic counselling on the emotional experience of adults living with dementia: A systematic review by Gill Mathews, Xiaoyang Li, and Heather Wilkinson in Dementia: the international journal of social research and practice

Supplemental Material - The role and impact of therapeutic counselling on the emotional experience of adults living with dementia: A systematic review Supplemental Material for The role and impact of therapeutic counselling on the emotional experience of adults living with dementia: A systematic review by Gill Mathews, Xiaoyang Li, and Heather Wilkinson in Dementia: the international journal of social research and practice

Supplemental Material - The role and impact of therapeutic counselling on the emotional experience of adults living with dementia: A systematic review Supplemental Material for The role and impact of therapeutic counselling on the emotional experience of adults living with dementia: A systematic review by Gill Mathews, Xiaoyang Li, and Heather Wilkinson in Dementia: the international journal of social research and practice

Supplemental Material - The role and impact of therapeutic counselling on the emotional experience of adults living with dementia: A systematic review Supplemental Material for The role and impact of therapeutic counselling on the emotional experience of adults living with dementia: A systematic review by Gill Mathews, Xiaoyang Li, and Heather Wilkinson in Dementia: the international journal of social research and practice

Supplemental Material - The role and impact of therapeutic counselling on the emotional experience of adults living with dementia: A systematic review Supplemental Material for The role and impact of therapeutic counselling on the emotional experience of adults living with dementia: A systematic review by Gill Mathews, Xiaoyang Li, and Heather Wilkinson in Dementia: the international journal of social research and practice

Supplemental Material - The role and impact of therapeutic counselling on the emotional experience of adults living with dementia: A systematic review Supplemental Material for The role and impact of therapeutic counselling on the emotional experience of adults living with dementia: A systematic review by Gill Mathews, Xiaoyang Li, and Heather Wilkinson in Dementia: the international journal of social research and practice

Supplemental Material - The role and impact of therapeutic counselling on the emotional experience of adults living with dementia: A systematic review Supplemental Material for The role and impact of therapeutic counselling on the emotional experience of adults living with dementia: A systematic review by Gill Mathews, Xiaoyang Li, and Heather Wilkinson in Dementia: the international journal of social research and practice
