# Supplementary material for: The role and impact of therapeutic counselling on the emotional experience of adults living with dementia: A systematic review
Source: Dementia (London). 2024 Apr 16;23(5):882–902. doi: 10.1177/14713012241233765 (PMC11163847; doi:10.1177/14713012241233765)
Supplement: Supplemental Material - The role and impact of therapeutic counselling on the emotional experience of adults living with dementia: A systematic review [file sj-pdf-1-dem-10.1177_14713012241233765.pdf]

Supplementary Files: The role and impact of therapeutic counselling on the emotional experience of adults living with dementia: a systematic review

Title and description of supplementary files in the order that these appear within the main article:-

Title: Table 1. Databases Searched

Description: List of databases included in the electronic search for relevant articles

Title: Appendix 1**.** MEDLINE detailed search terms

Description: Detailed search terms used to build the search for relevant articles on the main academic databases

Title: Fig. 1. Counselling People with Dementia: PRISMA flow chart (Moher et al., 2009)

Description: Documentation of the article screening process

Title: Appendix 2. Counselling People with Dementia – Excluded Articles

Description: Tabulated list of articles excluded from the review giving brief information on the content and reason for omission

Title: Table 2: Inclusion and exclusion criteria

Description: Tabulated account of the inclusion and exclusion criteria for studies following the PICO format.

Title: Table 3: CASP Scoring Tables

Description: The Critical Appraisal Skills Programme (CASP) tools were used to consider methodological quality. These tools provide a recognised method for assessing evidence for use in healthcare practice and policy development. A scoring system was applied to further assist in the process of assessing studies included in the review. The 10 CASP criteria were scored as follows: a score of 0 meaning not or inadequately addressed, a score of 1 meaning the criterion was partially addressed and a score of 2 meaning the criterion was fully addressed. A total score between 15 and 20 was considered to be ‘high’ quality, ‘moderate’ papers scored between 8 and 14 and ‘low’ quality papers scored between 0 and 7. Papers were assessed by two reviewers independently, who then agreed the final rating. Although no papers were discounted on grounds of quality, the quality assessment enabled a more robust review of the studies.

Title: Table 4: Characteristics Qualitative Studies

Description: Tabulated details of the qualitative articles included in the review

Title: Table 5: Characteristics Randomised Controlled Trials

Description: Tabulated details of the randomised controlled trial articles included in the review

Title: Table 6: Characteristics Other Study Designs

Description: Tabulated details of the ‘other study design’ articles included in the review

Title: Table 7: Characteristics Systematic Reviews

Description: Tabulated details of the systematic review articles included in the review

Title: Table 8: Types of intervention included in original research studies

Description: List of counselling/psychotherapeutic interventions, e.g. Cognitive Behavioural Therapy (CBT)**,** Compassion Focused Therapy along with the first author of the research article in which these were described.

Title: Table 9: List of Recommendations

Description: List of recommendations arising from the review process
