# Supplementary material for: The role and impact of therapeutic counselling on the emotional experience of adults living with dementia: A systematic review
Source: Dementia (London). 2024 Apr 16;23(5):882–902. doi: 10.1177/14713012241233765 (PMC11163847; doi:10.1177/14713012241233765)
Supplement: Supplemental Material - The role and impact of therapeutic counselling on the emotional experience of adults living with dementia: A systematic review [file sj-pdf-2-dem-10.1177_14713012241233765.pdf]

MEDLINE detailed search terms

1.	(People or participants or adults or humans).mp. [mp=title, abstract, original title, name of substance word, subject heading word, floating sub-heading word, keyword heading word, organism supplementary concept word, protocol supplementary concept word, rare disease supplementary concept word, unique identifier, synonyms]
2.	exp Child/
3.	1 not 2
4.	(animals not humans).mp. [mp=title, abstract, original title, name of substance word, subject heading word, floating sub-heading word, keyword heading word, organism supplementary concept word, protocol supplementary concept word, rare disease supplementary concept word, unique identifier, synonyms]
5.	3 not 4
6.	(Dementia or Alzheimer* disease or vascular dementia or Lewy-body dementia or dementia with Lewy bodies or DLB or diagnosis of dementia).mp. [mp=title, abstract, original title, name of substance word, subject heading word, floating sub-heading word, keyword heading word, organism supplementary concept word, protocol supplementary concept word, rare disease supplementary concept word, unique identifier, synonyms]
7.	(Counselling or counseling or individual counselling or dyad* counselling or group counselling or psychotherapy or individual psychotherapy or dyad* psychotherapy or group psychotherapy).mp. [mp=title, abstract, original title, name of substance word, subject heading word, floating sub-heading word, keyword heading word, organism supplementary concept word, protocol supplementary concept word, rare disease supplementary concept word, unique identifier, synonyms]
8.	(Humanistic counselling or humanistic therapy or non-directive counselling or non-directive therapy or client-centred therapy or client centred therapy or humanistic-experiential psychotherapy or HEP or Person Centred Counselling or Person-Centred Counselling or PCC or Person Centred Therapy or PCT or Rogerian or Gestalt or Transactional Analysis or TA or Emotion-Focused Therapy or Emotion Focused Therapy or EFT or Psychoanalytic therapy or PAT or Psychodynamic therapy or PDT or Psychodynamic counselling or process-experiential therapy or supportive-expressive or Cognitive-Behavioural counselling or Cognitive Behavioural Therapy or CBT or Rational Emotive Counselling or RET or Integrative counselling or pluralistic counselling or integrative therapy or pluralistic therapy or pastoral counselling).mp. [mp=title, abstract, original title, name of substance word, subject heading word, floating sub-heading word, keyword heading word, organism supplementary concept word, protocol supplementary concept word, rare disease supplementary concept word, unique identifier, synonyms]
9.	(Mindfulness Based Interventions or Mindfulness-Based Interventions or MBIs or Mindfulness-Based Stress Reduction or Mindfulness Based Stress Reduction or MBSR or Mindfulness-Based Cognitive Therapy or Mindfulness Based Cognitive Therapy or MBCT).mp. [mp=title, abstract, original title, name of substance word, subject heading word, floating sub-heading word, keyword heading word, organism supplementary concept word, protocol supplementary concept word, rare disease supplementary concept word, unique identifier, synonyms]
10.	7 or 8 or 9
11.	exp Randomized Controlled Trial/

12.	exp Controlled Clinical Trial/
13.	(RCT or randomized or randomised or randomly or random allocation).mp. [mp=title, abstract, original title, name of substance word, subject heading word, floating sub-heading word, keyword heading word, organism supplementary concept word, protocol supplementary concept word, rare disease supplementary concept word, unique identifier, synonyms]
14.	(Clinical Trials or trial or Randomized Controlled Trials).mp. [mp=title, abstract, original title, name of substance word, subject heading word, floating sub-heading word, keyword heading word, organism supplementary concept word, protocol supplementary concept word, rare disease supplementary concept word, unique identifier, synonyms]
15.	(((group*1 or control*1) and treatment as usual) or TAU or usual care or care as usual or waitlist* or wait*list).mp. [mp=title, abstract, original title, name of substance word, subject heading word, floating sub-heading word, keyword heading word, organism supplementary concept word, protocol supplementary concept word, rare disease supplementary concept word, unique identifier, synonyms]
16.	11 or 12 or 13 or 14 or 15
17.	(observational study or case-control or cohort).mp. [mp=title, abstract, original title, name of substance word, subject heading word, floating sub-heading word, keyword heading word, organism supplementary concept word, protocol supplementary concept word, rare disease supplementary concept word, unique identifier, synonyms]
18.	exp Qualitative Research/
19.	16 or 17 or 18
20.	5 and 6 and 10 and 16 and 19
