# Supplementary material for: The role and impact of therapeutic counselling on the emotional experience of adults living with dementia: A systematic review
Source: Dementia (London). 2024 Apr 16;23(5):882–902. doi: 10.1177/14713012241233765 (PMC11163847; doi:10.1177/14713012241233765)
Supplement: Supplemental Material - The role and impact of therapeutic counselling on the emotional experience of adults living with dementia: A systematic review [file sj-pdf-3-dem-10.1177_14713012241233765.pdf]

Counselling People with Dementia – Excluded Articles

	First Author	Date	Title	Type	Reason for Exclusion
1.	Aftab, A	2019	Well-being in dementia and mild cognitive impairment	Editorial	Wrong Intervention<br>No specific reference to counselling/psychotherapy
2.	Aguirre, A.	2019	Combining Value-Based And Collaborative Care Models In Dementia Care	Conference Abstract	Wrong design
3.	Ahmed, S.	2016	Frontotemporal Dementia treatment: a review	Review	Wrong design Short review with no detail other than a recommendation for counselling or psychotherapy in FTD
4.	Aldridge, Z	2019	ABC model: A tiered, integrated pathway approach to peri- and post-diagnostic support for families living with dementia (Innovative Practice)	Mixed Methods	Wrong Intervention<br>Admiral Nurse Model – focus on case management with no specific reference to counselling/ psychotherapy
5.	Alzheimer's Society	2015	Talking therapies	Factsheet	Wrong design
6.	Anderson, J.	2017	Non-pharmacological Strategies for Patients With Early-Stage Dementia or Mild Cognitive Impairment: A 10-Year Update	Review	Wrong Intervention<br>No specific reference to counselling/psychotherapy. Reference to MBI's but all outwith the date range.
7.	Andrews, L.	2016	Treating depression and anxiety in people with dementia	Brief evidence	Wrong population<br>Commentary on Orgeta V et al (2014) Psychological treatments for depression and anxiety in dementia and mild cognitive impairment. Cochrane Database of Systematic Reviews 2014, 1: CD009125.
8.	Auer, S.	2015	Dementia service centres in Austria	Model description	Wrong design
9.	Backhouse, A.	2017	The effectiveness of community-based coordinating interventions in dementia care: a meta-analysis and subgroup analysis of intervention components	Meta-analysis	Wrong Intervention<br>No specific reference to counselling/psychotherapy

Counselling People with Dementia – Excluded Articles					
	**First Author**	**Date**	**Title**	**Type**	**Reason for Exclusion**
**10.**	Ballard, C.	2018	Impact of person-centred care training and person-centred activities on quality of life, agitation, and antipsychotic use in people with dementia living in nursing homes: A cluster randomised controlled trial	RCT	Wrong Intervention<br>WHELD Trial – Person-centred approach but no specific reference to counselling/psychotherapy
**11.**	Bartlett, R.	2017	Suffering with dementia: the other side of "living well''	Position Paper	Wrong Intervention<br>No specific reference to counselling/psychotherapy
**12.**	Bartlett, R.	2018	Life at Home for People with a Dementia	Book	Wrong Design
**13.**	Bass, D.	2015	Impact of the care coordination program "partners in Dementia Care" on veterans' hospital admissions and emergency department visits	RCT	Wrong Population<br>Carer support
**14.**	Bass, D.	2015	Reflections on Implementing the Evidence-Based BRI Care Consultation with RCI in Georgia	Position Paper<br>Implementation Science<br>Model of Care	Wrong Population<br>Care model focusing on home carer support<br>Commentary on implementation of evidence-based dementia care programme
**15.**	Berk, L.	2018	Mindfulness Training for People With Dementia and Their Caregivers: Rationale, Current Research, and Future Directions.	Review	Relates to participants with Mild Cognitive Impairment (MCI) or Subjective Cognitive Decline (SCD) – not diagnosed with dementia
**16.**	Bjorge, H.	2019	The effect of psychosocial support on caregivers' perceived criticism and emotional over-involvement of persons with dementia: an assessor-blinded randomized controlled trial	Blinded RCT	Wrong Population<br>Carer perspective

Counselling People with Dementia – Excluded Articles					
	**First Author**	**Date**	**Title**	**Type**	**Reason for Exclusion**
**17.**	Bohlken, J.	2020	Needs of Patients with Mild Cognitive Disorders in a Specialist Practice.	Methods Survey of 51 PmMCI in a specialist care practice	Wrong Population<br>MCI not dementia
**18.**	Borson, S.	2019	Reducing Barriers to Mental Health Care: Bringing Evidence-Based Psychotherapy Home.	Implementation Science	Wrong Population<br>Focus on older adults and caregivers – not specific to PwD
**19.**	Bosco, A.	2019	Promoting personhood through co-production.	Review	Wrong Intervention<br>Coproduction - personhood and dignity in dementia care
**20.**	Botte, A.	2019	Reaching the unreachable: a community-based dementia educator	Position paper Care Model	Wrong Intervention
**21.**	Botte, A.	2018	Eye movement desensitization and reprocessing	Position Paper	Wrong Design<br>Clinical Evidence
**22.**	Botte, A.	2017	Improving the quality of mental health care offered to dementia patients and their families through co-located medical and mental health services	Position Paper	Wrong Design
**23.**	Brando, E. B.	2017	The application of technologies in dementia diagnosis and intervention:	literature review.	Wrong Intervention<br>No reference to counselling/psychotherapy for PwD
**24.**	Braun, A.	2018	Managing behavioural and psychological symptoms in community dwelling older people with dementia .	A systematic review of qualitative studies	Wrong Intervention<br>No reference to counselling/psychotherapy for PwD

Counselling People with Dementia – Excluded Articles					
	**First Author**	**Date**	**Title**	**Type**	**Reason for Exclusion**
**25.**	Bryden, C.	2019	Challenging the discourses of loss: A continuing sense of self within the lived experience of dementia.	Autoethnography	Wrong Intervention but very useful insights into personal experience of living with dementia and importance of recognising present moment selfhood
**26.**	Carrion, C.	2018	Cognitive Therapy for Dementia Patients	A Systematic Review	Wrong Intervention<br>No reference to counselling/psychotherapy<br>Reality orientation therapy and cognitive skills training –focus on improving cognition not emotional well-being
**27.**	Carter, M.	2019	An individualised, non-pharmacological treatment strategy associated with an improvement in neuropsychiatric symptoms in a man with dementia living at home.	Autoethnography	Mentions use of validation and talking therapy in single sentences – no detail on how implemented.
**28.**	Carter, M.	2019	A Structured Cognitive Intervention Pathway as a decision-support tool for non-pharmacological interventions within a dementia care service	Care model	Wrong Intervention<br>No reference to counselling/psychotherapy for PwD
**29.**	Casey, D.	2019	Telling a 'good or white lie': The views of people living with dementia and their carers.	Qualitative study	Wrong Intervention<br>No reference to counselling/psychotherapy for PwD
**30.**	Cassidy-Eagle, E.	2018	Neuropsychological Functioning in Older Adults with Mild Cognitive Impairment and Insomnia Randomized to CBT-I or Control	RCT	Wrong population<br>MCI not dementia

Counselling People with Dementia – Excluded Articles					
	**First Author**	**Date**	**Title**	**Type**	**Reason for Exclusion**
			Group.		

Counselling People with Dementia – Excluded Articles					
	**First Author**	**Date**	**Title**	**Type**	**Reason for Exclusion**
**31.**	Chan, J.	2015	A Mindfulness Programme for People with Dementia in Care Homes	Systematic Review RCT Pilot	Wrong Intervention
**32.**	Charlesworth, G.	2015	Cognitive Behaviour Therapy for Anxiety in People With Dementia	Modality Description	Wrong Design
**33.**	Chatwin, J.	2017	The influence of subliminal crosstalk in dementia narratives. Dementia, 18(5), 1740-1750. doi:10.1177/1471301217724922	Ethnographic Study	Wrong Intervention<br>No reference to counselling/psychotherapy for PwD – research on interaction/communication in Care Home setting
**34.**	Chiu, H. Y.	2018	Reality orientation therapy benefits cognition in older people with dementia: A meta-analysis. Int J Nurs Stud, 86, 20-28. doi:10.1016/j.ijnurstu.2018.06.008	Meta-analysis.	Wrong Intervention<br>No specific reference to counselling/psychotherapy
**35.**	Clare, L.	2019	Individual goal-oriented cognitive rehabilitation to improve everyday functioning for people with early-stage dementia: A multicentre randomised controlled trial (the GREAT trial.	RCT	Wrong Intervention<br>No specific reference to counselling/psychotherapy
**36.**	Clare, L.	2019	Cognitive rehabilitation, self-management, psychotherapeutic and caregiver support interventions in progressive neurodegenerative conditions:A scoping review	Review<br>(Cochrane)	Wrong population/intervention<br>Mainly carer support<br>out of date range
**37.**	Clarkson, P.	2018	Systematic review: Effective home support in dementia care,	Review	Wrong Intervention<br>Only one reference to family

Counselling People with Dementia – Excluded Articles					
	**First Author**	**Date**	**Title**	**Type**	**Reason for Exclusion**
			components and impacts - Stage 2, effectiveness of home support interventions		counselling/psychotherapy<br>out of date range

Counselling People with Dementia – Excluded Articles					
	**First Author**	**Date**	**Title**	**Type**	**Reason for Exclusion**
**38.**	Clift, K.	2016	Familial Creutzfeldt-Jakob Disease: Case report and role of genetic counseling in post mortem testing	Case Report	Wrong Population
**39.**	Connors, M. H.	2018	Non-pharmacological interventions for Lewy body dementia	Systematic review	Wrong Intervention
**40.**	Crook, A.	2017	Predictive genetic testing for amyotrophic lateral sclerosis and frontotemporal dementia: genetic counselling considerations	Original Research	Protocol for systematic review
**41.**	Culler, S. L.	2018	Importance of structured biopsychosocial assessment (bpsa) for people with dementia (pwd) and their care partners	Position Paper	Wrong Design
**42.**	de Witt, L., & Ploeg, J.	2014	Caring for older people living alone with dementia: Healthcare professionals' experiences.	Qualitative research - interviews	Wrong Intervention but useful reference material
**43.**	DeMarco, M.	2017	On (Writing) Families: Autoethnographies of Presence and Absence, Love and Loss, by Jonathan Wyatt and Tony E. Adams, eds, Netherlands, Sense Publishers,	Book Review	No reference to PwD in review
**44.**	Davison, T.	2016	Brief on the Role of Psychologists in Residential and Home Care Services for Older Adults	Narrative Review	Wrong Design

Counselling People with Dementia – Excluded Articles					
	**First Author**	**Date**	**Title**	**Type**	**Reason for Exclusion**
**45.**	Dening, K. H.	2018	Palliative care in dementia: a fragmented pathway?	Position Paper	Wrong Design
**46.**	Dewey, A.	2016	The role of psychological treatments for depression and anxiety in dementia	Position Paper	Wrong Design
**47.**					
**48.**					
**49.**	Dham, P. (2019).	2019	Collaborative care initiative for mental health risk factors in dementia: Depression, anxiety and mild cognitive impairment.	Systematic Review	One study linked to collaborative care in dementia but out of date
**50.**	Dimitriou, T. D.	2018	Non-pharmacological interventions for agitation/aggressive behaviour in patients with dementia	RCT	Wrong Intervention<br>No specific reference to counselling/psychotherapy
**51.**	Dodd, K.	2018	Consensus statement of the international summit on intellectual disability and Dementia related to post-diagnostic support	Consensus statement	Wrong population<br>No specific reference to counselling/ psychotherapy
**52.**	Dooley, J.	2015	Communication in healthcare interactions in dementia: a systematic review of observational studies.	Systematic review	Wrong intervention
**53.**	Drayton, S.	2017	Achieving positive outcomes in complex cases: The Admiral Nurse Dementia Helpline	Innovative practice model	Wrong population – caregiver support
**54.**	Dugmore, M.	2015	Qualitative studies of psychosocial	systematic	Wrong intervention No specific reference to counselling/

Counselling People with Dementia – Excluded Articles					
	**First Author**	**Date**	**Title**	**Type**	**Reason for Exclusion**
			interventions for dementia	review	psychotherapy

Counselling People with Dementia – Excluded Articles					
	**First Author**	**Date**	**Title**	**Type**	**Reason for Exclusion**
**55.**	Dunham, A.	2019	Impact of disclosure of a dementia diagnosis on uptake of support services: A pilot study exploring a post-traumatic stress approach	Pilot study	Wrong Intervention<br>Not specific to counselling/psychotherapy but useful info for discussion
**56.**	Dyer, S. M.	2018	An overview of systematic reviews of pharmacological and non-pharmacological interventions for the treatment of behavioral and psychological symptoms of dementia.	overview of systematic reviews	Wrong Intervention<br>Not specific to counselling/psychotherapy
**57.**	Dyakova, M.	2017	Investment for health and well-being: a review of the social return on investment from public health policies to support implementing the Sustainable Development Goals by building on Health 2020.	WHO Report	Wrong population<br>No specific reference to counselling/psychotherapy for PwD
**58.**	Eriksen, S.	2018	The experience of lived space in persons with dementia: a systematic meta-synthesis	Review systematic meta-synthesis	Not specific to counselling/psychotherapy but review includes studies that invite the user's psychological experience and highlight needs
**59.**	Esandi, N.	2016	Family-centered care: A model for approaching dementia care in the community.	Model of care description	No specific reference to counselling/psychotherapy for PwD<br>Written in Spanish
**60.**	Eguchi, D.	2019	The Effectiveness of Problem Adaptation Therapy (Path) in a Culture and Language Rich Community	Retrospective review of participant records	No full text – conference Poster.<br>Unclear whether participants had dementia
**61.**	Eichler, T.	2019	Unmet Needs of Community-Dwelling Primary Care Patients with Dementia in	RCT – Baseline data from GP-	Wrong Intervention but interesting

Counselling People with Dementia – Excluded Articles					
	**First Author**	**Date**	**Title**	**Type**	**Reason for Exclusion**
			Germany: Prevalence and Correlates	based, cluster-randomized controlled intervention trial	

Counselling People with Dementia – Excluded Articles					
	**First Author**	**Date**	**Title**	**Type**	**Reason for Exclusion**
**62.**	Fang, X.	2017	Prevention of dementia and care of dementia patients from nursing perspective: best practices and development needs.	Thesis Lit review	Wrong Intervention<br>Not specific to counselling/psychotherapy
**63.**	Farhang, M.	2019	Impact of mind-body interventions in older adults with mild cognitive impairment: a systematic review	Systematic Review	Wrong population
**64.**	Farrand, P.	2016	Psychological interventions to improve psychological well-being in people with dementia or mild cognitive impairment	Systematic review and meta-analysis protocol.	Protocol – e-mail sent to first author re study status 12/05/20 study completed but not yet published
**65.**	Ford, A.	2015	Psychological treatment for depression and anxiety associated with dementia and mild cognitive impairment	Clinical evidence - Editorial Commentary	A systematic review of psychological treatments in this issue (Ortega et al 2015) highlights the current paucity of good-quality data, but suggests these interventions hold promise. novel adequately powered randomised controlled trials needed.
**66.**	Frank, A.	2017	A patient's experience in dementia care Using the "lived experience" to improve care	Case study/literature search	Wrong Design<br>Framework for dementia care
**67.**	Fredriksen-Goldsen, K.	2016	Cognitive Impairment, Alzheimer's Disease, and Other Dementias in the Lives of Lesbian, Gay, Bisexual and Transgender (LGBT) Older Adults and Their Caregivers: Needs and Competencies.	Grey -Position Paper outlining competencies for LGBT framework based on prior Systematic Review	Need for welcoming and affirming environments through targeted outreach, recognizing community needs, and building capacity to advocate and develop needed services. Evidence-based trainings and innovative service models are needed to increase the knowledge and skills of providers

Counselling People with Dementia – Excluded Articles					
	**First Author**	**Date**	**Title**	**Type**	**Reason for Exclusion**
**68.**	Freeman, M.	2016	From absence to presence. Finding Mother, Ever Again	Book Chapter Autoethnograph y	In On (Writing) Families: Autoethnographies of Presence and Absence – insight into mother-son relationship/trajectory of dementia
**69.**	Gajardo, J.	2017	The Kintun program for families with dementia: From novel experiment to national policy (innovative practice).	Care model	Description of evolving dementia in Chile
**70.**	Grünzig, M.	2020	Die häusliche Situation eines Ehepaares Challenges in dementia care at home - The situation at home of a married couple.	Case Management Study	Wrong Intervention<br>Not specific to counselling/psychotherapy
**71.**	Guan, Y.	2018	The impact of genetic counselors' use of facilitative strategies on cognitive and emotional processing of genetic risk disclosure for Alzheimer's disease. Highlights mechanisms used by genetic counsellors to support understanding of risk information	Analysis of sub-set of data (audio and transcripts) from multi-site RCT (READ)	Wrong population (MCI)
**72.**	Guideline Adaptation Committee	2016	Clinical Practice Guidelines And Principles Of Care For People With Dementia	Clinical Guideline	Wrong Design
**73.**	Gustavson, K.	2016	Problem-Solving Therapy Reduces Suicidal Ideation In Depressed Older Adults with Executive Dysfunction	Sub-set of RCT	Wrong population
**74.**	Hadar, B.	2018	Response to 'Everybody needs a group: A qualitative study looking at therapists' views of the role of	Commentary on study	Full text of the relevant study (Perren et al.) already sourced

Counselling People with Dementia – Excluded Articles					
	**First Author**	**Date**	**Title**	**Type**	**Reason for Exclusion**
			psychotherapy groups in working with older people with dementia and complex needs'.		

Counselling People with Dementia – Excluded Articles					
	**First Author**	**Date**	**Title**	**Type**	**Reason for Exclusion**
**75.**	Harwood, R.	2018	A staff training intervention to improve communication between people living with dementia and health-care professionals in hospital: the VOICE mixed-methods development and evaluation study.	Report on original research	Wrong intervention but useful information on communication techniques and staff training
**76.**	Hazlett-Stevens, H.	2019	Mindfulness-Based Stress Reduction and Mindfulness-Based Cognitive Therapy with Older Adults	Qualitative Review of RCT Research.	Wrong population
**77.**	Helmes, E., & Ward, B. G.	2017	Mindfulness-based cognitive therapy for anxiety symptoms in older adults in residential care	RCT	Wrong population
**78.**	Hernandez, E	2017	"We are a Team": Couple Identity and Memory Loss.	Qualitative IPA	Wrong intervention<br>Couple identity<br>Couples Life Story Project, a life review intervention where one partner has memory loss
**79.**	Hochgraeber, I.	2015	Low-threshold support services for people with dementia within the scope of respite care in Germany – A qualitative study on different stakeholders' perspective	Qualitative	Wrong intervention<br>useful information on German modes of working in dementia care
**80.**	Holopainen, A.	2017	Factors Associated with the Quality of Life of People with Dementia and with Quality of Life-Improving Interventions: Scoping Review.	Systematic Review	Wrong Intervention<br>Not specific to counselling/psychotherapy but brief ref to counselling -Guidance, counselling and social support would seem to be important, especially in the early stages of dementia (Leung, Orrell, & Orgeta, 2015), when both the patient and his or her next of kin have a great need for information.

Counselling People with Dementia – Excluded Articles					
	**First Author**	**Date**	**Title**	**Type**	**Reason for Exclusion**
**81.**	Hughes, J. C. E.	2018	Ethical Issues in Older Patients.	Book chapter	Wrong Intervention<br>Not specific to counselling/psychotherapy
**82.**	Huis in het Veld, J. G.,	2018	A systematic meta-review of self-management support for people with dementia.	Systematic Meta-Review	Wrong Intervention
**83.**	Hutchinson, K.	2018	Co-creation of a family-focused service model living with younger onset dementia.	Qualitative	Wrong Intervention<br>Not specific to counselling/psychotherapy
**84.**	Iek Long, L.	2019	Macao Dementia Policy: Challenges and prospects (innovative practice).	Regional Action Plan	Chinese strategic action plan scant reference to counselling except for post diagnostic referral
**85.**	Iliffe, S.	2017	Case Management for People with Dementia and its Translations: A Discussion Paper.	Position Paper	Wrong Intervention<br>Not specific to counselling/psychotherapy
**86.**	Ingersoll-Dayton, B.	2014	Creating a duet: The Couples Life Story Approach in the United States and Japan		Wrong Intervention<br>Out of date range
**87.**	Institute for Quality and Efficiency in Health Care	2017	Systemic therapy in adults as a psychotherapeutic approach. Retrieved from Kln, Germany:	Brief extract from Report on Systematic Review	1 study linked to dementia – no benefit for ST - outwith date range Full report only accessible in German language
**88.**	Jacqui	2020	Jacqui is struggling with side-effects of new medication, depression and lack of support	Dementia Diary	Not a research article
**89.**	Jackson, D.	2016	A systematic review of the effect of telephone, internet or combined support for carers of people living with Alzheimer's, vascular or mixed dementia in the community	Systematic Review	Wrong population - caregivers
**90.**	Jeong, J. H.	2016	Group- and Home-Based Cognitive	RCT	Wrong population - MCI

Counselling People with Dementia – Excluded Articles					
	**First Author**	**Date**	**Title**	**Type**	**Reason for Exclusion**
			Intervention for Patients with Mild Cognitive Impairment: A Randomized Controlled Trial.		

Counselling People with Dementia – Excluded Articles					
	**First Author**	**Date**	**Title**	**Type**	**Reason for Exclusion**
**91.**	Johannessen, A.	2015	Family carers' experiences of attending a multicomponent psychosocial intervention program for carers and persons with dementia	Qualitative interviews	Wrong population – caregivers but useful study for discussion
**92.**	Johnston, L.	2015	Dynamics in Couples Facing Early Alzheimer's Disease	Qualitative Interviews	Understanding the experiences of couples in the early stages of AD can help the therapist develop greater empathy and a willingness to work with this population. If a model can be developed which includes the experiences of couples who successfully cope with AD, therapists could use this model to help other couples move through the process of maintaining a relationship
**93.**	Jokel, R.	2017	Group intervention for individuals with primary progressive aphasia and their spouses: Who comes first?	Pre-post comparison-group pilot	Wrong population but useful study for discussion Stresses importance of dyadic intervention and needs of both parties have to be addressed simultaneously. Value of educational input and emotional support
**94.**	Jutkowitz, E.	2016	Care-Delivery Interventions to Manage Agitation and Aggression in Dementia Nursing Home and Assisted Living Residents	Systematic Review and Meta-analysis	Wrong Intervention<br>Not counselling/ psychotherapy
**95.**	Kales, H.	2015	Assessment and management of behavioral and psychological symptoms of dementia.	Review	Wrong Intervention<br>No counselling/ psychotherapy
**96.**	Karel, M. J.	2016	Effectiveness of Expanded Implementation of STAR-VA for Managing Dementia-Related Behaviors	Care model	Wrong Intervention<br>No counselling/ psychotherapy

Counselling People with Dementia – Excluded Articles					
	**First Author**	**Date**	**Title**	**Type**	**Reason for Exclusion**
			Among Veterans.		

Counselling People with Dementia – Excluded Articles					
	**First Author**	**Date**	**Title**	**Type**	**Reason for Exclusion**
**97.**	Kaufmann, E. G.	2014	Dementia and well-being: A conceptual framework based on Tom Kitwood's model of needs.	Qualitative research with PwD	No counselling/ psychotherapy<br>But interesting study on emotional needs of PwD
**98.**	Kessler, E.-M.	2018	Psychotherapeutic work with old and very old people	Review	In German – Interesting to know content -?<br>Focus on older people or/and PwD
**99.**	Khalsa, D. S.	2015	Alzheimer's Disease Prevention: Where The Evidence Stands.	Review	No counselling/ psychotherapy<br>Focus on meditation
**100.**	Khayum, B.	2018	Toss the Workbooks! Choose treatment strategies for clients with dementia that address their specific life-participation goals.	Position paper	Wrong Intervention<br>No counselling/ psychotherapy
**101.**	Kiosses, D	2018	A path forward: A culturally relevant psychosocial intervention for depression in cognitive impaired older adults	Conference Abstract	Wrong Design
**102.**	Kiosses, D	2019	It's the path, not the destination: lessons learned from a psychosocial intervention for high risk depressed, cognitively impaired older adults	Conference Abstract	Wrong Design
**103.**	Koder, C.	2016	The use of cognitive behaviour therapy in the management of BPSD in dementia	Position Paper	Wrong Design
**104.**	Kok, R. M.	2017	Management of Depression in Older	Review	Wrong population but does highlight potential for psychotherapy in CI

Counselling People with Dementia – Excluded Articles					
	**First Author**	**Date**	**Title**	**Type**	**Reason for Exclusion**
			Adults		

Counselling People with Dementia – Excluded Articles					
	**First Author**	**Date**	**Title**	**Type**	**Reason for Exclusion**
**105.**	Laakkonen, M. L.	2016	Effects of Self-Management Groups for People with Dementia and Their Spouses	RCT	Wrong population but does highlight potential for Mindfulness in AD
**106.**	Larouche, E.	2015	Potential benefits of mindfulness-based interventions in mild cognitive impairment and Alzheimer's disease: an interdisciplinary perspective	Review	Wrong population but does highlight potential for Mindfulness in AD
**107.**	Larouche, E.	2019	Mindfulness mechanisms and psychological effects for aMCI patients: A comparison with psychoeducation.	RCT	Wrong population but does highlight potential for Mindfulness in AD
**108.**	Lee, D. W.	2018	Korean national dementia plans: from 1st to 3rd.	Grey<br>National Strategy	Not in English language
**109.**	Leung, P.	2015	Social support group interventions in people with dementia and mild cognitive impairment	systematic review	Wrong Intervention but one study Burgener (2008) multimodal therapy inc. CBT
**110.**	Lin, R.	2019	Effects of creative expression therapy on Chinese elderly patients with dementia	RCT	Wrong Intervention
**111.**	Ling, Y.	2018	A Randomized Controlled Trial with a Nine-month Follow-up of a Transdiagnostic Cognitive Behavioural Therapy (Group) for Chinese Adults	RCT (thesis)	Wrong population

Counselling People with Dementia – Excluded Articles					
	**First Author**	**Date**	**Title**	**Type**	**Reason for Exclusion**
			with Common Mental Disorders		

Counselling People with Dementia – Excluded Articles					
	**First Author**	**Date**	**Title**	**Type**	**Reason for Exclusion**
**112.**	Linnemann, A.	2017	Psychotherapy with mild cognitive impairment and dementia	Review	Wrong Design
**113.**	Lishman, E.	2014	The paradox of dementia: Changes in assimilation after receiving a diagnosis of dementia	Qualitative interviews	Wrong Intervention
**114.**	Livingston, G.	2017	Dementia prevention, intervention, and care	Review	Wrong Design
**115.**	Low, L. F.	2018	Do people with early stage dementia experience Prescribed Disengagement?	systematic review of qualitative studies	Wrong Intervention
**116.**	Low, L. F.	2018	Communicating a diagnosis of dementia: A systematic mixed studies review of attitudes and practices of health practitioners	systematic review	Wrong Intervention
**117.**	Mace, R. A.	2017	Therapeutic relationship in the treatment of geriatric depression with executive dysfunction	RCT	Wrong population
**118.**	Mantero, V.	2017	Genetic Counseling Dilemmas for a Patient with Sporadic Amyotrophic Lateral Sclerosis, Frontotemporal Degeneration &amp	Case Report	Wrong intervention
**119.**	Martínez-Alcalá, C.	2016	Information and Communication Technologies in the Care of the Elderly: Systematic Review of Applications Aimed	Systematic Review	Wrong intervention

Counselling People with Dementia – Excluded Articles					
	**First Author**	**Date**	**Title**	**Type**	**Reason for Exclusion**
			at Patients With Dementia and Caregivers.		

Counselling People with Dementia – Excluded Articles					
	First Author	Date	Title	Type	Reason for Exclusion
120.	McCabe, L.	2018	Scaffolding and working together: A qualitative exploration of strategies for everyday life with dementia	Secondary analysis qualitative interviews and focus groups	Wrong intervention
121.	McPherson, J. A.	2015	Evaluating the Need for Early Stage Alzheimer's Disease Patient-Caregiver Dyad Support Groups in Rural Washington.	Qualitative thesis	Wrong population
122.	McCurry, P.	2015	Living with the Challenges of Dementia	Book	Wrong Design
123.	Miller, S.	2015	Communicating Across Dementia: How to talk, listen, provide stimulation and give comfort	Book	Wrong Design
124.	Mela, C.	2017	The therapeutic model of group analytic psychotherapy in brain's plasticity modification and expression, , in patients with cognitive and psychiatric disorders: A hypothesis of neuron-immune-analysis and neuron-immune-modulation	Position Paper based on clinical research	Wrong population<br>The reduction of cytokine's levels following participation and psycho-education in Group Psychotherapeutic Treatment, could lead to a regulation of IL-1, a reduction of CRP, and amelioration of cortisol levels, thus regulating the inflammation of the brain. EEG and Mini Mental Scale significantly changed and improved after Psychotherapy.
125.	Mitchell, G.	2015	Person-centred care for people with dementia: Kitwood reconsidered	Position Paper	Wrong intervention but useful commentary

Counselling People with Dementia – Excluded Articles					
	**First Author**	**Date**	**Title**	**Type**	**Reason for Exclusion**
126.	Milby, E.	2015	Diagnosis disclosure in dementia: Understanding the experiences of clinicians and patients who have recently given or received a diagnosis	Qualitative IPA	Wrong Intervention<br>results support a psychosocial model of dementia and highlight the need for flexible follow up interventions that both recognise patients' use of avoidance and denial as coping strategies and facilitate social support
127.	Monteiro, J. N.	2015	Video care services: AAL solution for dementia support-state of the art of research and intervention Ambient Assisted Living	Book Chapter	Wrong Intervention
128.	Morgan, D.	2015	Availability and Primary Health Care Orientation of Dementia-Related Services in Rural Saskatchewan, Canada	x-sectional survey data	Wrong Intervention<br>Highlights dearth of counselling support services in rural Canada
129.	Nyman, S. R	2016	Meaningful activities for improving the wellbeing of people with dementia: beyond mere pleasure to meeting fundamental psychological needs	Review	Wrong Intervention
130.	Nakanishi, M.	2018	Psychosocial behaviour management programme for home-dwelling people with dementia	RCT	Wrong Intervention
131.	National Institute for Health and Care Excellence	2018	Dementia: Assessment, management and support for people living with dementia and their carers.	Guidelines	Wrong Intervention<br>No mention of counselling or psychotherapy
132.	National	2020	Dementia: Assessment, management	Updated	Wrong Intervention

Counselling People with Dementia – Excluded Articles					
	**First Author**	**Date**	**Title**	**Type**	**Reason for Exclusion**
	Institute for Health and Care Excellence		and support for people living with dementia and their carers.	Guidelines	No mention of counselling or psychotherapy

Counselling People with Dementia – Excluded Articles					
	**First Author**	**Date**	**Title**	**Type**	**Reason for Exclusion**
133.	National Institute for Health and Care Excellence	2018	Dementia Overview NICE Pathway	Overview of guidelines	Wrong Intervention<br>No mention of counselling or psychotherapy
134.	Ng, T.	2017	Mindfulness modulates biomarkers and cognition in elderly with mild cognitive impairment (mci)	RCT	Wrong Population
135.	Nicholson, L.	2017	Person-centred care: experiences of older people with dementia	Qualitative Literature review	Wrong Intervention<br>No mention of counselling or psychotherapy
136.	Nickel, F.	2018	Health economic evaluations of non-pharmacological interventions for persons with dementia and their informal caregivers	Systematic review	One relevant study (Spector et al. 2015) already included in review literature
137.	Nikumaa, H.	2019	Counselling of people with dementia in legal matters – social and health care professionals' role.	Reports on three Finish case studies	e assesses the legal security of people with dementia by concentrating on the social and health care professional's role in enabling their rights to be fulfilled by giving sufficient counselling and support
138.	Nyman, S.	2016	Meaningful activities for improving the wellbeing of people with dementia: beyond mere pleasure to meeting fundamental psychological needs	Review	Wrong Intervention<br>No mention of counselling or psychotherapy
139.	O'Caoimh, R.	2019	Non-pharmacological treatments for sleep disturbance in mild cognitive impairment		3 Mindfulness and CBT for Insomnia studies – original articles already included in review

Counselling People with Dementia – Excluded Articles					
	**First Author**	**Date**	**Title**	**Type**	**Reason for Exclusion**
			and dementia		

Counselling People with Dementia – Excluded Articles					
	**First Author**	**Date**	**Title**	**Type**	**Reason for Exclusion**
**140.**	Øksnebjerg, L.	2018	Towards capturing meaningful outcomes for people with dementia in psychosocial intervention research: A pan-European consultation	Consultation<br>Qualitative interviews	people with dementia wish to participate in interventions that enhance their well- being, confidence, health, social participation and human rights
**141.**	Olthof-Nefkens, M.	2018	Improving Communication between Persons with Mild Dementia and Their Caregivers: Qualitative Analysis of a Practice-Based Logopaedic Intervention	Qualitative	A short pragmatic but consistent approach for communication problems caused by dementia seems promising for improving daily communication and reducing stress and frustration
**142.**	Osvath, P.	2018	Information and communication technologies in the integrated care of people with dementia	Feasibility	Wrong Intervention
**143.**	Oyebode, J.	2016	Psychosocial interventions for people with dementia: An overview and commentary on recent developments	Review	Wrong Intervention – refs checked for counselling and psychotherapy PwD
**144.**	Parker, E.	2015	Dementia is a disease, not a person: exploring the experiences of people with dementia, carers and mental health practitioners on dementia and dementia services via a qualitative evaluation of a community-based memory service	Thesis<br>Qualitative Interviews	Wrong Intervention but highlights Counselling as important intervention for emotional support
**145.**	Quinn, C.	2016	A Review of Self-Management Interventions for People With Dementia and Mild Cognitive	Review	All studies outwith the date range.

Counselling People with Dementia – Excluded Articles					
	**First Author**	**Date**	**Title**	**Type**	**Reason for Exclusion**
			Impairment		

Counselling People with Dementia – Excluded Articles

	First Author	Date	Title	Type	Reason for Exclusion
146.	Quinn, C.	2016	A pilot randomized controlled trial of a self-management group intervention for people with early-stage dementia (The SMART study)	Pilot RCT Mixed Methods	Wrong Intervention<br>Facilitated group with various topics including psychotherapeutic components. Preliminary evidence that self-management may be beneficial for people with early-stage dementia
147.	Quintana Hernandez, D.	2015	Mindfulness-based stimulation in advanced Alzheimer's disease	Pilot RCT	Wrong Outcome Measures
148.	Ramsay-Jones, E.	2016	Being here with you: An examination of the relational field in dementia care	Qualitative Longitudinal psychoanalytical observation	Wrong Intervention but valuable insights into care experience<br>Focus on the experiences and relationships of two residents with dementia living in two different care homes (local government; private) Despite a focus on quality of care, our understanding of the relational field, upon which care practice is based, is underdeveloped
149.	Rao, G.<br>India	2020	Cognitive Therapy and Family Intervention for Patients with Dementia and Psychosis	Clinical guideline	Wrong Intervention but useful discussion on the potential role for psychosocial interventions in the management of persons with dementia particularly as relates to Indian context.
150.	Rehm, I.	2017	Cognitive behavioural therapy for older adults with anxiety and cognitive impairment: Adaptations and illustrative case study	Case Study	Wrong Design
151.	Reid, L. D.	2017	Cognitive behavioral therapy (CBT) for preventing Alzheimer's disease	Review	Wrong Population
152.	Rapaport, P.	2018	An intervention to improve sleep for people living with dementia:	Feasibility	Wrong Intervention – sleep promotion

Counselling People with Dementia – Excluded Articles					
	**First Author**	**Date**	**Title**	**Type**	**Reason for Exclusion**
			Reflections on the development and co-production of DREAMS:START (Dementia RElAted Manual for Sleep: STrAtegies for RelaTives)		

Counselling People with Dementia – Excluded Articles					
	**First Author**	**Date**	**Title**	**Type**	**Reason for Exclusion**
153.	Regan, M. (MHF)	2016	The interface between dementia and mental health: an evidence review.	Grey literature review and interviews with PwD	Wrong intervention<br>Comorbidities are underdiagnosed in people living with dementia, not extensively researched and therefore not understood fully. relatively little literature on the challenges or experiences associated with living with dementia and mental health problems nor on care needs in this context. lack of understanding within service provision and an absence of specialised services.
154.	Reichert, M.	2016	Mobile dementia counseling as low-threshold assistance for caregiving relatives	Feasibility Evaluation	? Wrong Population – Caregivers but also indicates for PwD in abstract. Full text in German
155.	Rentería, M.	2020	Genetic testing for Alzheimer's disease: Trends, challenges and ethical considerations.	Literature review	Wrong Intervention<br>Ethical concerns - Urgent need for protocol for genetic testing along with appropriate counselling
156.	Robinson, K. M.	2016	Outcomes of a Two-Component Intervention on Behavioral Symptoms in Persons With Dementia and Symptom Response in Their Caregiver	Quasi-experimental study	Wrong Population
157.	Roggenbuck, J.	2017	Genetic testing and genetic counseling for amyotrophic lateral sclerosis: An update for clinicians	Clinical Evidence	Wrong Design
158.	Sanford, S.	2018	Independence, loss, and social identity: Perspectives on driving cessation and dementia	Qualitative	Wrong Intervention but useful information on experience of loss findings reveal the profound effect of losses of autonomy associated with dementia as conveyed through the perspectives and experiences of family caregivers, and further

Counselling People with Dementia – Excluded Articles					
	**First Author**	**Date**	**Title**	**Type**	**Reason for Exclusion**
					demonstrate that such losses are shared between the person with dementia and their spouse and family members

Counselling People with Dementia – Excluded Articles					
	**First Author**	**Date**	**Title**	**Type**	**Reason for Exclusion**
**159.**	Schulc, E.	2016	Preventive home visits	Cross-sectional study	Wrong population<br>Nurse visits – Case management approach recommendation to given special attention to counselling and info for family members in preventive home visits<br>Full text in German
**160.**	Sivananthan, S. N.	2015	Caring for dementia: A population-based study examining variations in guideline-consistent medical care	Population based retrospective cohort study	Wrong Intervention<br>Patterns of inequality in respect of dementia care. High income participants more likely to receive better care including counselling support.
**161.**	Smith, C.	2015	Handbook of Depression in Alzheimer's Disease	Book	Wrong Intervention<br>Mainly Aetiology/PharmacologicalTreatment of depression except for Kiosses study (PATH) which is already included in the review
**162.**	Smythe, A.	2015	The experiences of staff in a specialist mental health service in relation to development of skills for the provision of person centred care for people with dementia.	Qualitative	Wrong Intervention<br>findings suggest that staff believe they learn through experience, doing and modelling themselves on peers who they perceive as having high standards, extensive experience and natural ability. Staff tend not to value classroom learning - training more likely to be successful if it includes strong elements of experiential learning through modelling alongside credible trainers using a problem based approach.
**163.**	Snow, K.	2015	Making sense of dementia: Exploring the use of the Markers of Assimilation of Problematic Experiences in Dementia scale to understand how couples process a diagnosis of dementia.	Qualitative	Wrong Intervention<br>The Assimilation Model (Stiles et al., 1990) was developed from psychotherapy process research to explore how change occurs during the therapeutic process

Counselling People with Dementia – Excluded Articles					
	**First Author**	**Date**	**Title**	**Type**	**Reason for Exclusion**
164.	Spreadbury, J. H.	2017	Measuring younger onset dementia: What the qualitative literature reveals about the 'lived experience' for patients and caregivers	Review Qualitative	Wrong Intervention<br>Important to recognise and address internal psychological processes such as finding effective coping strategies, developing acceptance of the diagnosis, managing grief and stigma, mastering transitions in self-perception of identity, and adjusting to a changed perception of the future. These are underestimated and future service provision should include addressing these types of psychological processes as an essential part of the lived experience
165.	Surampalli, A.	2015	Psychological Impact of Predictive Genetic Testing in VCP Inclusion Body Myopathy, Paget Disease of Bone and Frontotemporal Dementia	Pre-post test	Wrong population<br>In this small cohort, one third of individuals at 50 % risk chose pre-symptomatic testing. Although one quarter of those choosing testing had high anxiety at baseline, this was not evident at follow-up.
166.	Swaffer, K.	2014	Dementia and Prescribed Disengagement	Position Paper	Positive impact on being meaningfully and positively engaged is paramount to a person's well-being, their motivation to fight against the symptoms of dementia and to overcoming the emotional toll of diagnosis
167.	The British Psychological Society	2016	Psychological therapies for people with dementia	Evidence Briefing	Wrong Design
168.	Tible, O. P.	2017	Best practice in the management of behavioural and psychological symptoms of dementia.	Literature Review	Studies covered outwith date range. Three relevant – details recorded for one. Two already noted.

Counselling People with Dementia – Excluded Articles					
	**First Author**	**Date**	**Title**	**Type**	**Reason for Exclusion**
169.	Toms, G.	2015	A systematic narrative review of support groups for people with dementia	systematic narrative review	Wrong Intervention Supportgroupsseemacceptabletopeoplewithdementia.Qualitativestudiesreportsubjectivebenefits for participants but there is limited evidence of positive outcomes based on quantitative data. Samples have tended to be homogenous and this may limit the generalizability of finding
170.	Trivedi, D. P.	2018	Managing behavioural and psychological symptoms in community dwelling older people with dementia	systematic review of the effectiveness of interventions	Wrong Intervention – not focussed on counselling psychotherapy Nurses and occupational therapists appear to help people with dementia with behavioural and psychological symptoms, but professional comparisons are lacking and there is no shared language about or understanding of behavioural and psychological symptoms amongst professionals, or between professionals and family carers
171.	Tuijt, R.	2020	Exploring how triads of people living with dementia, carers and health care professionals function in dementia health care	Systematic qualitative review and thematic synthesis.	Wrong intervention but useful information Gives understanding of the intricacies of establishing a working care relationship between the members of a dementia care triad. This includes where members of the triad are agreed as well as when they differed, for example, when multiple professionals or carers are involved or when expectations are mismatched. HCP care providers can strengthen the dementia care triad by involving the person living with dementia, ensuring continuity of care and establishing expectations of care, effective communication and trust

Counselling People with Dementia – Excluded Articles					
	**First Author**	**Date**	**Title**	**Type**	**Reason for Exclusion**
**172.**	Turner, T.	2016	Dementia care: An overview of non-pharmacological therapies: A summary of non-pharmacological therapies for patients with dementia that may be used alongside licensed medicines	Literature Review	No mention of counselling or psychotherapy
**173.**	Vajda, A.,	2017	Genetic testing in ALS: A survey of current practices	Survey	Wrong intervention<br>Need for updated set of guidelines for presymptomatic genetic counseling and testing to people at high genetic risk for developing ALS. data suggest that the clinical application of genetic testing in symptomatic patients is not always evidence-based, and that genetic counselling of patients and families does not occur routinely as a standard of care in all instances
**174.**	van Boxtel, M.	2019	Mindfulness-based interventions for people with dementia and their caregivers: keeping a dyadic balance	Position Paper	Wrong Design
**175.**	Van der Linden, M.	2016	A life-course and multifactorial approach to Alzheimer's disease: Implications for research, clinical assessment and intervention practices	Review position paper	No mention of counselling or psychotherapy but interesting points re need for integrative approach.
**176.**	Volpe, U.	2020	Pathways to care for people with dementia: An international multicentre study	Retrospective study	Wrong intervention but useful data - Psychosocial interventions such as counselling/psychotherapy only offered in 2.7% of cases
**177.**	Veelenturf, A.	2017	Pharmacy team followed training for	Position paper	Written in German no full text available online

Counselling People with Dementia – Excluded Articles					
	**First Author**	**Date**	**Title**	**Type**	**Reason for Exclusion**
			counseling of patients with dementia: Communicating empathically		

Counselling People with Dementia – Excluded Articles					
	**First Author**	**Date**	**Title**	**Type**	**Reason for Exclusion**
**178.**	Verhülsdonk, S.	2017	Geriatric psychiatric home counseling for people with dementia and anosognosia: Results of a model project.	Grey – project model	Case management - Wrong intervention but may have useful content. Written in German
**179.**	Watson, J.	2016	Developing the Senses Framework to support relationship-centred care for people with advanced dementia until the end of life in care homes	Qualitative research	Imp. of embodied selfhood in dementia care - behaviours that may have previously been overlooked become noticed, increasing the scope and opportunities for interpersonal relationships and improved quality of care (Kontos & Martin, 2013). we can learn from care home staff through their experience of the human encounter with people with advanced dementia.
**180.**	Watson, J.	2016	Face to Face: Relating to people with dementia until the end of life in care homes	Qualitative research briefing paper	As above
**181.**	Weaks, D.	2015	Daring to tell: The importance of telling others about a diagnosis of dementia	Qualitative	participants recognised the need to tell others about their diagnosis but these conversations were difficult to initiate and manage, and hindered the processing of emotions
**182.**	Weatherby, T.	2018	Ethical and organisational considerations in screening for dementia	Position Paper	Wrong intervention
**183.**	Wells, J.	2020	The experiences of older adults with a diagnosed functional mental illness, their carers and healthcare professionals in relation to mental health service delivery	integrative review	Wrong population but useful background material<br>Older people with functional mental illness often perceived they did not have a mental health need

Counselling People with Dementia – Excluded Articles					
	**First Author**	**Date**	**Title**	**Type**	**Reason for Exclusion**
**184.**	Wells, R.	2019	Can Adults with Mild Cognitive Impairment Build Cognitive Reserve and Learn Mindfulness Meditation? Qualitative Theme Analyses from a Small Pilot Study	Pilot Qualitative	Wrong population
**185.**	Westerhof, G.	2019	Online therapy for depressive symptoms: An evaluation of counselor-led and peer-supported life review therapy	Pilot RCT	Wrong population
**186.**	White, K.	2018	Dementia: An Attachment Approach	Book	Wrong Design
**187.**	Whitehead, L.	2016	Psychological Treatments for Depression and Anxiety in Dementia and Mild Cognitive Impairment	Evidence Summary	Wrong Design
**188.**	Wicke, J.	2018	Differentiating Dementias, Delaying Decline: The Pharmacist's Role	Position Paper Continuing Education for Pharmacists	Wrong intervention Small section on counselling PwD from pharmacist perspective – highlights need for Pharmacists to have necessary skills and understanding
**189.**	Wolinsky, D.	2018	Diagnosis and Management of Neuropsychiatric Symptoms in Alzheimer's Disease	Review	Wrong intervention
**190.**	World Health Organisation	2017	Global action plan on the public health response to dementia 2017–2025	Strategy	No mention of counselling or psychotherapy
**191.**	World Health Organisation	2017	Integrated care for older people: guidelines on community-level interventions to manage declines in	Guidelines	No mention of counselling or psychotherapy for PwD

Counselling People with Dementia – Excluded Articles					
	**First Author**	**Date**	**Title**	**Type**	**Reason for Exclusion**
			intrinsic capacity		
**192.**	Wuthrich, V.	2015	Barriers to treatment for older adults seeking psychological therapy	Pre-post test	Extends understanding about the barriers to treatment in older adults with anxiety and depression. In this study, we identified significant barriers related to the identification of symptoms and in identifying the need for help in older adults
**193.**	Wuttke-Linnemann, A.	2019	Dyadic Wind of Change: New Approaches to Improve Biopsychological Stress Regulation in Patients with Dementia and Their Spousal Caregivers	Position Paper based on research evidence	Rationale for incremental benefits of considering dyadic processes among caregivers and PWD by means of elucidating underlying mechanisms. Emphasises need to evaluate underlying mechanisms by means of objective physiological stress markers in PWD and caregivers to give knowledge for development of tailored dyadic interventions.
**194.**	Zagorscak, P.	2018	Benefits of individualized feedback in internet-based interventions for depression	RCT	Wrong population
