# Supplementary material for: The role and impact of therapeutic counselling on the emotional experience of adults living with dementia: A systematic review
Source: Dementia (London). 2024 Apr 16;23(5):882–902. doi: 10.1177/14713012241233765 (PMC11163847; doi:10.1177/14713012241233765)
Supplement: Supplemental Material - The role and impact of therapeutic counselling on the emotional experience of adults living with dementia: A systematic review [file sj-pdf-4-dem-10.1177_14713012241233765.pdf]

Table 3. CASP QUALITY APPRAISAL SCORING TABLES FOR RAPID REVIEW: Counselling adults with dementia

Counselling Adults with Dementia
Quality questions: Were the following areas adequately addressed or appropriately applied?
0= not or inadequately addressed/applied; 1= adequately addressed/applied; 2= well addressed/applied
Scores: 0-7= low quality; 8-14= moderate quality; 15-20= high quality.

Table 1. QUALITATIVE LITERATURE: Key issues relating to counselling adults with dementia: a review of on the role and impact of therapeutic counselling on the emotional experience of adults with dementia.

	Author/ year/country	Aim/ Focus	Mthdlgy	Design/ methods	Rcrtmnt strategy	Data Collctn	Bias/ Rflxvty	Ethical issues	Data analysis	Statement of findings	Value/ applcblty	Score (n=20)
1.	**Baker 2022**	2	1	1	1	1	1	2	2	2	2	15
2.	**Birtwell 2018**	2	2	1	0	1	0	2	1	2	2	13
3.	**Cheston 2018**	2	2	2	1	1	1	1	2	1	1	14
4.	**Cheston 2017**	1	2	2	0	1	0	1	1	1	1	10
5.	**Douglas 2021**	2	1	1	1	1	1	2	1	2	2	14
6.	**Erdman 2016**	2	2	2	1	2	1	1	2	2	2	17
7.	**Griffiths 2021**	2	0	1	0	1	0	1	1	2	1	9
8.	**Hsiao 2016**	2	1	1	1	1	1	1	2	2	1	13
9.	**Johnston 2017**	2	1	1	1	2	0	1	1	2	2	13
10.	**Luxmoore 2017**	2	1	1	1	0	1	0	0	1	1	8
11.	**Lykkeslet 2016**	2	1	2	1	2	0	1	1	1	2	13
12.	**McCombie 2021**	2	1	1	1	1	1	2	1	1	1	12
13.	**Perren 2018**	1	1	1	0	1	0	1	1	1	1	8
14.	**Pybis 2021**	2	1	1	1	1	0	1	1	1	2	11
15.	**Staubo 2017**	2	1	1	0	1	0	1	1	1	1	10

Table 2. RANDOMISED CONTROLLED TRIALS Key issues relating to counselling for adults with dementia: a review of on the role and impact of therapeutic counselling on the emotional experience of adults with dementia. Quality appraisal of ***RCT literature***

	Author	Aim/ Focus	Rndmstn process	Attrition	Blinding	Comprblty participants	Equtbility – Active control	Treatmnt effect	Generalisab ility	Reporting of outcomes	Ethics/ Harm and cost benefit	Score (n=20)
1.	**Bailey 2017**	2	0	1	0	2	1	1	1	1	0	9
2.	**Jenewein 2021**	2	2	2	1	2	2	2	1	2	2	18
3.	**Kiosses 2015**	2	2	2	1	2	1	2	1	2	1	16
4.	**Kiosses 2015 (Suicidal Ideation)**	2	1	2	1	2	1	1	1	2	1	14
5.	**Koivisto 2016**	2	2	2	1	2	0	1	1	1	2	14
6.	**Marshall 2015**	1	1	2	1	2	0	1	1	2	2	13
7.	**Spector 2017**	2	2	1	1	1	0	1	1	2	2	13
8.	**Tonga 2021**	2	2	1	1	2	2	2	1	2	2	17
9.	**Whitlatch 2019**	2	1	2	1	1	1	1	1	1	2	13

Table 3. OBSERVATIONAL STUDIES: Key issues relating to counselling for adults with dementia: a review of on the role and impact of therapeutic counselling on the emotional experience of adults with dementia.

	Author	Aim/ Focus	Recruitment	Bias	Confounders identified	Confou-nders (Design/ Analysis)	Follow-up (level of completio n)	Follow-up (duration)	Results	Precision of results	Credibility	Value/ applicabili ty	Fit with existing evidence	Implicatio ns for practice	Score Total (n=26)
1.	**Alzubaidi 2020**	2	2	2	0	1	2	0	2	1	2	1	1	1	17
2.	**Bhattacharjee 2017**	2	1	1	1	1	0	0	2	1	2	1	1	1	14
3.	**Cornelis 2018**	2	1	1	1	2	1	0	1	1	1	1	1	1	14
4.	**Jo 2016**	2	0	0	0	0	2	0	1	1	1	1	1	1	10
5.	**Jennings 2016**	2	2	1	0	0	2	1	1	1	1	1	1	1	14
6.	**Orsulic-Jeras 2016**	2	1	0	0	0	2	0	1	1	0	1	1	1	10
7.	**Skov 2022**	2	2	0	1	0	1	1	1	1	1	1	1	1	13

Table 4. FEASIBILITY STUDIES Key issues relating to counselling for adults with dementia: a review of on the role and impact of therapeutic counselling on the emotional experience of adults with dementia. Quality appraisal of ***Feasibility literature***

	**Author/ year/country**	**Aim/ Focus**	**Mthdlgy**	**Design/ methods**	**Rcrtmnt strategy**	**Data Collctn**	**Bias/ Rflxvty**	**Ethical issues**	**Data analysis**	**Statment findings**	**Value/ applcblty**	**Score (n=20)**
1.	**Berwig 2020**	2	2	1	1	1	1	2	2	2	2	16
2.	**Blair 2018**	2	1	1	0	1	0	1	0	1	1	8
3.	**Cheston 2015**	1	0	0	1	1	0	1	1	1	1	7
4.	**Churcher Clarke 2017**	2	2	2	1	1	1	2	2	2	1	16
5.	**Collins 2018**	2	2	1	1	1	2	1	2	2	2	16
6.	**Craig 2018**	2	1	1	1	1	0	1	1	1	2	11
7.	**Garcia-Alberca 2017**	1	1	0	0	0	0	1	1	1	1	6
8.	**Johnston 2015**	1	1	1	0	1	0	2	0	2	2	10
9.	**Kovach 2018**	2	1	1	1	1	1	1	2	1	1	12
10.	**Paller 2015**	2	0	1	1	0	1	2	1	2	2	12
11.	**Plunger 2019**	2	2	2	2	2	1	1	1	2	1	16
12.	**Tonga 2016**	2	0	1	0	1	0	0	1	1	1	7
