# Supplementary material for: The role and impact of therapeutic counselling on the emotional experience of adults living with dementia: A systematic review
Source: Dementia (London). 2024 Apr 16;23(5):882–902. doi: 10.1177/14713012241233765 (PMC11163847; doi:10.1177/14713012241233765)
Supplement: Supplemental Material - The role and impact of therapeutic counselling on the emotional experience of adults living with dementia: A systematic review [file sj-pdf-5-dem-10.1177_14713012241233765.pdf]

Counselling Adults with Dementia: a review of on the role and impact of therapeutic counselling on the emotional experience of adults with dementia
TABLE 4: Characteristics Qualitative Studies

Author/ Date/ Country	Study Aim	Study Design/ Theoretical perspective/ Reflexivity	Participants	Intervention and context/setting	Attrition	Key Findings/ concepts/themes	Recommendations	Outcome Type & Perspectve
Baker, S. 2022 England	To gain a better understanding of the potential facilitators and barriers to accessing and engaging with CBT for people living with dementia or MCI as perceived by clinicians working in primary care psychological therapies services.	Interview study using convenience sampling based on availability and first come recruitment basis<br><br>Face-to-face structured interviews with 14 clinicians<br><br>Thematic analysis (NVivo to support coding) adopting realist/essentialist position; Member validation	Psychological therapists (n=14; F10:M4) - Psychological Well being Practitioners (n=7); CBT therapists (n=5);Clinical psychologists (n=2) working within Improving Access to Psychological Therapy (IAPT) services<br>Avge of 2 yrs experience excepting one practitioner with 15yrs exp.<br>50% had worked with people with dementia but only 2 had received dementia specific training	Eligible participants were qualified psychological therapists working within IAPT services, an initiative to increase the national availability of Primary Care therapy services in England (Clark, 2011).<br><br>Recruited through primary care psychological therapies services Advertised by emailing a recruitment poster addressed to the managers or admin team of 28 IAPT services within London		Three themes:<br>1.'attitudes towards dementia'<br>2.'competing demands'<br>3.'pressure without support'<br><br>Therapists in primary care psychological therapy settings face particular challenges when delivering cognitive behavioural therapy (CBT) to people living with dementia or mild cognitive impairment.<br>Experience of successful engagement and outcomes for older adults support positive clinician attitudes towards working with people with cognitive impairments.	Adapting CBT therapy to meet the needs of individuals is an important facilitator for both older adults and people living with dementia or mild cognitive impairment<br><br>Staff support in terms of time, supervision and adequate resources are needed to ensure flexibility and quality of service.	Secondary Professional
Birtwell, K. 2018	To explore attitudes to, and	Coproduction design - patient and public	All participants (n=5) had received a	Experience of: being diagnosed;	None reported	Three main themes:	Talking therapies and psychosocial	Primary User

Author/ Date/ Country	Study Aim	Study Design/ Theoretical perspective/ Reflexivity	Participants	Intervention and context/setting	Attrition	Key Findings/ concepts/themes	Recommendations	Outcome Type & Perspectve
England	acceptability of, psychological support* for people with mild dementia, from their perspective.<br><br>* The term psychological support included counselling, psychotherapy, other one-to-one psychological interventions [such as cognitive behavioural therapy (CBT)], group interventions (including mindfulness) and support from other sources	involvement in research process<br><br>Semi-structured qualitative interviews<br><br>Inductive thematic analysis (Braun & Clarke, 2006)<br><br>Reflexive statements	diagnosis of dementia with Alzheimer's disease or vascular dementia.<br><br>(M1:F4)<br>Age range 58–79yrs<br><br>White British (n=3); African Caribbean (n=2)	support services; opinion of alternative support options<br><br>Participants recruited from the Memory Assessment Services and the Young Onset Dementia Service of an NHS Mental Health Trust<br>time since diagnosis (2-21 months)		1.Loss of physical abilities associated with loss of identity and place in the community;<br>2.Coping mechanisms - asserting control, and growth and development, including engaging in new activities or using humour;<br>3.Support - Individual needs and preferences key to effective support. Value placed on social support and opportunities to talk about feelings. Counselling, mindfulness and group-based activities viewed positively. Support from admiral nurses or specialist nurses tended to be viewed in terms of physical health needs, and most needed in later stages of dementia	interventions seen as acceptable supports.<br>Person-centred support, tailored to individual needs/ preferences/abilities and experience of dementia.<br>Psychosocial interventions - counselling, group-based interventions, mindfulness, support groups, walking and gardening to help people with dementia to build a new social identity that embraces self-agency and efficacy.	
Cheston, R.<br>2015<br>England	To identify whether there were changes in participant	Two-part study – qualitative sub-component of a Pilot RCT:	Diagnosis of Alzheimer's disease, vascular dementia or dementia with	LivDem Groups (n=7)<br>8 weekly (75min)	Attendance overall – 83%	Study 1 indicated a process of discursive change in the ways	Give attention to, and sensitively support the emotional	Primary User and Secondary Professional

Author/ Date/ Country	Study Aim	Study Design/ Theoretical perspective/ Reflexivity	Participants	Intervention and context/setting	Attrition	Key Findings/ concepts/themes	Recommendations	Outcome Type & Perspectve
	discourse about dementia over the course of participation in Living Well with Dementia (LivDem) groups;<br><br>To establish any differences in clinical outcomes between groups run from two separate locations;<br>To examine for contrast in therapist behaviour across two groups.	Analysis of verbal content from the LivDem groups using:<br><br>Markers of Assimilation of Problematic Experiences of Dementia (MAPED)<br><br>Hill Counsellor Verbal response rating scale (for analysis of therapist verbal behaviour)	Lewy bodies within previous 18 months (n=60) randomised to intervention or TAU<br><br>Participants recruited from primary care teams, and the local Memory clinic	Carers attended first and last sessions<br><br>Each group had 5-7 participants Person with dementia (n=28)		participants framed their dementia diagnosis and experience over the intervention duration pointing to an increase in emotional processing.<br>Study 2 pointed to worse outcomes in week 8 where facilitators adopted a more directive approach and placed emphasis on information giving in the early programme stages.	processing of participants' coming to terms with their dementia diagnosis.<br><br>Avoid directive facilitator behaviour as this may be linked to poorer outcomes	
Cheston, R.<br>2017<br>England	To identify whether there were changes in participant discourse about dementia over the course of one group programme	Two-part study (as above):<br>Analysis of verbal content from the LivDem groups using:<br><br>Markers of Assimilation of Problematic Experiences of Dementia (MAPED) – 125 extracts examined from 8 randomly chosen group sessions (not 1	Diagnosis of Alzheimer's disease, vascular dementia or mixed within previous 18 months (n=60)	LWWD Group (one of 7 groups in the study)<br><br>8 weekly (75min) sessions with carers at first and last<br>Participants (n=5)	(n=1) Analysis based on four participants. One dropped out of the study	Over half of the extracts (66 of the 125) came from one participant – when excluded MAPED shows that in the second half of the group, participants talked about their dementia in a different way to that in the first four sessions – greater acceptance –	LivDem groups can help with the emotional processing of dementia diagnosis and experience.<br><br>Further work needed to assess the therapeutic value of LivDem groups	Primary User

Author/ Date/ Country	Study Aim	Study Design/ Theoretical perspective/ Reflexivity	Participants	Intervention and context/setting	Attrition	Key Findings/ concepts/themes	Recommendations	Outcome Type & Perspectve
		or 10)<br><br>Hill Counsellor Verbal response rating scale (for analysis of therapist verbal behaviour)				inferred positive emotional processing		
Douglas, S. 2021 England	To investigate the experiences of people with depression and dementia who participated in the mindfulness-based cognitive therapy intervention and those of their carers and facilitators. Research questions: (1) How do participants experience the MBCT course? (2) Which aspects of the course are perceived to useful? (3) What are the perceived effects of the course amongst participants, their carers and facilitators? (4) How do carers	Nested within a randomised controlled feasibility study<br><br>Semi-structured individual interviews at NHS sites, participants' homes or by telephone within 4 months post MBCT intervention.<br><br>QSR NVivo7 Thematic Analysis	Person with dementia, 60 years or older who met criteria for mild depression (Patient Health Questionnaire [PHQ-9]) (n=8) Carers (n=6) recruited from memory services in two National Health Service (NHS) trusts in the UK	MBCT protocol for the prevention of depression relapse (Segal et al., 2002) adapted for people with dementia	80% attended 7 sessions or more. Adherence to home practice inconsistent	Identifying the experience of taking part in the MBCT group course and important key ingredients of it<br>Effects and outcomes of the course<br>Factors influencing engagement<br>Adaptations to the course<br>Challenge of home practice<br><br>Small study/Risk of researcher bias/interview timing	Weekly phone calls targeted to support home practice; additional materials, e.g. video demonstrations and notes for home use; Offer introductory information to participants and carers over 2 sessions; Facilitators would benefit from getting to know participants better in advance to inform individualised adaptations; Longer interventions with f.up support; Remind participants of the purpose and set expectations for each session at its start	User and professional

Author/ Date/ Country	Study Aim	Study Design/ Theoretical perspective/ Reflexivity	Participants	Intervention and context/setting	Attrition	Key Findings/ concepts/themes	Recommendations	Outcome Type & Perspectve
	support home practice? and (5) What adaptations are required to make MBCT suitable for this client group?							
Erdmann, A. 2016 Germany	To develop a case study on the use of Integrative Validation Therapy (IVA) within a Long-term Care Facility, generate hypotheses about the effects of IVA on people with dementia, their relatives and their nurses, and to explore how IVA is accepted by nurses and relatives of people with dementia<br><br>Research Qs:<br>1.With which categories can we describe the practice of Integrative	Fourth Generation Evaluation (Guba & Lincoln, 1989) Constructivist - hermeneutic-dialecticism - descriptive and exploratory design - structured framework for evaluation of new programs, processes, organisations.<br><br>Data Collection: Problem-centred interviews with professionals/family carers (n=17); observation of interactions between Integrative Validation Therapy (IVA) practitioners and people with dementia during morning or afternoon care; focus groups (FG) (n=2) – with 1) IVA practitioners and one IVA trainer from the	IVA trained Nurses (n=2); IVA practitioners of the nursing home (n=7); relatives of nursing home residents (n=(7); treating physician (n=1)	IVA with residents (n=61) people with dementia living in a long-term care facility (LTCF) in small village in Germany Consent from 4 residents for observation	None reported	IVA contains a specific attitude toward people with dementia and a few other components of action: perception, validation, evaluation and documentation.<br>IVA for persons with dementia: an experience of trust, belonging, attachment; perception of own identity; the feeling of the person that somebody understands his or her situation and feelings; the opportunity to derive comfort from somebody, an experience of appreciation, respect, well-being and joy; less behaviour	IVA can help the process of transition into LTCF for people with dementia.<br><br>Professionals need to: be aware of embodied self of the person with dementia and accept their subjective reality/ modes of expression; be congruent and practice empathy to help people with dementia to self-express and validate selfhood; support self-esteem and help people with dementia to restore personal identity	Primary and Secondary Professional/ Carer

Author/ Date/ Country	Study Aim	Study Design/ Theoretical perspective/ Reflexivity	Participants	Intervention and context/setting	Attrition	Key Findings/ concepts/themes	Recommendations	Outcome Type & Perspectve
	Validation Therapy (IVA)?<br><br>2. How is IVA evaluated by nurses and relatives?	participating nursing home and, 2) a FG session with two IVA trainers from the participating nursing home, 14 other IVA trainers from Germany and Switzerland and the originator of the Feil method – Richard (1994).<br>Data Analysis: Qualitative content analysis using transition theory (Meleis, 2010) and the theory of Person-centred care (Kitwood, 2012).<br><br>Member validation/Reflexivity				disturbances like agitation or aggressive behaviour; reduced consumption of benzodiazepine and neuroleptics, for example risperidone.		
Griffiths, A. 2020 England	To understand the experiences of individuals with dementia or caring for someone with dementia, before and after a 12-week relational counselling intervention delivered through a third sector organisation	Semi-structured interviews<br><br>Framework analysis	Pre-intervention (n=29)<br>Person with dementia (n=6)<br>Carers (n=23)<br>Included 3 Dyads<br><br>Post-intervention (n=25)<br><br>F (n=22)<br><br>White British (n=26)<br><br>Avge age of person	Counselling intervention ran for 18 months within a faith-based community organisation staffed by one counsellor offering a course of 12 weekly counselling sessions lasting 1 hour. Counselling recipients were referred to the service through	N=4	Four main themes identified; expectations and outcomes of counselling, emotional impact of life with dementia, appraisals of identity and importance of therapeutic relationship	Participants reported that counselling interventions addressed a range of needs and concerns that they had, enabling them to reassess and reconsider these. Specific training is needed before therapists deliver therapeutic interventions with	Primary User

Author/ Date/ Country	Study Aim	Study Design/ Theoretical perspective/ Reflexivity	Participants	Intervention and context/setting	Attrition	Key Findings/ concepts/themes	Recommendations	Outcome Type & Perspectve
	within England.		with dementia (n=81yrs)	their family doctor or third sector agencies			people with dementia, to ensure that appropriate support is provided for those with more severe cognitive impairment or who may have fluctuating capacity. Future research should explore the experiences of people with dementia and their caregivers, across different counselling modalities, to establish the appropriateness and effectiveness of relational counselling	
Hsiao, H.-Y.<br>2016<br>China	To examine similarities and differences in dementia caring among Mental Health providers (MHP) in city versus town settings	Purposive sampling Data collection: Video recorded Focus groups (n=4);<br>and a brief survey to gather sociodemographic information on MHP.<br><br>Framework analysis	MHP (n=40) in Xicheng (n=20, mean age – 33yrs) and Daxing (n=20, mean age – 33.5yrs)<br><br>Mean yrs. work experience: Xicheng (10); Daxing 10.5)	Investigation into: 1)sociodemographic characteristics and work experiences of MHP; 2) their knowledge of dementia as a disease; 3) MHP attitudes toward dementia; 4) clinical practices for people with dementia, and; 5) experiences of working with	None reported	Disparities between knowledge, skills, clinical practices and resources specific to dementia care between urban (Xichen) and town/rural (Daxing) settings.<br>Uneven distribution of medical and human resources weighted towards urban setting. Main	Need for: Collaborative dementia care model involving academic institutions, communities, and mental health providers; In-depth perspective on training needs for MHP; more qualitative research on process-type issues; larger samples in city and	Secondary Professional

Author/ Date/ Country	Study Aim	Study Design/ Theoretical perspective/ Reflexivity	Participants	Intervention and context/setting	Attrition	Key Findings/ concepts/themes	Recommendations	Outcome Type & Perspectve
				family caregivers.<br><br>Researchers from the Institute of Mental Health in Beijing chose Xicheng and Daxing as two contrasting districts in Beijing city to represent the city and town; these districts were chosen from 16 administrative districts to collect data		similarities across the two groups:<br>1. insufficient training and education about dementia; 2. therapeutic nihilism; 3. ageism; 4. need for specialist care; 5. low awareness of dementia among family caregivers; and 6. discriminatory attitudes and behaviour toward people with dementia	town settings could examine mental health providers' knowledge, attitudes, and clinical practice relevant to dementia care<br><br>Information and advice on dementia requires to reflect the person's sociocultural background.	
Johnston, B. 2017 Scotland	To explore and examine the thematic features and shared narrative dimensions of Dignity Therapy (DT) documents, produced as part of a feasibility study, in order to gain a fuller understanding of the value of using DT for people with early stage dementia	DT question framework Interviews<br><br>In-depth thematic analysis of DT generativity document using Framework analysis (Ritchie, Lewis, Nichols, & Ormston, 2013)	People with early stage dementia, (n=7)<br>71 to 82 yrs<br>5M:2F<br>AD (n=3)<br>VD (n=3)<br>AD & VD (n=1)<br><br>recruited from a post diagnosis dementia service in East Scotland, which provides support for up to one year for people with early stage dementia* (moderate cognitive decline, with potential difficulty	Dignity Therapy process/creation of<br>Dignity Therapy documents (n=7)	None reported<br>No adverse events	Four main themes:<br>Origin of values;<br>Essence and affirmation of self;<br>Forgiveness and resolution;<br>Existentialism/ meaning of life<br><br>DT offered a key to connect with the individual, potentially facilitating personalised rather than systemised care.<br><br>Allows personal story to be told and	Important to emphasise those life events that are most important to the individual rather than the dementia story.<br><br>DT shows promise as a short psychotherapeutic intervention related to legacy and personhood. DT can make a valuable contribution to the support of people with dementia by providing	Primary<br>User

Author/ Date/ Country	Study Aim	Study Design/ Theoretical perspective/ Reflexivity	Participants	Intervention and context/setting	Attrition	Key Findings/ concepts/themes	Recommendations	Outcome Type & Perspectve
			concentrating, and decreased memory of recent events (Reisberg *et al.,* 1982)			personal legacy and history to be preserved<br><br>Highlights key relational skills of the Dignity Therapist	information about their values, their self-identity, and the people or events that have been important to them	
Luxmoore, B. 2017 England	To understand appropriate approaches to communicating with people with advanced dementia to facilitate an increased understanding of their experiences.	Auto-ethnography Audio recordings /Structured note taking post interviews/reflexivity supported by mentalisation (Allen and Fonagy, 2006), a psychodynamic concept that aims to help with understanding emotional responses to others.<br><br>Mentalisation used to observe the author's emotional responses to people with dementia (focus on critical incidents) and influence on their responses/ communicative interactions	People with advanced dementia: in residential care setting (n=7) (16hrs); Domestic setting (n=1) (14hrs)	Account of the first author's experiences working as a project manager on a project designed to solicit the views and understanding of the lived experience of people with advanced dementia.	None reported	Themes:<br>An uninvited guest; I am afraid I am going to have to get going soon; Working the room; Maggie and Matthew<br><br>Points to potential role of mentalisation in developing natural and authentic strategies to support family carers. Non-mentalizing responses may be a root cause of mis-understanding and emotional disengagement.	Promote the skills of mentalising to support empathic, and Person-centred responding to the person with dementia	Primary User Secondary Professional
Lykkeslet, E. 2016	To investigate the healthcare providers' (HCP)	3-phase Action Research: Participant	People with advanced dementia (n=8) 1M:7F living in	The change process was based on	Person with dementia	Staff change process described as: 1. from	MMC can support and enhance HCPs understanding and	Primary User and Secondary

Author/ Date/ Country	Study Aim	Study Design/ Theoretical perspective/ Reflexivity	Participants	Intervention and context/setting	Attrition	Key Findings/ concepts/themes	Recommendations	Outcome Type & Perspectve
Norway	experiences with introducing Marte Meo Counselling (MMC) in a dementia-specific care unit	Observation and Focus Groups (FG) (n=4)<br><br>Phase 1) mapping using participant observation (n=50hrs) over 5 months; FG 1;<br><br>Phase 2) intervention - lectures, clinical supervision in Marte Meo Counselling (MMC) and reflection groups (n=7) over 12 months. FGs 2 and 3 pre and post; 3) evaluation using participant observation (n=48hrs) over 6 months, FG4<br><br>Thematic analysis (Braun and Clark, 2006)	a 8-person, dementia care unit. ≥80yrs<br>Experienced Staff (n=8)<br>2 Nurses<br>6 Care Assistants<br>All female	implementation of new perspectives for communication and interaction based on MMC, including video recording (1 day a week for 11 weeks followed by supervision) of complicated situations in daily practice to help change practice in deadlocked relational situations between residents and HCPs.	who Died (n=3)<br>Replaced by 3 new residents<br>No staff changes/ all participated	challenging behaviour to challenging interaction; 2. from generalised skills to contextualised relational care; 3. from personal challenges to a professional community.<br><br>Helped HCPs to become active participants in person with dementia's world and changed perception of challenging behaviour as a disorder to being a challenge to their modes of interaction	empathic relating to person with dementia in Care Home context.<br><br>MMC should be combined with continuous reflection to improve healthcare providers' interaction with people who live with dementia.<br><br>Phenomenological research valuable paradigm to see the lifeworld of the person with dementia	Professional
McCombie, A. 2021 Canada	To adapt and optimize problem adaptation therapy (PATH) for depression in dementia by grounding it in the lives of people with dementia, caregivers and clinicians.	Person-centered qualitative approach to elicit the unique cognitive, psychological and social needs of people with dementia relevant to the adaptation of the intervention. Two-stage design: 1) interviews and focus groups to identify	People with mild to moderate dementia (n=10)<br>Caregivers (n=9) recruited from Memory Clinics and through Community Mental Health Teams<br><br>PPI focus group - People with dementia (n=6)	Problem adaption therapy (PATH) adapted for depression in dementia – two session trial of adapted manualised approach in participant's own homes	50%<br><br>Declined Stage 2 (PATH) (n=5 people with dementia) and Carers (n=5)	Stage 1: Main themes:<br>1.Experience of loss;<br>2.Pertinent cognitive and emotional aspects of depression in dementia<br>3.Caregiver involvement challenges	Supporting people with dementia to continue with valued roles but also adjust to changes; supporting open discussion with caregivers around activities and care arrangements to optimise independence and	Primary User Secondary Professional

Author/ Date/ Country	Study Aim	Study Design/ Theoretical perspective/ Reflexivity	Participants	Intervention and context/setting	Attrition	Key Findings/ concepts/themes	Recommendations	Outcome Type & Perspectve
		priorities and concerns surrounding depression in dementia; 2) Adapting and trialling the adapted intervention. Stage 1: Individual interviews- (n=10 people with dementia) Caregivers (n=9) Focus Groups - Healthcare practitioners and clinical academics with experience of working with dementia (n=35) Public interest group (PPI) group (n=6 people with dementia)<br><br>Stage 2: Modified intervention using Think-aloud sessions (n= 5 people with dementia; 5 Carers)<br><br>Thematic analysis (Braun and Clark, 2006)	Healthcare professionals (n=35) recruited from Memory Clinics and Community Mental Health Teams in North London and staff/specialists from the PATHFINDER research study			4.Preferences for therapy delivery'<br><br>Feedback from participants used to adapt the PATH manual and training for PATH therapists including session structure, information and instructions, wording, adjusting information and instructions for people with different levels of dementia.<br><br>Stage 2: Participants reported many positives of the sessions, including having practical suggestions and approaches to problems, a chance to talk through things and make plans, and that it left them with a sense of hope for the future.	reduce relationship tensions and fears around burden; supporting people with dementia to maintain social activities to create a supportive community and increase confidence and self-esteem.	
Perren, S. 2018 England	To evaluate clinicians' views of the impact of conducting inpatient	Interpretative Phenomenological Analysis (IPA) Smith and Osborn (2008)	Psychotherapists (n=3) Staff nurse trained in psychotherapy (n=1) experienced in	Weekly psychotherapy groups running in the older adults' units at the	None	1. The intra-personal: The facilitators' experience of the group; 2. The inter-	Need for adaptation from traditional psychotherapy group to embrace moment to moment	Secondary Professional

Author/ Date/ Country	Study Aim	Study Design/ Theoretical perspective/ Reflexivity	Participants	Intervention and context/setting	Attrition	Key Findings/ concepts/themes	Recommendations	Outcome Type & Perspectve
	psychotherapy groups for older adults with enduring mental health issues and/or cognitive impairment.	Focus Group (n=4)<br><br>Member Validation with one member of the FG	running psychotherapy groups for people with dementia (n=4)	Retreat Hospital, York:<br>Women's groups (n=2);<br>Men's group (n=1)		personal: The interactional experience of the group; 3. The extra-personal: The external experience of the group.<br>The capacity and desire of group members to communicate and belong challenged their own views (and ageism) and that of staff members. The facilitators' experience was of the beneficial and normalising effects of these groups in enabling and enhancing communication, emphasising common humanity, facilitating the expression of emotion and combating isolation by promoting a sense of belonging.	flexibility	

Author/ Date/ Country	Study Aim	Study Design/ Theoretical perspective/ Reflexivity	Participants	Intervention and context/setting	Attrition	Key Findings/ concepts/themes	Recommendations	Outcome Type & Perspectve
Pybis, J.<br>2021<br>England	To explore the experience of counsellors and views of care home managers and care teams towards counselling in care homes to better understand the barriers and facilitators of implementing counselling within a care home	Semi-structured interviews conducted by telephone<br>Thematic analysis (Braun and Clarke 2006)<br>No theoretical alignment – data driven	The British Association for Counselling and Psychotherapy BACP registered counsellors (n=12) with experience of counselling within the care home setting.<br><br>(M:1F9),gender unknown (n = 2)<br><br>British (n = 9; other n = 1; unknown n = 2)<br><br>Mean age 58.5 yrs. Mean counselling and working with older people experience (n=14yrs)<br><br>Care home managers (n = 3) and staff (n =6) were recruited via ENRICH (Enabling Research In Care Homes	Participants recruited via an advert placed on the BACP website, member e-bulletin, and social media platforms		1) the funding and referral process for counselling in a care home - no tried and tested service delivery model for the provision of counselling; 2) the skills and competences required – training gap – few trained counsellors to work in this context; 3) training and supervision needs; 4) adaptations to practice and 5) barriers to implementing counselling in a care home – funding support; care home staff; practical issues – readiness of client/staff interruptions; family dynamics; generational perceptions of counselling/stigma	Specialised training for counsellors and care home staff<br>Importance of: staying with client's reality; reducing intervention time; using creative methods; risk linked to counselling impact;<br>Adaptations to tailor therapy to individual needs; flexibility; "elastic boundaries"<br>Financial support<br>Working collaboratively with care staff – continuity and regularity of counsellor attendance<br>Mature counsellors	Secondary<br>Professional

Author/ Date/ Country	Study Aim	Study Design/ Theoretical perspective/ Reflexivity	Participants	Intervention and context/setting	Attrition	Key Findings/ concepts/themes	Recommendations	Outcome Type & Perspectve
Staubo, H. 2017 Norway	To assess how an intervention impacted on subjects' everyday lives and quality of life.<br>To describe the interaction between the subjects and the therapist.	Hermeneutical phenomenology<br>Case study – notes logged following each meeting (n=11)<br>Content Analysis<br><br>Montgomery and Åsberg Depression Rating Scale (MADRS) to map depressive symptoms, and the Quality of Life in Alzheimer's Disease Scale (QOL-AD)<br><br>(sub-study from CORDIAL a manual-based, intervention RCT study that includes 200 people living at home with early stage dementia and their families. KORDIAL aims to assess how the intervention impacts on subjects' everyday lives and quality of life)	People diagnosed with Alzheimer's Disease (AD) in the last twelve months and living at home, and their families (n=2);<br>Therapist (n=1)	CORDIAL , cognitive rehabilitation and cognitive-behavioral treatment (CBT) for early dementia in AD<br><br>Focus on meaningful activities aimed at modifying depression, aiding memory/reminiscence<br><br>11 sessions undertaken at a memory clinic in a university hospital	None	Marked variation in the relational interaction between the two people with dementia and the therapist. Therapist attributed this to the subject's insight into the disease, appropriateness of the intervention, and motivation to take part.<br>For one subject, the intervention showed a positive impact on their everyday life and quality of life.<br>Family member participation seemed to be beneficial and supportive for the person with dementia.	Disease insight and motivation are factors for consideration when choosing a therapeutic approach to people with early stage dementia.	Primary User and Secondary Professional

References:

ALLEN, J. G. & FONAGY, P. 2006. *The Handbook of Mentalization-Based Treatment,* New York, Wiley.

BRAUN, V. & CLARKE, V. 2006. Using thematic analysis in psychology. *Qualitative research in psychology,* 3**,** 77-101.

CLARK, D. M. 2011. Implementing NICE guidelines for the psychological treatment of depression and anxiety disorders: The IAPT experience. *International review of psychiatry (Abingdon, England),* 23**,** 318-327.

GUBA, E. G. & LINCOLN, Y. S. (1989) *Fourth generation evaluation*. Newbury Park, CA: Sage.
HONOS-WEBB, L. & STILES, W. B. 1998. Reformulation of assimilation analysis in terms of voices. *Psychotherapy: Theory, Research, Practice, Training,* 35**,** 23.
HONOS-WEBB, L., SURKO, M., STILES, W. B. & GREENBERG, L. S. 1999. Assimilation of voices in psychotherapy: The case of Jan. *Journal of Counseling Psychology,* 46**,** 448.
KITWOOD, T. (2012). *Dementia reconsidered: The person comes first* 17th ed. NewYork, NY: Open University Press.
KNOBLOCH, L. M., ENDRES, L. M., STILES, W. B. & SILBERSCHATZ, G. 2001. Convergence and divergence of themes in successful psychotherapy: An assimilation analysis. *Psychotherapy: Theory, Research, Practice, Training,* 38**,** 31.
LOGSDON, R. G., GIBBONS, L. E., MCCURRY, S. M. & TERI, L. 1999. Quality of life in Alzheimer's disease: patient and caregiver reports. *Journal of Mental health and Aging,* 5**,** 21-32.
MELEIS, A. I. 2010. *Transitions theory middle-range and situation-specific theories in nursing research and practice / Afaf Ibraham Meleis, editor,* New York, Springer Pub.
REISBERG, B., FERRIS, S. H., DE LEON, M. J. & CROOK, T. 1982. The Global Deterioration Scale for assessment of primary degenerative dementia. *The American journal of psychiatry*.
RICHARD, N. 1994. Validation. Lichtungen im Nebel der Verwirrtheit finden [Validation therapy. Light in the fog of desorientation]. *Altenpflege,* 19**,** 196-199.
RITCHIE, J., LEWIS, J., NICHOLLS, C. M. & ORMSTON, R. 2013. *Qualitative research practice: A guide for social science students and researchers*, sage.
SEGAL, Z. V., WILLIAMS, J. M. G., & TEASDALE, J. D. (2002). Mindfulness-based cognitive therapy for depression: A new approach to preventing relapse. Guilford Press.
SMITH, J. & OSBORN, M. (2008) Interpretative phenomenological analysis. In: Smith JA (ed.) *Qualitative Psychology: A Practical Guide to Research Methods* (2nd edn). London: SAGE, pp. 53–80.
STILES, W. 1999. Signs and voices in psychotherapy. *Psychotherapy research,* 9**,** 1-21.
STILES, W. B. 2001. Assimilation of problematic experiences. *Psychotherapy: Theory, Research, Practice, Training,* 38**,** 462.
STILES, W. B., HONOS-WEBB, L. & LANI, J. A. 1999. Some functions of narrative in the assimilation of problematic experiences. *Journal of Clinical Psychology,* 55**,** 1213-1226.

List of Abbreviations:
AD, Alzheimer's Disease; BACP, The British Association for Counselling and Psychotherapy; CBT, Cognitive Behavioural Therapy; CORDIAL, Cognitive Rehabilitation and Cognitive-Behavioral Treatment for early dementia in Alzheimer disease; DT, Dignity Therapy; FG, Focus Group; IAPT, Improving Access to Psychological Therapy; IPA, Interpretative Phenomenological Analysis; IVA, Integrative Validation Therapy; LivDem, Living Well with Dementia; LTCF, Long-Term Care Facility; MADRS, Montgomery and Åsberg Depression Rating Scale; MAPED, Markers of Assimilation of Problematic Experiences of Dementia; MBCT, Mindfulness-Based Cognitive Therapy; MCI, Mild Cognitive Impairment; MHP, Mental Health Providers; MMC, Marte Meo Counselling; NHS, National Health Service; PATH, Problem Adaptation Therapy; PPI, Patient and Public Interest;, People with Dementia; QOL-AD, the Quality of Life in Alzheimer's Disease Scale; TAU, Treatment as Usual; VD, Vascular Dementia.
