# Supplementary material for: The role and impact of therapeutic counselling on the emotional experience of adults living with dementia: A systematic review
Source: Dementia (London). 2024 Apr 16;23(5):882–902. doi: 10.1177/14713012241233765 (PMC11163847; doi:10.1177/14713012241233765)
Supplement: Supplemental Material - The role and impact of therapeutic counselling on the emotional experience of adults living with dementia: A systematic review [file sj-pdf-6-dem-10.1177_14713012241233765.pdf]

Counselling Adults with Dementia: a review of on the role and impact of therapeutic counselling on the emotional experience of adults with dementia

TABLE 5: Characteristics Randomised Controlled Trials (RCT)

Author/ Date/ Country	Study Aim	Participants / Study Setting/ Intervention / Context	Study Design	Data Collection	Outcome Measures	Attrition	Results	Key Findings/ Recommendations	Outcome Type
Bailey, E.<br>2017<br>USA	To conduct a randomised controlled trial (RCT) of a nursing home multi-component intervention - question-asking reading QAR reminiscence and Cognitive-Behavioural Therapy (CBT) techniques - to reduce depressive symptoms in residents with dementia.	Nursing home residents (n=55) with dementia and depressive Symptoms (GDS>8) recruited from five privately owned, for-profit urban area nursing homes in Alabama.<br><br>Mean Age – 84yrs<br><br>(M5:F46)	RCT<br>Two groups<br><br>Intervention (n=26) Control (n=25)<br>Treatment as Usual (TAU)<br>Ongoing nursing home activities (e.g., singing, bible study, and bingo)	Baseline phase (n=2 weeks); Intervention phase (n=6 weeks)<br><br>Behavioural observation; self- and caregiver report; clinician interview	MMSE<br>CSDD<br>GDS<br>QOL-AD<br>AER<br>ABC	(n=4) 9%<br><br>One death.<br><br>Three failed to attend group sessions	(n=51)<br>Significant difference in depressive symptoms in TG between baseline (B) and post- intervention (PI) as compared to CG:<br>CSDD F(1, 49)- 13.43*; Cohen's d - 1.01<br>GDS F(1, 49)- 2.55; Cohen's d 0.40<br>QOL-AD F(1, 49) 2.41; Cohen's d 0.37<br>AER F(1, 495.53* Cohen's d 1.05<br>ABC<br>Expressive verbalization F(1, 49) - 20.77*; Cohen's d – 1.46;<br>Engagement with materials F(1, 49) - 51.92* ; Cohen's d – 2.68;<br>Laughter F(1, 49) - 10.47 ; Cohen's d – 1.00<br><br>QOL-AD remained stable in the	Residents receiving the QAR-Depression intervention showed significantly higher levels of expressive verbalisations, engagement with materials, and laughter, compared to control group.<br><br>Results suggest that the combined intervention positively impacted participants' levels of depression although few differences in resident behaviour outside of the group activities.<br><br>Small sample size with potential recruitment and intervention delivery bias.<br><br>The QAR structure may be suitable for older adults with cognitive impairment	User

Author/ Date/ Country	Study Aim	Participants / Study Setting/ Intervention / Context	Study Design	Data Collection	Outcome Measures	Attrition	Results	Key Findings/ Recommendations	Outcome Type
							experimental condition; a minor but non-significant decline in QOL was noticed for control condition participants.		
Jenewein, J. 2021 Switzerland	To determine the feasibility, acceptability and preliminary efficacy of Dignity Therapy (DT – a brief psychotherapeutic intervention to enhance dignity and reduce psychological burden, in patients with early stage dementia and in their families or close friends).	Early stage (very) mild dementia (CDR score between ≥0.5 and ≤1.5) and study partners (n=54) recruited from University Geriatric Outpatient Centre (Covid-19 disruption)<br><br>Age range 63-93 yrs Mean [SD] age = 79.6 yrs;<br><br>(M26: F28)	Wait list RCT<br><br>Four groups receiving two sessions of DT over 2 weeks<br><br>Immediate treatment (*n* = 28); Delayed treatment (*n* = 26) after 3 months waiting.	Baseline, post-treatment, 3-months follow-up) and group∗time interaction on outcome measures	Main outcome: Feasibility, acceptability and treatment satisfaction. Baseline: HADS; PDI; WHOQOL-BREF; FACIT-Sp-12<br><br>TG1 One week post-intervention – HADS & DTEQ<br><br>TG2 (15 weeks post baseline) – Repeat Baseline Qrre and DTEQ<br><br>T3 and T4 as first cohort	11.1% (n=6) (CI95%: 3.7–20.4%) TG1 (n=1) TG2 (n=5)<br><br>Treatment group 1 (n=28) with 1 drop-out during study; Treatment TG 2 (n=26) with 5 drop-outs.	Participant (with dementia) satisfaction after the intervention - 37.8 (CI95%: 35.7–39.8) and 40.4 (CI95%: 39.1–41.6) - near or above expected reference score (36.0)<br><br>Reduction of HADS in TG1 (Mean difference = −2.69, SE = 0.85, P = 0.003) and PDI scores (Mean difference = −6.56, SE = 1.63, P < 0.001) at 3-months and TG2 (Mean difference = −1.97, SE = 0.89, P = 0.031)<br><br>no significant differences in all other measures<br><br>no statistically	Treatment satisfaction high in patients and study partners - discussion of meaningful events and achievements as well as the generativity document most helpful parts. DT found to be feasible and acceptable to a sample of patients with early stage dementia and their family members. Underpowered. Short-term follow-up. Potential selection bias. Inclusion of only pre-screened patients with moderate psychological distress is needed to better ascertain the efficacy of this intervention among patients with early stage dementia.	User and Carer

Author/ Date/ Country	Study Aim	Participants / Study Setting/ Intervention / Context	Study Design	Data Collection	Outcome Measures	Attrition	Results	Key Findings/ Recommendations	Outcome Type
							significant group by time interaction effect on all outcome measures (e.g., HADS: F = 0.71; df = 2, 70.3; P = 0.50; PDI: F = 2.29; df = 1, 46.8; P = 0.14; Qol-social: F = 2.39; df = 1, 49.7; P = 0.13; FACIT-Sp-12: F = 1.41; df = 1, 49.0; P = 0.24)		
Kiosses, D.<br>2015<br>USA	To test the efficacy of 12-week Problem adaptation therapy (PATH – manualised guide) vs supportive therapy for cognitively impaired patients (ST-CI) in reducing depression and disability	Older adults (n=74) with major depression and cognitive impairment to the level of moderate dementia (DSM-IV).<br><br>Age range = 66 -95 years; Mean [SD] age = 80.90yrs;<br><br>(M23:F51)<br><br>Recruited	RCT<br><br>12 weekly sessions of Home-delivered PATH or Home-delivered ST-CI<br><br>Mixed-effects models for longitudinal data compared the efficacy of PATH with that of ST-CI in reducing depression	Baseline<br>4 weeks<br>8 weeks<br>12 weeks	MADRS; WHO-DASII	14.8%<br>(no detail on drop-out)	Participants in PATH - reduction in depression (Cohen d, 0.60;95% CI,0.13-1.06; treatment×time,F1,179 =8.03;P=.005) and disability (Cohen d, 0.67;95%CI,0.20-1.14; treatment×time,F1,169 =14.86;P=.001) than ST-CI participants during the12-week period (primary outcomes).<br><br>Compared with ST-CI, participants in PATH had greater decline in depression (43%) and	PATH more efficacious than ST-CI in reducing depression and disability. Reductions in depression and disability statistically and clinically significant.<br><br>Good acceptability of PATH by participants and caregivers – importance of carer participation stressed.<br><br>Home-delivered PATH may provide relief to a large group of depressed and cognitively impaired	User and Carer

Author/ Date/ Country	Study Aim	Participants / Study Setting/ Intervention / Context	Study Design	Data Collection	Outcome Measures	Attrition	Results	Key Findings/ Recommendations	Outcome Type
		through collaborating community agencies of Weill Cornell Institute of Geriatric Psychiatry.					disability (93%), respectively, at week 12.	older adults who have few treatment options	
Kiosses, D.<br>2015<br>USA	To examine the course of depression and suicidal ideation in a sub-group (from larger RCT) of 39 older adults with major depression and dementia during 12 weeks of home-delivered PATH and ST-CI.	Sub-group (n=39) of RCT participants (see above) with mild to moderate major depression and probable or definite dementia based on the DSM-IV criteria.<br><br>36% had mild suicidal ideation (CSDDSI = 1)	RCT<br><br>12 weekly sessions of Home-delivered PATH or Home-delivered ST-CI<br><br>Mixed-effects models for longitudinal data including time-trend parameter(s), treatment group, and time treatment interaction	Baseline<br>4 weeks<br>8 weeks<br>12 weeks	Total score and the Suicide Item (Item #16) of Cornell Scale for Depression in Dementia (CSDD)<br><br>WHODAS II<br><br>CSDD Anxiety item (Item #1)<br><br>Philadelphia Multiphasic Assessment Instrument Social Support Domain	33 (84.6%) completed the 12-week treatment.<br><br>Drop-out (n = 6), 3 receiving PATH and 3 ST-CI.	PATH participants had significantly greater reduction in depression than ST-CI participants over the 12-week period (treatment group by time interaction: $F[1,95.9] = 7.28$, $p = 0.0082$; Cohen's d: week 12:0.50. Caregiver involvement (yes vs no) and degree of dementia (mild versus moderate) were not significantly associated with the course of depression.<br><br>Participants with lower baseline anxiety scores had greater reduction in depression over 12 weeks of treatment (CSDD Anxiety:	Both treatments reduced depression. Baseline social support significant factor.<br>PATH participants had significantly greater reduction in depression than ST-CI participants (Cohen D = 0.50) but no difference in respect of suicidal ideation where both therapies offered similar benefit. Specific psychosocial treatment as opposed to generic supportive intervention recommended for suicidality.<br><br>Emphasises the use of well-validated, manualised, home-based psychotherapies in	User and Carer

Author/ Date/ Country	Study Aim	Participants / Study Setting/ Intervention / Context	Study Design	Data Collection	Outcome Measures	Attrition	Results	Key Findings/ Recommendations	Outcome Type
							F[1,32.2] = 5.45, p = 0.0260) in both treatments. There was no significant difference between PATH and ST-CI participants.	reducing depressive symptomology in depressed older adults with dementia.	
Koivisto (2016) Finland	To evaluate the effect of psychosocial intervention on the time of institutionalisation for patients with Alzheimer's disease (AD)	Early stage diagnosis of dementia rating of 0.5 (very mild) or 1.0 (mild) and their carers (CERAD-NB)<br><br>Patient-carer dyads (n=236) recruited from Department of Neurology, University of Eastern Finland.<br><br>TG (n=8) Control (n=152)<br><br>Intervention conducted in Memory clinics.	TG- Psychosocial rehabilitation (4 courses n=16 days) to enhance knowledge, reduce social isolation and caregiver distress, and to support functional ability and management everyday life situations. in first 2 years post diagnosis. Control: TAU Basic counselling at diagnosis.	Baseline 12, 24 and 36 month	Primary - Nursing home placement<br><br>Secondary - effect of early psychosocial intervention on AD progression using QoL-AD and VAS. Cognitive impairment (MMSE); Memory disorder severity CDR-SOB; ADCS-ADL; Behavioural disturbances (NPI); Mortality - 80% power and a significance level of 0.05 to detect a 20%	TG: 30% over 3 years (n=30) CG: 50% over 3 years (n=76)<br><br>Patients with AD who died (n=27): nine (11%) from the TG and 18 (12%) from the CG	No significant differences were found in nursing home placement. At 36months, 18 (21%) patients with AD from the intervention group and 24 (16%) from the control group lived in nursing homes. At the follow-up visits, the intervention group performed significantly worse in CERAD-NB, their ADCS-ADL scores were lower, and their CDR-SOB scores were higher compared with those of the control group. No significant differences were found in the NPI or QoL measures (15D, QoL-AD, and VAS) for patients with AD between the study groups during follow-up. no significant	The psychosocial intervention: did not delay nursing home placement in persons with AD; had no effect on health-related quality of life, disease progression, or AD-related symptoms of persons with AD; had no effect on caregiver distress, depression, or health-related quality of life. Results do not support the recommendations to automatically offer early, intensive course-based psychosocial intervention to all patients with very mild or mild AD and their caregivers in order to delay institutionalisation, AD progression, or caregiver distress.	

Author/ Date/ Country	Study Aim	Participants / Study Setting/ Intervention / Context	Study Design	Data Collection	Outcome Measures	Attrition	Results	Key Findings/ Recommendations	Outcome Type
					difference between the study groups after a 3-year follow-up.		effect of intervention on caregiver-related outcome measures	Need for tailored support/ follow-ups and counselling as required, especially in advanced stages of dementia.	
Marshall, A. 2015 England	To report a pilot study in which participants who had recently received a diagnosis of dementia were randomised to either a 10-week group intervention (Living Well with Dementia – LWWD) or a waiting-list control	People with dementia (n=58) diagnosed in previous 18/12 Recruited from NHS Memory Clinics in theSouth of England.<br><br>Of the 282 people attending memory clinics (MC), 131 did not meet eligibility criteria, 72 declined to participate and 19 were unavailable. MC staff trained to lead a 10-week	Pilot RCT Intervention or TAU (waitlist control)<br><br>Qualitative interviews - reported separately	Baseline (T1) 2 weeks post intervention completion (12 weeks) (T2) and 22 weeks (T3)	Primary - participant rated QoL-AD; Self-esteem; CSDD	Recruitmnt targets met < 10% attrition rate for TG	QoL-AD - improvement in TG compared to CG at Time 2 - 2.12 (95% CI =0.17, 4.42, effect size dD.46) which reduced to 0.30 at Time 3 follow-up (=2.09, 2.69). Improvement in self-esteem in TG compared to CG at Time 2 was 1.08 (=0.04, 2.20), which increased to 1.58 at Time 3 follow-up (=0.08, 3.25). Reduction in cognitive functioning in TG compared to CG of 1.34 at Time 2 (=2.88, 0.20) but deficit had largely disappeared at Time 3 follow-up,=0.45 (=2.07, 1.16).<br><br>No significant change in depression scores (CSDD) in either arm.	Need for effective interventions, which can be widely implemented and which focus on facilitating adjustment to a diagnosis. Some reluctance to attend group therapy suggests need for individualised options.	User

Author/ Date/ Country	Study Aim	Participants / Study Setting/ Intervention / Context	Study Design	Data Collection	Outcome Measures	Attrition	Results	Key Findings/ Recommendations	Outcome Type
		(75min) group programme (LWWD) conducted in NHS hospital or community sites<br><br>Seven programmes with 5-7 people with dementia in each<br><br>.					Non-significant trends towards improvements in both QoL-AD and self-esteem in the TG compared to the CG.		
Spector, A. 2015 England	To: (a) develop a cognitive-behavioural therapy (CBT) intervention manual and (b) assess the feasibility of the intervention through a single-blind, pilot RCT of CBT plus TAU versus TAU for	People with anxiety and mild to-moderate dementia (and carers) were randomly allocated to 10-session CBT (n=25) or TAU (n=25).<br><br>Age range = 66 -95 years;	Single-blind, multicentre, pilot RCT with two phases (Medical Research Council guidelines for developing a complex intervention and assessing feasibility)	Baseline, 15 weeks and 6 months	RAID; MMSE; QoL-AD; QCPR; HADS; CSDD; NPI	39 of the 50 dyads remained at 15 weeks and 38 at 6 months. Nine participants withdrew from the trial at first follow-up. Two participants were unable to be assessed at first follow-up but were	15 weeks:<br>RAID scale, anxiety lower in the TG group at 15 weeks (74.32, 95% CI 78.21 to 70.43) – Not Stat Sig.<br>Depression- CSDD significantly lower in the TG and remained so following adjustment (75.37, 95% CI 79.50 to 71.25).<br>No significant differences or notable trends in quality of life, cognition, anxiety and depression (measured	Adapted, manualised CBT (with flexible approach to delivery) acceptable to people with dementia and their family carer intervention was feasible for those with mild to moderate dementia (MMSE scores ranging from 25 to 16).	

Author/ Date/ Country	Study Aim	Participants / Study Setting/ Intervention / Context	Study Design	Data Collection	Outcome Measures	Attrition	Results	Key Findings/ Recommendations	Outcome Type
	people with dementia (supported by their carers). This included an assessment of acceptability, adherence, recruitment, retention and costs.	Mean [SD] age = 78 yrs;<br><br>(M20:F30)<br><br>Setting - NHS Hospital outpatient services				assessed at second follow-up. Another three withdrew from the trial at second follow-up	by the HADS) or the quality of caregiver–patient relationship from the carer or patient perspective<br><br>Cost per session TG was £114.36. Average session attendance per person - 8.8, Average total intervention cost per person was £1002<br><br>Data from this trial have been used to provide a power calculation for a full RCT, suggesting a minimum of 96 participants (48 in each group)		
Tonga, J. 2021 Norway	To evaluate the feasibility and effectiveness of CORDIAL, a psychosocial intervention consisting of CBT, cognitive rehabilitation	People with MCI (n=80) or early-stage dementia (n=116) recruited from five old age psychiatry and memory clinics at	Randomised controlled trial, based on a two-group (intervention and control - TAU), pre-/post-intervention design<br><br>Intervention	4 months (post treatment)<br><br>F.up - 10-months	Primary – MADRS<br><br>Secondary NPI; QoL-AD	Dropout rate from baseline to 4 months post intervention was 14% (27 of 198) and the dropout rate from 4 to 10 months post-intervention	Depressive symptoms assessed by MADRS were significantly more reduced in TG as compared to the CG ($p < 0.001$). The effect lasted 6 months post intervention. The average MADRS score at baseline was 7.6 = no clinical depression. No	Future studies could adopt a mixed-methods approach using qualitative methods to give insight into how people with dementia experience psychosocial interventions; Incorporation of an active control group;	User and Carer

Author/ Date/ Country	Study Aim	Participants / Study Setting/ Intervention / Context	Study Design	Data Collection	Outcome Measures	Attrition	Results	Key Findings/ Recommendations	Outcome Type
	and reminiscence to manage depressive symptoms for people with MCI or dementia	outpatients' hospitals in Norway<br><br>Intervention based on German CORDIAL study (Kurz et al., 2012), but adapted to Norwegian culture, post pilot study (Tonga et al., 2015; 2016)	group (n = 100); Control group -TAU (n=85)<br><br>TG - 11 individual weekly sessions of CORDIAL.<br><br>TAU- usual assessment appts plus eight sessions of CBT and ACT			was 20% (40 of 198)<br><br>Only 9.5% of TAU participants attended group therapy sessions; 57% of TAU participants did not participate in therapy or assessment appts.	significant differences between groups were found in neuropsychiatric symptoms or quality of life.	Longitudinal studies Points to the value of individualised therapy.	
Whitlatch , C. 2019 USA	To examine the efficacy of SHARE a six session, home-based, early stage, dyadic, psychosocial intervention for the person with dementia and his or her care partner.	People with a diagnosis of early stage dementia and care partner<br><br>SHARE dyads (n=84)<br><br>Control participants (n=44) received a single 90 min in-home	RCT – Waitlist control with option to participate after 6-months<br><br>Examination of key processes of treatment implementation and acceptability and the immediate rather than long-term effects SHARE	T1 – 1 week prior to SHARE or single Control intervention<br><br>T2 – SHARE participants post week 6<br><br>T2 -Control - 2 months post single session	MMSE; PCTS and care plan; SAM; DRS (adapted); CAS; EIDBS; D-QoLI; IADL; PADL Satisfaction with SHARE scale	Dyads attended on average 5.88 (SD = 2.28) sessions, with 78 (89%) dyads completing six sessions. Average session length was 78.39 min (SD= 17.34; range 40–195 min).	All SHARE dyads developed a balanced mutual care plan with significant decrease in the number of IADLs, PADLs, and socioemotional tasks assigned to the caregiver. Preference for PADL tasks to be completed by service providers and socioemotional tasks to be provided by family and friends Compared to the CG, participants in the	SHARE holds potential to address psychosocial needs of dyads in early stages of dementia. Using the dyadic protocol it was possible to create a plan of care that reflected both partners' care preferences and that balanced the provision of care tasks across family/friends, and service providers as well as the caregiver.	User and Carer

Author/ Date/ Country	Study Aim	Participants / Study Setting/ Intervention / Context	Study Design	Data Collection	Outcome Measures	Attrition	Results	Key Findings/ Recommendations	Outcome Type
		session led by a trained professional including information about dementia and available resource options<br><br>Dyads recruited through Alzheimer's Association chapters in northern Ohio.<br><br>Home-based intervention	might have on each member of the dyad			SHARE (n=76) completed T2 interviews CG (n=40)<br><br>12 (9.4%) lost to attrition at T2 (eight treatment dyads and four control dyads)	SHARE group increased the number of services they used during the intervention period. Caregivers in the CG reported an increase in emotional disruption during the intervention period, whilst this decreased for caregivers in the SHARE group.<br>No significant interactions were evident for reports of affect by either caregivers or the person with dementia. Caregivers and people with dementia in the SHARE group reported greater satisfaction than controls with their SHARE counsellor		

References:
KURZ, A., THÖNE-OTTO, A., CRAMER, B., EGERT, S., FRÖLICH, L., GERTZ, H.-J., KEHL, V., WAGENPFEIL, S. & WERHEID, K. 2012. CORDIAL: Cognitive Rehabilitation and Cognitive-behavioral Treatment for Early Dementia in Alzheimer Disease: A Multicenter, Randomized, Controlled Trial. *Alzheimer disease and associated disorders,* 26, 246-253.
STEVENS, A. B., CAMP, C. J., KING, C. A., BAILEY, E. H. & HSU, C. 1998. Effects of a staff implemented therapeutic group activity for adult day care clients. *Aging & mental health,* 2**,** 333-342.
STEVENS, A. B., KING, C. A. & CAMP, C. J. 1993. Improving prose memory and social interaction using question asking reading with adult day care clients. *Educational gerontology,* 19**,** 651-662

TONGA, J. B., KARLSOEEN, B. B., ARNEVIK, E. A., WERHEID, K., KORSNES, M. S. & ULSTEIN, I. D. (2016) Challenges With Manual-Based Multimodal Psychotherapy for People With Alzheimer's Disease: A Case Study. *American journal of Alzheimer's disease and other dementias.* 31 (4), 311–317.
TONGA, J. B., ARNEVIK, E. A., WERHEID, K. & ULSTEIN, I. D. (2015). Manual-based cognitive behavioural and cognitive rehabilitation therapy for young-onset dementia: a case report. *International Psychogeriatrics,* 28, 119–122.

List of Abbreviations:
ABC, Activity Behaviour Checklist; Ad, Alzheimer's disease; ADCS-ADL, Alzheimer's Disease Cooperative Study-Activities of Daily Living; AER, Activity enjoyment rating; CAS, Care-related Agreement Scale; CBT, Cognitive-Behavioural Therapy; CCCSI, Cornell Capacity to Consent Scale; CDR, Clinical Dementia Rating; CDR-SOB, Clinical Dementia Rating Sum of Boxes; CERAD-NB, Consortium to Establish a Registry for Alzheimer's Disease Neuropsychological Battery; CG, Controlled Group; CI, Confidence Interval; CORDIAL, Cognitive Rehabilitation and Cognitive Behavioural Treatment for Early Dementia programme; CSDD, Cornell Scale for Depression in Dementia; DSM-IV, Diagnostic and Statistical Manual of Mental Disorders; DQoL, the Dementia quality of life instrument; DT, Dignity Therapy; DTEQ, Dignity Therapy Evaluation Questionnaire; DRS, Dyadic Relationship Scale; EIDBS, Emotional-Intimacy Disruptive Behaviour Scale; FACIT-Sp-12, Functional Assessment of Chronic Illness Therapy Spiritual Wellbeing Scale; GDS, General Depression Scale; HADS, Hospital Anxiety and Depression Scale; IADL, Instrumental activities of daily living; MADRS, Montgomery-Asberg Depression Rating Scale; MCI, Mild Cognitive Impairment; MMSE, Mini-Mental State Examination; NHS, National Health Service; NPI, Neuro-psychiatric Inventory; PADL, Personal Activities of Daily Living; PATH, Problem adaptation therapy;PCTS, Preferences for Care Tasks Scale; PDI, Patient Dignity Inventory; QoL-AD, disease-specific quality of life Alzheimer's Disease; QCPR, Quality of Carer-Patient Relationship; QAR, Question- Asking, Reading; RAID, Rating Anxiety in Dementia Scale; RCT, Randomised Controlled Trial; SD, Standard Deviation (Mean); SAM, Service Availability Measure; SHARE, Support, health, activities, resources, and education; ST-CI, supportive therapy for cognitively impaired patients; TG, Treatment Group; TAU, Treatment as Usual; VAS, Visual Analogue Scale; WHOQOL-BREF, World Health Organisation Quality of Life; WHO-DASII, World Health Organization Disability Assessment Schedule II.
