# Supplementary material for: The role and impact of therapeutic counselling on the emotional experience of adults living with dementia: A systematic review
Source: Dementia (London). 2024 Apr 16;23(5):882–902. doi: 10.1177/14713012241233765 (PMC11163847; doi:10.1177/14713012241233765)
Supplement: Supplemental Material - The role and impact of therapeutic counselling on the emotional experience of adults living with dementia: A systematic review [file sj-pdf-7-dem-10.1177_14713012241233765.pdf]

Counselling Adults with Dementia: a review of on the role and impact of therapeutic counselling on the emotional experience of adults with dementia
TABLE 6: Characteristics - Other Studies

**Author/ year/ country**	**Study Aim**	**Participants/ Study Setting/ Intervention/ Context**	**Study Design**	**Data Collection/ Outcome Measures**	**Attrition**	**Results**	**Key Findings/ Recommendations**	**Outcome Type/ Perspective**
Alzubaidi, H. 2019 United Arab Emirates (UAE)	To evaluate community pharmacists' knowledge of AD and its management, counselling skills, and dispensing patterns when caring for people with AD and their caregivers in the UAE	Community pharmacists (n=325) from pharmacies (n=923) in three UAE cities: Dubai (n=140); Sharjah (n=170); and Ajman.	Cross-sectional survey using stratified random sampling	Questionnaires and interviews with community pharmacists<br><br>Alzheimer's Disease Knowledge Scale (ADKS); Alzheimer's Disease Pharmacotherapy Measure (ADPM)<br><br>Multivariate analysis using logistic regression model to predict counselling comprehensiveness	no data	Major shortcomings in pharmacist practices; history-taking, adherence assessment, and counselling provided by 2.2%, 9.3%, and 17.3%, respectively. Minority provided comprehensive counselling. Pharmacists did not provide structured patient-centred care for people with AD. Community pharmacists did not: provide adequate counselling; assess adherence-related issues appropriately; and, had deficient knowledge.	To develop patient-centred pharmacy-based services for Arabic-speaking communities, a multifaceted approach is required that goes beyond improving pharmacy workforce knowledge and communication skills to address broader sociocultural, legislative, and financial factors.	Secondary Professional
Berwig, M. 2020 Germany	To evaluate the feasibility of the Marte Meo® (MM) video-based counselling intervention in practice with primary carers of persons with bvFTD and assess	Home dwelling dyads - person with bvFTD and primary carer (n=5)<br><br>Four of the five persons with severe to very severe stage bvFTD.<br><br>MM individual and face to face	Non-randomised mixed-method feasibility study with quasi-experimental, single-arm, single-group, pre-	Quantitative interviews based on standardized questionnaires and videography at all three examination points in two sessions on two consecutive days at all three time points (t0, t1 after 2 weeks, and t2 after	None	MM counselling showed clinically very significant effects on the positive affect of the persons with bvFTD and the primary carers' stress experience due to BPSD.<br><br>Changes in the	MM counselling worked for people with bvFTD.<br><br>The acceptance of progressing dementia illness and the subsequent assumption of the carer role by a primary carer may be a central	Primary User

Author/ year/ country	Study Aim	Participants/ Study Setting/ Intervention/ Context	Study Design	Data Collection/ Outcome Measures	Attrition	Results	Key Findings/ Recommendations	Outcome Type/ Perspective
	the feasibility of a future confirmatory trial<br>Research Questions:<br>1 What are suitable outcomes and associated recording instruments for evaluating the effects of the intervention?<br>2 Are there descriptively shown effects in favour of the intervention and are these effects clinically significant?	counselling provided in 5 weekly sessions by a certified MM therapist experienced in using MM counselling with people with dementia and their primary carers.	post design with double pre-measurement and embedded qualitative change evaluation.	6 weeks).<br><br>Positive and negative affect; behavioural and psychological symptoms in dementia (BPSD); interpersonal abilities of the person with dementia; the sensitivity and distress of the primary carers due to BPSD; manageability of BPSD; the personal goal attainment by means of MM counselling; and, the quality of the dyadic relationships.		quality of the dyadic relationships as assessed from primary carer perspective described as clinically significant.<br><br>Measures deemed suitable to describe the effect of MM counselling in a future confirmatory trial.	promoting factor for increasing the benefits of MM counselling.<br><br>Recruitment process as a whole was very laborious and time consuming.<br><br>Important to invite feedback from PlwD<br><br>Additional follow-up sessions after a period of several weeks	
Bhattacharjee, S.<br>2017<br>USA	Prevalence, Patterns, and Predictors of Depression Treatment among Community-Dwelling Elderly Individuals with Dementia in the United States	Community dwelling elderly (age ≥65 years) individuals who 1) had dementia, 2) were alive during the calendar year, and 3) had received depression treatment, identified by antidepressant medication with or without psychotherapy	Retrospective cross sectional survey Multiple years of Medical Expenditure Panel Survey (2002, 2004, 2006, 2008,	Dependent variable - depression treatment, identified by antidepressant medication with or without psychotherapy use.<br><br>χ2 tests and analysis of variance to establish difference between groups. Multinomial logistic regression –	n/a	Co-occurring depression prevalence in 22% of sample [95% CI]: 18.44%–24.74%). Nearly 88% reported receipt of depression treatment 95%CI: 84.9%–90.8%. Antidepressants only and combination therapy (antidepressant with psychotherapy) was	Race/ethnic disparity in depression treatment.<br><br>Need to explore comparative effectiveness of antidepressant alone vs combination therapy of antidepressants and psychotherapy among elderly individuals with co-	Secondary<br>Professional

Author/ year/ country	Study Aim	Participants/ Study Setting/ Intervention/ Context	Study Design	Data Collection/ Outcome Measures	Attrition	Results	Key Findings/ Recommendations	Outcome Type/ Perspective
			2010, and 2012) data.	individual factors associated with depression treatment		reported by 75% (95% CI: 69.8%–80.0%) and 13% (95% CI: 8.9%–17.1%), respectively. Several subgroup differences, e.g. age re reporting the use of depression treatment.	occurring dementia and depression.	
Blair, A. C. 2016 Australia	To evaluate the clinical effectiveness and practical viability of group psychotherapy for older frail residents of an aged-care facility with anxiety and/or depressive symptoms	Residents with multiple physical comorbidities and loss of physical independence (n=6);<br><br>Person with dementia (n=1)<br><br>Mean age - 88 years<br><br>8 weekly sessions of group psychotherapy loosely based on cognitive behaviour therapy (CBT) principles in low-level residential care facility	Pilot study with pre-post, test measures.	Questionnaires; weekly verbal feedback from participants; Researcher journal.<br><br>Beck Depression Inventory II (BDI-II); Beck Hopelessness Scale (BHS); Geriatric Anxiety Inventory (GAI); 21-item Depression Anxiety Stress Scale (DASS 21).	n=2	Variable across participants. No statisitical analysis due to low numbers. No-one completed the minimal homework assignments though they were supported by reminders and audio and written material; participants were unable to articulate any goals despite repeated discussions and guidance.<br>Positive engagement with group process reported.	1 Older adults in residential care in Australia have very limited access to psychological services despite depression and anxiety rates of up to 50% for this population. 2 Very frail, very elderly residents with multiple physical comorbidities can participate in the process of group therapy.<br>3 There are significant logistical and clinical issues to be addressed when working with groups in residential aged-care facilities.<br>4 Important to tailor content to the specific needs of participants	
Cornelis,	To determine	Person with dementia	Retrospecti	Everyday functioning	Attrition	For persons with	Not clear how much	Primary

Author/ year/ country	Study Aim	Participants/ Study Setting/ Intervention/ Context	Study Design	Data Collection/ Outcome Measures	Attrition	Results	Key Findings/ Recommendations	Outcome Type/ Perspective
E.<br>2018<br>Belgium	whether the multicomponent rehabilitation programme (MRP) of a memory clinic had positive outcomes on ameliorating everyday functioning, quality of life, mood and behavioural disturbances of persons with dementia and reducing the distress and burden of caregivers	and their caregivers.<br><br>Participation in the Universitair Ziekenhuis Brussels memory clinic MRP. Maximum duration of 1 year with 25 x 1-hour counselling sessions	ve pre-test–post-test study without control group.	in basic, instrumental and advanced activities of daily living, cognition, mood, emotional and behavioural disturbances, quality of life and caregiver burden.	(n=8)<br><br>Over 52 wks, average attendance at 15 sessions (max 25) of which 6 were psychological.<br><br>50% of those who left refused to continue.	dementia (n=22), participating in the programme did not improve everyday functioning, cognition or mood. The GDS-15 (Z=–2.5, p=0.001, 95% CI (.016–.021)) showed significantly more limitations and declines at the end of the programme. Quality of life improved significantly (Z=–2.7, p=0.006, 95% CI (.003–.005)) and stabilised mood, emotional and behavioural disturbances for 60% or more of them. For caregivers (n=22), the mild to moderate burden of care remained stable or got better for 63.6% of the caregivers.	therapeutic counselling was included in the programme - ? dependent on user preferences but appeared that more focus was placed on functional and cognitive aspects of care. Small sample size and effect sizes of the outcome measurements small thus cannot draw firm conclusions regarding clinical implications of the results	User (self report)
Cheston, R.<br>2015<br>England	To describe the use of the ‘‘Living Well with Dementia’’ or LivDem model of group support for people affected by dementia	Person with dementia (n=5) diagnosed within previous 18 months and Carers (n=4)<br><br>Recruited from primary care (n=3) and local Memory clinic (n=5).	Feasibility Mixed Methods	1st and 10th sessions Telephone interviews (n=3) 1 week post intervention<br><br>Focus group with LivDem facilitators<br><br>QoL–AD	Person with dementia (n=2)<br>Carers (n=1)<br>85% dyad attendance over 10 sessions	QoL-AD ratings improved for two of the three participants over the course of the intervention. One participant found it unhelpful. Therapists described important changes in the way participants spoke about their	LivDem intervention helpful for two of the three participants with dementia. Carers appeared to gain the most benefit. Therapist need for Dementia knowledge/experience and supervision	Primary User (Self-report) and Professional

Author/ year/ country	Study Aim	Participants/ Study Setting/ Intervention/ Context	Study Design	Data Collection/ Outcome Measures	Attrition	Results	Key Findings/ Recommendations	Outcome Type/ Perspective
	within a Primary Care setting	LivDem group support - 10 week model				illness.		
Churcher Clarke, A. 2017 England	To investigate the feasibility and the potential benefits of an adapted mindfulness programme for people with mild to moderate dementia living in care homes	People with mild to moderate dementia<br><br>Intervention (n=20) Control Group treatment as usual [TAU] (n=11)	Feasibility single-blind, multicentre randomised controlled pilot study<br><br>Adapted 10-session mindfulness programme (1Hr x 2/week over 5 weeks) plus Tau conducted in four care homes in England	Baseline and 1 week post-intervention<br><br>Cornell Scale for Depression in Dementia (CSDD); Rating Anxiety in Dementia Scale (RAID); QoL-AD Mini Mental State Examination (MMSE); The Perceived Stress Scale (PSS-13)	Control group (n=3) did not complete f.up measures.<br><br>Mean attendance -8.15 sessions (SD = 2.46, range 1–10). Reasons for non-attendance - being unwell or asleep	Improvement in quality of life in the intervention group compared to controls ($p = 0.05$). No significant changes in other outcomes.	The intervention was feasible in terms of recruitment, retention, attrition and acceptability and was associated with significant positive changes in quality of life. Fully powered randomised controlled trial needed.	Primary User (self-report)
Collins, R. 2018 England	To evaluate the effectiveness of a Compassion-Focused Therapy (CFT) group on reducing anxiety, depression, and respiratory rate (RR) in	Community dwelling people with dementia invited from Memory Assessment and Treatment Service (MATS) with spousal caregiver (Caucasian in heterosexual relationship).<br><br>Dyads (n=34) Person with dementia (M:20,	Feasibility using Pre-post tests analysing repeated measures using t-tests and Reliability Change Index	Questionnaires<br><br>Outcome data from 10 CFT groups between 2013 to 2017.<br><br>HADS (from 56 participants (n=41) (21 spouses and 20 Person with dementia) completions	64 participants completed the intervention.<br><br>Attrition rate of 6%.	57% of people with dementia with borderline to abnormal baseline scores showed clinically significant improvement in anxiety and depression. For spouses, 80% showed clinically significant improvement in	CFT appears effective in improving QoL and depression in people with dementia and reducing RR in people with dementia and spouses.<br><br>CFT reduces anxiety and depression in most people with	Primary User (self report)

Author/ year/ country	Study Aim	Participants/ Study Setting/ Intervention/ Context	Study Design	Data Collection/ Outcome Measures	Attrition	Results	Key Findings/ Recommendations	Outcome Type/ Perspective
	people with dementia (People with dementia) and their spouses, and improving the quality of life (QoL) of Person with dementia	F:12)<br><br>57-85yrs<br><br>AD (47%); Mixed Vascular and AD (28%); Vascular Dementia 12.5%); Dementia with Lewy Bodies (9%); Parkinson's with with Lewy Body dementia (3%)<br>CFT groups (n=10) based on Gilbert's (2009) model<br>six weekly two hour CFT sessions		Respiratory rate (15 People with dementia and 13 spouses)<br>QOL-AD (only in last 2 groups) from potential 12 dyads (n=24) 17 participants (8 dyads and 1 spouse) completed feedback forms; rating the helpfulness of the session on a bar-chart scale from 0 (least helpful) to 10 (most helpful)- scores 7 and above increased over time. Documentation of verbal feedback from participants.		depression and 50% in anxiety. RR reduced for people with dementia and spouses with large and medium effects respectively. QoL of people with dementia improved with a large effect.	dementia and spouses with borderline to abnormal symptoms	
Craig, C. 2018 England	To develop a therapeutic intervention based on CFT for people with dementia with depression and/or anxiety, and to assess its feasibility, acceptability, and utility within this	Person with dementia (n=7)<br>And depression (>10 on the Cornell Scale for depression in dementia; CSDD); and/or anxiety (≥11 on the Rating Anxiety in Dementia; RAID).<br><br>Age 53-88yrs (M1:F6)<br><br>Recruited from three	Mixed methods Case Series<br><br>Measures at 3 Time-points: pre-interventio n; mid-point; post-interventio n	Face-to-face interviews Sessions.<br><br>CSSD<br>RAID<br>Session rating form (SRF) likert scale completed for 48 out of 66 (73%)<br>Post-therapy change interviews semi-structured interview	Five participants completed course. Two completed eight sessions<br>1 died but data included	No adverse events.<br><br>Improvements in measures of mood, anxiety, and self-compassion.<br><br>Not clinically significant<br><br><br>Small sample size and no follow-up	CFT was mostly well received by people with dementia.<br>Those with mild-moderate cognitive impairment may be able to engage in a CFT intervention.<br>CFT may increase self-compassion and improve low mood and anxiety symptoms in people	Primary User

Author/ year/ country	Study Aim	Participants/ Study Setting/ Intervention/ Context	Study Design	Data Collection/ Outcome Measures	Attrition	Results	Key Findings/ Recommendations	Outcome Type/ Perspective
	population.	memory services and one dementia charity organisation.<br><br>Ten-week intervention delivered by 3 clinical psychologists and 1 trainee clinical psychologist, all trained in CFT.		(Person with dementia: n=4), designed for the study and based upon Elliot, Slatick, and Urman's25 change interview		data.	living with dementia. The intervention needs flexibility with manual use and may require an increased number of sessions.<br><br>A pilot trial with a larger sample is recommended.	
Garcia-Alberca, J. 2017 Spain	Describe a multimodal approach to treatment derived from cognitive–behavioural interventions to treat clinically significant depression in a patient with AD and clinically significant depression.	75-year-old male diagnosed with mild dementia and spouse.<br><br>Recruited from the Dementia Unit of the Andalusian Institute for Neuroscience and Behaviour, Málaga.<br><br>CBT for depression in AD (CBT-DAD), nine weekly (45–60 min) sessions then six biweekly f,up sessions over 3 months. include pleasant events therapy, problem-solving therapy, exercise, and education, as well as cognitive restructuring for the caregiver.	Case Study Report	CBI; CSDD; CSI,; NPI-DES; NPI-DIS;. CSDD.	None	Decrease in depression scores.<br><br>Caregiver distress and caregiver burden decreased.<br><br>Caregiver engagement coping strategies increased post-treatment and disengagement scores decreased. 3-month follow-up assessment showed rating scale scores remained at post-treatment levels.	Treatment benefit points to decreased sadness, improved mood and increased participation in pleasant activities.<br><br>CBT-DAD may be an effective intervention for reducing depression in patients with mild AD. Caregiver participation essential.	Primary User/Carer
Jennings,	To measure	Community-dwelling	Retrospecti	Aggregate quality of	Fifteen	Individuals were	Comprehensive	Secondary

Author/ year/ country	Study Aim	Participants/ Study Setting/ Intervention/ Context	Study Design	Data Collection/ Outcome Measures	Attrition	Results	Key Findings/ Recommendations	Outcome Type/ Perspective
L.<br>2016<br>USA	the quality of dementia care provided by nurse practitioner dementia care managers (DCM) through the Health System created the University of California at Los Angeles (UCLA) Alzheimer's and Dementia Care (ADC) Program	adults with dementia (n=797) referred to UCLA ADC program over 2-years.<br><br>Quality improvement program that uses a co-management model with DCMs working with primary care physicians and community-based organisations to provide comprehensive dementia care	ve analysis of care records using descriptive statistics.<br><br>Quality Indicators (QIs) abstracted from DCM notes over a 3-month period from date of initial assessmen t.	care for the UCLA ADC cohort, calculated as the total number of recommended care processes received divided by the total number of eligible QIs (n=17); Assessing Care of Vulnerable Elders (ACOVE-3); Physician Consortium for Performance Improvement Qis.<br><br>Secondary outcomes - aggregate quality of care in three domains of dementia care: Assessment and Screening (7 QIs), Treatment (6 QIs), and Counselling (4 QIs).	people (2%) died during the 3-month abstraction window, and 28 (4%) had documentat ion indicating life expectancy less than 6 months or had been referred to hospice.	eligible for 9,895 QIs, of which 92% were passed. Overall pass rates of DCMs were similar (90–96%).<br>All counselling and assessment QIs had pass rates greater than 80%, with most exceeding 90%. Wider variation in adherence among QIs addressing treatments for dementia, which patient-specific criteria triggered, ranging from 27% for discontinuation of medications associated with mental status changes to 86% for discussion about acetylcholinesterase inhibitors.	dementia care co-management with a nurse practitioner can result in high quality of care for dementia, especially for assessment, screening, and counselling. The effect on treatment QIs is more variable but higher than previous reports of physician-provided dementia care.	Professional
Jo, H.<br>2016<br>Korea	To apply the "Happy Together" integrated cognitive behaviour programme for elderly people with dementia who are living at home	Elderly individuals and their caregivers (n=46);<br>Experimental Group Dyad (n=24)<br>Control Group Dyad (n=22)<br><br>Age <65 yrs<br><br>Theoretical framework based on CBT	Quasi-experiment al study using non-equivalent control group pretest–posttest design.	Frenchay Activities Index;<br>Problematic behaviours of elderly people with dementia (Ma and Kim 1994);<br>The Life Satisfaction Index-A; Zarit Burden Interview;<br><br>SPSS version 21.0 program to calculate χ2 tests, Fisher's	No data	No significant difference (t=1.39,P=0.172) in the problematic behaviours of elders with dementia.<br>Significant difference (t=-2.38, P=0.022) in their IADL;<br><br>Caregiver burden significantly decreased in the	Eight sessions deemed insufficient to markedly improve problematic behaviours	Primary User (self report)

Author/ year/ country	Study Aim	Participants/ Study Setting/ Intervention/ Context	Study Design	Data Collection/ Outcome Measures	Attrition	Results	Key Findings/ Recommendations	Outcome Type/ Perspective
		8 weekly (120min) sessions held in Mental Health Centres<br>3 components: warm up & hands & feet exercise (10minutes), performing the CBT strategy (95minutes), and concept memory training (15minutes)		exact tests, and independent t-tests		experimental group (t=3.43, P=0.002);<br><br>No significant difference between the two groups in terms of QOL (t=0.74,P=0.466)		
Johnston, B.<br>2016<br>Scotland	To assess the feasibility, acceptability and potential effectiveness of modified Dignity Therapy (DT) to improve the quality of life and reduce psychological and spiritual distress in older individuals with dementia	Participants (n=27): People with Early Stage Dementia (n=7); family members (n=7); stakeholder participants (n=7); and, focus group members (n=6) (who were joined by 2 existing participants (n=8 -5 people with ESD and 3 family members)<br>referred to the post-diagnostic dementia service by a specialist clinician in an east of Scotland community setting.<br><br>Dignity Therapy - short psychotherapeutic intervention that uses a trained therapist to take the person with dementia through a	Mixed method feasibility	Herth Hope Index (HHI)<br>Patient Dignity Inventory (PDI)<br>Perceived Quality of Life, and Satisfaction with Quality of Life Ratings<br><br>Data (transcripts) from the DT documents, post DT interviews, focus group discussions and stakeholder interviews were thematically analysed using Framework Analysis (Ritchie et al., 2013).	No data	Outcome measures show potential to indicate changes in quality of life and psychological and spiritual distress as a result of Dignity Therapy	Indicates that method and outcome measures proposed were appropriate and acceptable to this group of participants, and would transfer well to a larger scale study. Participants were ‘more positive regarding openly exploring their inner thoughts and feelings surrounding their life events’. Participants also reported feeling relieved about having the Dignity Therapy document as a legacy to pass to their family.	Primary Professional and User

Author/ year/ country	Study Aim	Participants/ Study Setting/ Intervention/ Context	Study Design	Data Collection/ Outcome Measures	Attrition	Results	Key Findings/ Recommendations	Outcome Type/ Perspective
		guided interview process, producing a ‘generativity’ document that creates a lasting, written legacy during a time when they are still able to communicate well						

Kovach C. 2018 USA	To determine the feasibility of older adults with multiple chronic conditions and a subset with cognitive impairment participating in a mindfulness intervention, and (b) compare short- and long-term changes in agitation, aff ect, sleep, stress, and interoception when participating in a mindfulness intervention and therapeutic cognitive activity intervention.	Individuals with multiple Chronic conditions, including cognitive impairment (n=36)<br><br>People with moderate and severe stages of dementia (n=20)<br><br>Average age- 87 years (SD = 10.2 years; range = 56 to 98 years).<br><br>Eight groups run over a 16-	Controlled crossover Repeated measures experimental design Participants randomised into a sequence of two study conditions: Present in the Now (PIN) and a cognitive therapeutic activity (COG) for 4 weeks each with a 2-week washout period	Baseline and 1-week post intervention Cohen-Mansfield Agitation Inventory Observed Emotion Rating Scale Arousal States in Dementia Scale; Salivary cortisol assay; Actigraphy; Discomfort–Dementia of the Alzheimer's Type scale; Communication of need	29 (81%) of 36 PIN participants attended seven or more of the 11 sessions offered to each group and 11 (28%) participants attended all sessions. In the COG group, 18 (50%) participants attended seven or more sessions.	Statistically significant short-term changes in agitation between the PIN and COG activities (F [1, 28] = 6.89, p = 0.014) Long-term agitation decreased in the COG condition from a mean of 67.16 (SD = 33.23) at baseline to 55.47 (SD = 35.20) at 1-week posttest (t = 1.84, p = 0.038, one-tailed). Anger and anxiety/fear decreased during and post the PIN intervention, (anger F [1, 31] = 2.94, p = 0.030, one-tailed; anxiety/fear F [1, 31] = 5.94, p = 0.004). Pleasure increased during and post (20mins) the PIN intervention (F [1, 31] = 2.40, p = 0.046,one tailed). No change to sadness and alertness in either PIN or COG. Night-time sleep did not improve. Discomfort scores decreased for the PIN group by 193 points and increased by 19 points after the COG activity (F [1, 27] = 32.63, p < 0.001, partial eta squared = 0.547. No long-term changes in outcomes.	Mindfulness is feasible for older adults with multiple chronic conditions and cognitive impairment. The intervention can be conducted in a residential setting, and is associated with positive benefits.	Primary Professional

Orsulic-Jeras, S. 2019 USA	To determine the acceptability of SHARE (Support, Health, Activities, Resources, and Education) programme	People with early stage dementia (ESD) and family caregivers (CG) – Forty community dwelling, English-speaking dyads able to read printed material.<br><br>Recruited over 20-months from various social service agencies in Northeast Ohio: 160-bed nursing home (n=12) and 96-unit apartment that contained independent (n=12)and assisted living (n=5) (≥4 weeks in residence)<br><br>PIN intervention (n= 4 groups) -MBI with three Components: attentional skill exercises; body awareness activities; compassion meditation<br>COG intervention (n= 4 groups) - cognitive activities that stimulate memory and thinking, e.g. wordplay, mental aerobics, trivia.	Feasibility Interviews at Time 1 (baseline) and Time 2 (2wks post intervention)<br><br>11 x (45 mins) sessions of PIN and COG run mid- to late morning on 2 days in week 1 and 3 days per week for the next 3 weeks	Care Values Scale (CVS);<br>Preferences for Care Tasks Scale; (PCTS);<br>Likert Scale (1-5)	26 dyads (65%) completed the 7 SHARE sessions whilst 14 (35%) dyads completed in a fewer no.	SHARE Counselor's level of skill, helpfulness, and friendliness highly rated, as was the usefulness of session information and materials (Avge Likert Scale score of 3.03 to 3.77)<br>SHARE Counselors successfully built sufficient rapport with care partners, as evidenced by 100% of CGs and 97% of people with dementia.<br>Most CGs (97%) and people with dementias (94%) felt the SHARE Program gave them an opportunity to express their thoughts and feelings.<br>82% of CGs and 81% of people with dementias felt that their relationship with each other had improved. the majority of care dyads strongly agreed they understood the illness better (CG = 97%; people with dementia = 90%), felt more control over the care situation (CG ¼ 90%; people with dementia = 88%), felt better prepared for what lies ahead (CG = 95%; people with dementia = 94%), and were ultimately more	Programme well received overall highlighting the importance of early planning and decision making so that people with dementia can voice their care values and preferences for future care. More CG responses than people with dementia and some dissatisfaction/emotional distress noted by people with dementia	Primary User (self report and participant comments)

						confident making care decisions (CG ¼ 92%; people with dementia =90%)		
Paller, S. 2015 USA	To implement a mindfulness programme for patients and caregivers that could be a model for future interventions	Person with dementia/MCI or other form of memory impairment (n=17) Caregivers (n=20)<br><br>Recruited through the University Alzheimer's Disease Centre via local advertisements and word of mouth.<br><br>Eight group mindfulness sessions orientated to the needs of patients with memory loss due to a terminal illness and to the needs of their caregivers	Pre-post test	Quality of life in AD (QoL-AD); Geriatric Depression Scale (GDS); Pittsburg Sleep Quality Inventory (PSQI); Beck Anxiety Inventory (BAI); Trail-Making Tests A and B; Battery for the Assessment of Neuropsychological Status (RBANS); Revised Memory Problem and Behaviour Checklist (RMPBC); Short Form Health Survey (SF-36); Activities of Daily Living Questionnaire (ADLQ).	No data	Pre–post analyses revealed several benefits, including increased quality-of-life ratings, fewer depressive symptoms, and better subjective sleep quality. Significant scores recorded only in GDS average scores decreased 1.4 points (SE = .7) (F1,35 - 4.16, P = .049) and QoL-AD with an average increase of 1.8 points (standard error [SE] = 0.5) from the initial session to the subsequent session (main effect of session, F1,33 ¼ 15.06, P < .001). 84% of the participants felt they benefited from the program, 89% said they would continue with the mindfulness techniques, and 89% said they would recommend the program to someone in their situation	Participants indicated that they were grateful for the opportunity to learn to apply mindfulness skills and that they would recommend the programme to others.<br><br>Mindfulness training can be beneficial for patients and their caregivers and can be delivered at low cost to combined groups	Primary User and carers
Plunger, P. 2019 Austria	To train community pharmacy staff in dementia care, and to establish	Community pharmacies (n=40) - 18 from two Austrian provinces (Vienna and Lower Austria), and a	Health promotion approach based on organisation al change	Semi-structured interviews, focus groups and document analysis. Survey data.	None	Programme development and implementation: Pharmacy staff identified lacking communication skills	For further attention: specifics of the community pharmacy setting, motives for change, the	Secondary Professional

	community pharmacies as easily accessible contact points for caregivers, people with dementia and the general population.	follow-up project (March 2016–August 2017) with 22 community pharmacies from the city of Salzburg.<br><br>The ‘Dementia-friendly Pharmacy’ programme encompassed the pilot project (May 2013–December 2015) with The two projects were both carried out in three phases: needs assessment; training phase; and, realisation of small-scale initiatives.	and participatory health research (Implementation Science – Mixed methods) –2-fold aim of exploring social reality while at the same time creating positive social change.	Thematic analysis<br>Reflexivity<br>Descriptive statistics		with disoriented people as the most relevant issue.<br>Relevance of dementia care established and fields of activity and their specific meaning for community pharmacy identified: training counselling knowledge and skills, strengthening professional networks and consolidating knowledge on medication<br>stigma of dementia addressed and logo created to foster visibility<br>ongoing peer-support and training.<br>Self-rated competency in counselling and on network contacts support the notion of effectiveness.	impact of the health and social care system as well as the local community environment as meta-setting(s), and involving caregivers and people with dementia.<br>Need to balance the professional orientation with the business mandate in community pharmacy and to tackle the still dominant product-focus, with counselling appearing as an appreciated but still auxiliary service.<br>To be implemented and sustained, counselling strategies developed had to fit with the existing organisational structure, culture and professional practice.<br>Importance of partnership building to refer on. Need for user involvement in service	

							development	
Skov, S. 2022 Denmark	To investigate the acceptability and potential impact of a multicomponent intervention comprising physical exercise, cognitive stimulation therapy (CST), psychoeducation and counselling for people with early-stage dementia.	People with early stage dementia (n = 44) living in Copenhagen.<br><br>Recruited from public health and social authorities, e.g. memory clinics, general practitioners, home-care staff and dementia consultants<br><br>Qualitative data participants (n=14) –mostly male<br><br>15-week multicomponent group-based intervention delivered at the Centre for Dementia, Copenhagen	Mixed-methods pilot design	Combination of interviews, observations and tests of cognitive and physical functioning: MMSE; Quality of Life (QoL-AD); Chair stand test, 10-m walk test single- and dual-task) and an interviewer-assisted questionnaire on quality of life to:<br>(1) investigate acceptability of the intervention, including whether people with dementia and their caregivers found the intervention meaningful;<br>(2) to explore and assess changes in participants' physical and cognitive functioning and quality of life	Dropped out programme (n=5); Lost to f.up (n=8)<br><br>Average of 25.5 (out of 30) attendances per participant	No significant differences between baseline and post-intervention measures of cognitive and physical functioning or in quality of life. Participants did not find purpose or meaning in CST component. Much larger number of sessions for exercise (n=30) and CST (n=27) in comparison to psychoeducation (n=3) and counselling (n=3). Qualitative data revealed that participants found aspects of the intervention beneficial and that it had a positive influence on their physical and social well-being. Interaction and support from peers and staff members was considered important and rewarding.	Prolonged and sustainable interventions are needed for people with dementia to maintain personal and social benefits	Primary User and Carer
Tonga, J. 2016 Norway	To explore if a multimodal psychotherapy approach that combines CBT and CR could be used to manage depressive symptoms in patients with young-onset	63-year-old single woman living independently in own apartment, diagnosed with early-onset Alzheimer's disease at Memory Clinic of Oslo University Hospital, Norway.<br><br>Manualised	Case Report<br><br>Pre- Post-<br>6 month f.up	MADRS<br>Pre 15/60<br>Post 3/60<br>6mnth 4/60<br><br>MMSE<br>pre 20/30<br>post 29/30<br>6mnth 30/30<br><br>HADS<br>Pre 14/42	None	User satisfaction with the treatment.<br>Improved mood.<br>The caregiver stated that written home assignments especially useful to support the treatment process.<br>At 6-month f.-up participant reported being less depressed and was still	Further studies needed to examine the effect of psychotherapy on depression in a larger patient sample of YOD.	Primary User Self report

	dementia (YOD)	treatment (n=11 structured sessions with homework) based on the CORDIAL study (CR and CBT for people with Alzheimer's disease) (Kurz et al., 2012).		Post 8/42<br>6mnth 12/42		participating in the activities. No longer using calendar.		

References

GILBERT, P. 2009. Introducing compassion-focused therapy. Advances in psychiatric treatment : the Royal College of Psychiatrists' journal of continuing professional development, 15**,** 199-208.

KURZ, A., THÖNE-OTTO, A., CRAMER, B., EGERT, S., FRÖLICH, L., GERTZ, H.-J., KEHL, V., WAGENPFEIL, S. & WERHEID, K. 2012. CORDIAL: Cognitive Rehabilitation and Cognitive-behavioral Treatment for Early Dementia in Alzheimer Disease: A Multicenter, Randomized, Controlled Trial. Alzheimer disease and associated disorders, 26**,** 246-253.

MA, J. & KIM, C. 1994. Study on the effect of the behavior problems for the demented elderly upon the stress among family members. J Korean Soc Health Educ, 12**,** 83–109.

RITCHIE, J., LEWIS, J., NICHOLLS, C. M. & ORMSTON, R. 2013. Qualitative research practice: A guide for social science students and researchers, Sage

List of Abbreviations:

AADL-CDI: Advanced Activities of Daily Living – Cognitive Disability Index; AADL-DI, Advanced Activities of Daily Living – Disability Index; AADL-PDI: Advanced Activities of Daily Living – Physical Disability Index; ACOVE, Assessing Care of Vulnerable Elders; AD, Alzheimer's Disease; ADC, Alzheimer's and Dementia Care; ADLQ, Activities of Daily Living Questionnaire; ADKS, Alzheimer's Disease Knowledge Scale; ADPM, Alzheimer's Disease Pharmacotherapy Measure; BADL (Katz), Basic Activities of Daily Living according to the Katz Scale; BAI, Beck Anxiety Inventory; BDI-II, Beck Depression Inventory II; BHS, Beck Hopelessness Scale; BPSD, Behavioural and Psychological Symptoms in Dementia; bvFTD, behavioural variant Frontotemporal Fementia; CBT, Cognitive Behavioural Therapy; CBT-DAD, Cognitive-Behavioural Therapy for Depression in Alzheimer's Disease; CFT, Compassion-Focused Therapy; CG, Caregivers; CORDIAL, Cognitive Rehabilitation and Cognitive-Behavioral Treatment for Early Dementia in Alzheimer Disease; COG, Cognitive Therapeutic Activity; CSDD, Cornell Scale for Depression in Dementia; CSI, Coping Strategies Inventory; CST, Cognitive Stimulation Therapy; CVI, Caregiver Burden Interview; CVS, Care Values Scale; CR, Cognitive Rehabilitation; DASS 21, 21-item Depression Anxiety Stress Scale; DCM, Dementia Care Managers; DT, Dignity Therapy; ESD, Early Stage Dementia; GAI, Geriatric Anxiety Inventory; GDS-15, Geriatric Depression Scale; HADS, Hospital Anxiety and Depression Scale; HHI, Herth Hope Index; IADL (Lawton), Instrumental Activities of Daily Living according to the Lawton Scale; LivDem, Living well with Dementia; MATS, Memory Assessment and Treatment Service; MADRS, Montegomery and A˚sberg Depression Rating Scale; MBI, Mindfulness-Based Intervention; MCI, Mild Cognitive Impairment; MM, Marte Meo®; MMSE, Mini-Mental State Examination; MRP, Multicomponent Rehabilitation Programme; NPI-Q, Neuro Psychiatric Inventory Questionnaire; PCTS, Preferences for Care Tasks Scale; PDI, Patient Dignity Inventory; PIN, Present in the Now; PLwD, People Living with Dementia; PSQI, Pittsburg Sleep Quality Inventory; PSS-13, the Perceived Stress Scale; QIs, Quality Indicators; QoL- AD, Quality of Life in Alzheimer's Disease; QoL, Quality of Life; RAID, Rating Anxiety in Dementia Scale; RBANS, Repeatable Battery for the Assessment of Neuropsychological Status; RMPBC, Revised Memory Problem and Behaviour Checklist; RR, Respiratory Rate; SF-36, Short Form Health Survey; SRF, Session Rating Form; SHARE, Support, Health, Activities, Resources, and Education; TAU, Treatment as Usual; UAE, United Arab Emirates; UCLA, the University of California at Los Angeles; YOD, Young-Onset Dementia; ZARIT-BI, Zarit Burden Interview
