# Supplementary material for: The role and impact of therapeutic counselling on the emotional experience of adults living with dementia: A systematic review
Source: Dementia (London). 2024 Apr 16;23(5):882–902. doi: 10.1177/14713012241233765 (PMC11163847; doi:10.1177/14713012241233765)
Supplement: Supplemental Material - The role and impact of therapeutic counselling on the emotional experience of adults living with dementia: A systematic review [file sj-pdf-8-dem-10.1177_14713012241233765.pdf]

Counselling Adults with Dementia: a review of on the role and impact of therapeutic counselling on the emotional experience of adults with dementia
TABLE 7: Characteristics Systematic Reviews

Author/ Date/ Country	No. of studies	Participants	Methods	Review Aim	Design of included studies	Study Interventions and focus	Main concepts/themes	Recommendations
Bielsten, T. 2019 Sweden	Six studies Two studies exploring counselling interventions (n=2) included (Part A) Extension of review (Part B)	People Living with Mild – Moderate stage Dementia and carer dyads (n= 92)<br><br>Auclair et. al (2009) (n=42) looked at individualised support to couple's needs related to the changes that had occurred since the onset of Alzheimer's Disease (AD)<br><br>Quayhagen et al. (2000) (n=50) The dyadic counselling component addressed conflicts and conflict resolution, stress, anger management and communication. Dyadic counselling was expected to show improvement in physical and emotional well-being and marital	Database searches x 2 (A and B) (n=6) Academic Search Premier, CINAHL, PsycINFO, PubMed, Scopus and Web of Science between January 2000 to August 2017	To explore the 'what' and 'why' of interventions aimed at couples where one partner has a diagnosis of dementia and in which the couple jointly participate.<br><br>1. What types of interventions have been conducted for couples living with dementia? (What?) 2. What were the objectives and outcome measures of the interventions? (Why?)	2000-2012 RCTs (n=4); Single group (n=2)<br><br>Auclair et al. RCT & Qualitative<br><br>Quayhagen et al. RCT<br><br>Garcia-Alberta et al. Single group intervention<br><br>Stanley et al. Pilot RCT	Objectives related to: cognitive function for the person living with dementia (PLwD); and, for the care partner, to well-being.<br><br>Auclair et al. To preserve the integrity of the relationship and the sense of self of each partner.<br><br>Quayhagen et al. To improve caregivers physical and emotional well-being and marital interaction.<br><br>Garcia-Alberta et al. Reduce depression in depressed people with dementia.<br><br>Stanley et al.	The two counselling studies showed beneficial impacts in respect of the spousal relationship and empathised the importance of couples to adopt an accepting, non-judgmental, non-blaming attitude towards each other.<br><br>This contrasted to overall findings which highlighted that joint interventions for PLwD and care partners lack a salutogenic and genuine dyadic approach where both partners' views of their relationship, their strengths and resources are identified, valued and supported.<br><br>The focus on relationship dynamics was	Future interventions would benefit from using a framework of relationship-centred care, in which the person with the dementia is the central concern but cannot be supported isolated from the impact of relationships. Interventions should include more components of self-management that aim to live well with (or despite) a chronic condition, which, in turn, could contribute towards a shift in focus from illness-specific to resource-oriented approaches in support of couples where one partner has a dementia.<br><br>Many dyadic interventions which include couples in which one partner has a dementia lack a relational understanding.

Author/ Date/ Country	No. of studies	Participants	Methods	Review Aim	Design of included studies	Study Interventions and focus	Main concepts/themes	Recommendations
		interaction for care partners.<br><br>Additional studies in Part B:<br>Garcia-Alberta et al. (Dyads=9) Cognitive Behavioural Therapy (CBT)<br><br>Stanley et al. (Dyads=32) CBT				To reduce anxiety in dementia	generally found to be vague with the views of PLwD, often excluded. A ‘negative approach’ is accorded to outcome measures in dementia which may adversely impact couples relationships and their views of their situation.<br>Part B:<br>Joint dyadic interventions promote opportunities for interaction and shared activity but key areas of challenge identified. In line with the findings of part A there is a negative approach to outcomes, lack of a genuine dyadic approach for relationship sustaining care; lack of tailored support, neglect of interpersonal issues; the overlook of the views of people with dementia; and, a lack	Relationship factors need to be addressed and the voice of people with dementia should be obvious in the context of dyadic joint interventions

Author/ Date/ Country	No. of studies	Participants	Methods	Review Aim	Design of included studies	Study Interventions and focus	Main concepts/themes	Recommendations
							of easily-accessible support.	
Cheston, R.<br>2017<br>England	24 from 26 studies	People living with mild-mod dementia<br>n= (1,065)	Systematic Review - Electronic database search- Cinahl Plus, the Cochrane Library, Embase, Medline and Psychinfo - and reference lists for records of psychotherapy with people affected by Alzheimer's Disease, Vascular dementia, Lewy-body dementia or a mixed condition between 1 January 1997 and 31 March 2015	Review the existing evidence base for individual and group psychotherapy with people affected by dementia	Randomised and non-randomised controlled trials and studies using repeated measured designs.<br><br>19 articles concerning 16 studies were identified as Level I (RCTs, four of which adequately powered); 2 as Level II (controlled non-randomised studies) and 5 as Level III (repeated measure designs)	Studies of therapies which met British Association for Counselling and Psychotherapy definitions: CBT (n=6); Person Centred Counselling (n=3); Psychodynamic Interpersonal (n= 2); Validation therapy (n=4); Generic group psychotherapy (n=5); Multi-component therapy (n=4)	Limited and uncertain evidence on the use of Psychotherapy to help people affected by dementia to adjust to their illness.<br>Strongest evidence supported the use of short-term group therapy after diagnosis and an intensive, multi-faceted intervention for Nursing Home residents.	Many areas of Psychotherapy need further research.<br>There is a need to identify the change processes & nuanced elements of psychotherapeutic interventions that lead to successful outcomes. The importance of promoting therapeutic skills/understanding for all those involved in dementia care is emphasised.
Jao, Y.-L.<br>2017<br>USA	18	Early-stage dementia<br>(n=c.350)	Database Search (n=7)<br>PubMed,	To update current evidence on the use and	Quantitative (n=11) and qualitative (n=7)	Different forms of group support. All involved some	Support groups showed numerous beneficial outcomes: positive impacts on	Evidence on support groups for individuals with ESD is insufficient

Author/ Date/ Country	No. of studies	Participants	Methods	Review Aim	Design of included studies	Study Interventions and focus	Main concepts/themes	Recommendations
			CINAHL, AgeLine, Cochrane Library, PsycINFO, ProQuest, and Scopus Narrative analysis	effects of support groups for individuals with Mild Cognitive Impairment (MCI) and Early Stage Dementia (ESD) and their care partners. The term 'support group' was defined as a group treatment program that provided any of the following: (a) emotional support (opportunities to express feelings and concerns), (b) peer support, (c) education about MCI or dementia, or (d) a combination of these approaches (Schmall, 1984)	RCTs (n=4), non-RCTs (n=2), single group pretest–posttest intervention studies (n=3), one single group post-test-only follow-up study, and one descriptive study.	level of emotional and peer support and 12 of the studies also included educational support. Most were community based and one online.	participant acceptance of cognitive impairment; performance and satisfaction of meaningful activity; resilience; self-help; and care partner coping self-efficacy, perceived support, and preparation and task effectiveness. Support groups were well accepted by participants and care partners but those with a focus on educational support may potentially result in negative affect. Lack of ethnic diversity.	to draw robust conclusions. Large-scale studies are needed to confirm the effects of support groups for individuals with ESD in all populations
National Institute for Health and	3	125	Commissioned Evidence	Research Q. How effective are pre, peri &	Extensive review of the current	Pre, peri & post-diagnostic counselling and	Moderate-quality evidence found a clinically meaningful	The committee noted that there were no meaningful

Author/ Date/ Country	No. of studies	Participants	Methods	Review Aim	Design of included studies	Study Interventions and focus	Main concepts/themes	Recommendations
Care Excellence (2018) England			Review of well-designed studies (RCTs) covers diagnostic counselling and psychotherapy.	post-diagnostic counselling and support on outcomes for people living with dementia and their families?<br><br>What are the most effective non-pharmacological interventions for managing illness emergent non-cognitive symptoms, such as psychosis, depression, behavioural changes in people living with dementia?	evidence Includes RCTs prior to 2015 and evidence from support groups	support/ non-pharmacological interventions for managing illness emergent non-cognitive symptoms	post-intervention improvement in depressive symptoms in people living with dementia offered psychotherapy versus usual care, but these effects did not persist at long-term follow-up.<br>Low- to moderate-quality evidence from up to 2 RCTs containing 95 participants could not detect clinically meaningful differences in cognition, activities of daily living, or quality of life between people living with dementia offered psychotherapy versus usual care.	benefits found in trials of psychotherapy (specifically interpersonal therapy) or on pre- and peri-diagnostic counselling and support for people living with dementia and their families.<br>It was proposed to make 'do not offer' recommendations for these interventions even in the absence of proven clinical harm, as the committee perceived that money spent on these interventions would be better used on interventions with evidence of benefits.
Noone, D. 2019 England	Three studies from eight	PLwD (n=129) that met criteria for dementia according to the DSM-IV or International Classifictn of Diseases-10 (ICD-10),<br>Prtcpnts also met	Database search (n=3) OvidMedline; PsychInfo; Embase.<br><br>Studies rated for	Assess the effectiveness of psychosocial interventions for depression and anxiety in people living with dementia (PLwD) or mild	RCT (n=8) using control group; including treatment as usual (TAU), waitlist controls or a comparison intervention.	Problem Adaptation Therapy PAT (Kiosses et al 2015);<br>Cognitive behavioural therapy (CBT) (Spector et al 2015);	Two RCTs found that psychotherapeutic interventions (multicomponent intervention, PAT) were effective at reducing symptoms of depression in PLwD who were depressed.<br>CBT reduced	High quality studies with larger sample sizes are required to test the efficacy of specific interventions such as CBT.

Author/ Date/ Country	No. of studies	Participants	Methods	Review Aim	Design of included studies	Study Interventions and focus	Main concepts/themes	Recommendations
		criteria for either clinical depression or anxiety.<br>Recruited from nursing homes, research centres and outpatient services<br>Mean age = 82yrs	quality using Cochrane's Risk of Bias tool<br><br>Meta-analyses	cognitive impairment (MCI)	Studies included if they reported at least one standardised measure of either depression or anxiety.	Multi-component Intervention (Bailey et al., 2016).	symptoms of anxiety in PLwD who were anxious and was sustained at 3-6 month follow up.	
Orgeta, V.<br>2015<br>England	6	Older adults diagnosed with dementia, Alzheimer's disease or organic brain syndrome, according to the DSM-IV, ICD-10 or comparable and participants with a diagnosis of MCI (n=439), in any setting.<br>Mostly mild dementia but no figures separating PLwD and MCI	Systematic review and meta-analysis<br><br>Cochrane Dementia and Cognitive Improvement Group's Specialized Register and major healthcare databases including MEDLINE, Embase, CINHAL, PsycINFO, ALIOS and LILACS Grey literature search/trials ref search and ref list search<br>2	To evaluate the evidence of effectiveness of psychological treatments in treating depression and anxiety in people with dementia and MCI.	Randomised controlled trials (RCTs) with primary outcomes: symptoms of anxiety and depression; and, secondary outcomes: quality of life; ability to perform daily activities; neuropsychiatric symptoms; cognition; caregivers' self-rated depressive symptoms	Psychological treatment vs usual care in people with dementia and MCI.<br><br>Trials used cognitive–behavioural therapy, interpersonal therapy, counselling or multimodal interventions including a specific psychological therapy.	Psychological treatments are effective in reducing symptoms of depression and anxiety for people with dementia.	Highlights a need for high-quality, multicentre trials including standardised, well-defined interventions

Author/ Date/ Country	No. of studies	Participants	Methods	Review Aim	Design of included studies	Study Interventions and focus	Main concepts/themes	Recommendations
			reviewers working independently					
Shoesmith, E.<br>2020<br>England	Four studies	PLwD (n=80)	Database search of MEDLINE (via PubMed), PsycINFO, and CINAHL<br><br>2-author review Adapted CASP quality scoring<br><br>Narrative synthesis	to examine the following research questions: (1) Are counselling/psychotherapeutic interventions effective for people with dementia?, (2) Are counselling/psychotherapeutic interventions effective for care-givers of people with dementia? and (3) Which modes of delivery are most effective for people with dementia and care-givers of people with dementia?	RCT or Quasi-Exprmntl (n=20) single-group repeated measures (n=4) Qualitative (n=8)	Cognitive (Scholey and Woods, 2003); psycho-dynamic (Burns et al., 2005) and person-centred (Tappen and Williams, 2009). Carpenter et al. (2002) delivered R-E-M (Restore–Empower–Mobilise) Psychotherapy – combination of different psychological approaches)	Variation in counselling/psychotherapeutic approaches and modes of delivery. Most interventions adopted either a problem-solving or CBT approach with mixed effectiveness in treating depression and other symptoms. Need for modifications emphasised, e.g. simplifying materials and shifting focus to behavioural components to accommodate the cognitive needs of a person with dementia. Face to face delivery bigger impact on outcomes	Interventions need to be modified/ tailored to the experiences, level of cognitive impairment and background of participants; understanding dyadic relationship essential for effectiveness; better reporting of CI needed and understanding of its impact on mood training/guidance for practitioners delivering interventions; Need for research into Psychodynamic Therapy
Tay, K. W.<br>2019<br>Malaysia	11	116	Database Review (n=3)	to clarify whether CBT can be used to reduce depression and anxiety symptoms in	RCT (n=2) pilot study level -included a range of formats, such as single case studies and	Dyad (n=9)<br>Group (n=1)<br>Individual (n=1)<br>With multiple caregivers (n=1)	Need for customised modifications based on the cognitive abilities and needs of PLwD<br>Attrition high<br>Questions the	CBT is feasible and effective at reducing anxiety and depression symptoms among patients with mild-moderate stage

Author/ Date/ Country	No. of studies	Participants	Methods	Review Aim	Design of included studies	Study Interventions and focus	Main concepts/themes	Recommendations
				persons with dementia	quasi-experimentl design		feasibility of CBT in PLwD who have a CDR score of ≥2 MMSE ≥15<br>No recognised standard for timing/duration etc.<br>Limited outcome measures.<br>Mainly people with mild-mod dementia<br>Caregiver inclusion (mental health status needs to be assessed)	dementia but the evidence is at an early stage.<br>Modifications of CBT, such as spaced retrieval and prioritisation of behavioural strategies, need to be studied.<br>Caregiver inclusion advocated.

References:

BIELSTEN, T. & HELLSTRÖM, I. 2019. A review of couple-centred interventions in dementia: Exploring the what and why – Part A. *Dementia,* 18**,** 2436-2449.

CHESTON, R. & IVANECKA, A. 2017. Individual and group psychotherapy with people diagnosed with dementia: a systematic review of the literature. *Int J Geriatr Psychiatry,* 32**,** 3-31.

NATIONAL INSTITUTE FOR HEALTH AND CARE EXCELLENCE 2018. Dementia: Assessment, management and support for people living with dementia and their carers. England: NICE UK.

NOONE, D., STOTT, J., AGUIRRE, E., LLANFEAR, K. & SPECTOR, A. 2019. Meta-analysis of psychosocial interventions for people with dementia and anxiety or depression. *Aging & Mental Health,* 23**,** 1282-1291.

ORGETA, V., QAZI, A., SPECTOR, A. & ORRELL, M. 2015. *Psychological treatments for depression and anxiety in dementia and mild cognitive impairment: Systematic review and meta-analysis*, The British Journal of Psychiatry.

SHOESMITH, E., GRIFFITHS, A. W., SASS, C. & CHARURA, D. 2020. Effectiveness of counselling and psychotherapeutic interventions for people with dementia and their families: a systematic review. *Ageing and Society***,** 1-28.

TAY, K. W., SUBRAMANIAM, P. & OEI, T. P. 2019. Cognitive behavioural therapy can be effective in treating anxiety and depression in persons with dementia: a systematic review. *Psychogeriatrics:The Official Journal of the Japanese Psychogeriatric Society,* 19**,** 264-275.

List of Abbreviations:

AD, Alzheimer's Disease; CBT, Cognitive-Behavioural Therapy; DSM-IV, Diagnostic and Statistical Manual of Mental Disorders; ESD, Early Stage Dementia; ICD-10, International Classification of Diseases 10th Revision; MCI, Mild Cognitive Impairment; MMSE, Mini-Mental State Examination; PAT, Problem Adaptation Therapy; PLwD, People Living with Dementia; RCT, Randomised Controlled Trial; TAU, Treatment as Usual
