# Supplementary material for: The role and impact of therapeutic counselling on the emotional experience of adults living with dementia: A systematic review
Source: Dementia (London). 2024 Apr 16;23(5):882–902. doi: 10.1177/14713012241233765 (PMC11163847; doi:10.1177/14713012241233765)
Supplement: Supplemental Material - The role and impact of therapeutic counselling on the emotional experience of adults living with dementia: A systematic review [file sj-pdf-9-dem-10.1177_14713012241233765.pdf]

Table 9. List of Recommendations

- Create opportunities for people with dementia to take part in therapeutic counselling
- Ensure public involvement throughout the research and design process
- Increase the use of qualitative research to explore and report on participant experience
- Adapt or tailor therapeutic methods to meet the specific characteristics and needs of the person with dementia, taking any other diagnoses into account
- Provide ongoing support for therapists including customised training and supervision, peer learning communities and adequate resources
